# Conventional and genetic evidence on alcohol and vascular disease aetiology: a prospective study of 500 000 men and women in China

**DOI:** 10.1016/S0140-6736(18)31772-0

**Published:** 2019-05-04

**Authors:** Iona Y Millwood, Robin G Walters, Xue W Mei, Yu Guo, Ling Yang, Zheng Bian, Derrick A Bennett, Yiping Chen, Caixia Dong, Ruying Hu, Gang Zhou, Bo Yu, Weifang Jia, Sarah Parish, Robert Clarke, George Davey Smith, Rory Collins, Michael V Holmes, Liming Li, Richard Peto, Zhengming Chen

**Affiliations:** a Medical Research Council Population Health Research Unit, Nuffield Department of Population Health, University of Oxford, Oxford, UK; b Clinical Trial Service Unit and Epidemiological Studies Unit, Nuffield Department of Population Health, University of Oxford, Oxford, UK; c Chinese Academy of Medical Sciences, Beijing, China; d Gansu Provincial Center for Disease Control and Prevention, Lanzhou, China; e Zhejiang Provincial Center for Disease Control and Prevention, Hangzhou, China; f Henan Center for Disease Control and Prevention, Zhengzhou, China; g Nangang Center for Disease Control and Prevention, Harbin, China; h Liuyang Center for Disease Control and Prevention, Changsha, China; i Medical Research Council Integrative Epidemiology Unit, Population Health Sciences, University of Bristol, Bristol, UK; j Department of Epidemiology and Biostatistics, Peking University Health Science Center, Peking University, Beijing, China

## Abstract

**Background:**

Moderate alcohol intake has been associated with reduced cardiovascular risk in many studies, in comparison with abstinence or with heavier drinking. Studies in east Asia can help determine whether these associations are causal, since two common genetic variants greatly affect alcohol drinking patterns. We used these two variants to assess the relationships between cardiovascular risk and genotype-predicted mean alcohol intake in men, contrasting the findings in men with those in women (few of whom drink).

**Methods:**

The prospective China Kadoorie Biobank enrolled 512 715 adults between June 25, 2004, and July 15, 2008, from ten areas of China, recording alcohol use and other characteristics. It followed them for about 10 years (until Jan 1, 2017), monitoring cardiovascular disease (including ischaemic stroke, intracerebral haemorrhage, and myocardial infarction) by linkage with morbidity and mortality registries and electronic hospital records. 161 498 participants were genotyped for two variants that alter alcohol metabolism, *ALDH2*-rs671 and *ADH1B*-rs1229984. Adjusted Cox regression was used to obtain the relative risks associating disease incidence with self-reported drinking patterns (conventional epidemiology) or with genotype-predicted mean male alcohol intake (genetic epidemiology—ie, Mendelian randomisation), with stratification by study area to control for variation between areas in disease rates and in genotype-predicted intake.

**Findings:**

33% (69 897/210 205) of men reported drinking alcohol in most weeks, mainly as spirits, compared with only 2% (6245/302 510) of women. Among men, conventional epidemiology showed that self-reported alcohol intake had U-shaped associations with the incidence of ischaemic stroke (n=14 930), intracerebral haemorrhage (n=3496), and acute myocardial infarction (n=2958); men who reported drinking about 100 g of alcohol per week (one to two drinks per day) had lower risks of all three diseases than non-drinkers or heavier drinkers. In contrast, although genotype-predicted mean male alcohol intake varied widely (from 4 to 256 g per week—ie, near zero to about four drinks per day), it did not have any U-shaped associations with risk. For stroke, genotype-predicted mean alcohol intake had a continuously positive log-linear association with risk, which was stronger for intracerebral haemorrhage (relative risk [RR] per 280 g per week 1·58, 95% CI 1·36–1·84, p<0·0001) than for ischaemic stroke (1·27, 1·13–1·43, p=0·0001). For myocardial infarction, however, genotype-predicted mean alcohol intake was not significantly associated with risk (RR per 280 g per week 0·96, 95% CI 0·78–1·18, p=0·69). Usual alcohol intake in current drinkers and genotype-predicted alcohol intake in all men had similarly strong positive associations with systolic blood pressure (each p<0·0001). Among women, few drank and the studied genotypes did not predict high mean alcohol intake and were not positively associated with blood pressure, stroke, or myocardial infarction.

**Interpretation:**

Genetic epidemiology shows that the apparently protective effects of moderate alcohol intake against stroke are largely non-causal. Alcohol consumption uniformly increases blood pressure and stroke risk, and appears in this one study to have little net effect on the risk of myocardial infarction.

**Funding:**

Chinese Ministry of Science and Technology, Kadoorie Charitable Foundation, National Natural Science Foundation of China, British Heart Foundation, Cancer Research UK, GlaxoSmithKline, Medical Research Council, and Wellcome Trust.

## Introduction

Although high alcohol intake is dangerous,^1^ in many prospective studies moderate intake (about one to two drinks per day, or 100 g of alcohol per week) is associated with somewhat lower incidence of stroke and myocardial infarction than no alcohol intake.^2^, ^3^, ^4^, ^5^, ^6^ However, these apparently protective associations do not necessarily mean that moderate alcohol intake itself is protective against either condition. For, poor health might affect alcohol consumption (reverse causality), and other systematic differences might exist between people with different drinking patterns that were not fully allowed for (residual confounding).^7^, ^8^ Small, short-term randomised trials^9^, ^10^, ^11^ have shown that alcohol intake causes changes in physiological factors such as blood pressure, HDL cholesterol, adiponectin, and fibrinogen, but such trials cannot reliably assess realistic effects on the incidence of stroke or myocardial infarction.

**Research in context**

**Evidence before this study**

Although conventional epidemiological studies have associated moderate alcohol intake with a reduced risk of stroke and, particularly, coronary heart disease, these apparently protective effects may be largely non-causal. Since genetic variants have only limited effects on alcohol intake in populations of European descent, studies in such populations cannot directly compare the effects of moderate alcohol intake with those of near-total alcohol avoidance. The question of whether moderate intake really is protective can be addressed by genetic epidemiology in east Asian populations where two common genetic variants (*ALDH2*-rs671 and *ADH1B*-rs1229984) jointly cause large absolute differences in mean alcohol intake.

To review the genotypic evidence about the relationships between vascular disease and these two genetic variants, we searched PubMed from database inception to Jan 11, 2019, for studies that had investigated the effects of either variant on alcohol exposure or cardiovascular disease, using the search terms (“alcohol” or “blood pressure” or “cholesterol” or “cardiovascular” or “stroke” or “myocardial infarction” or “coronary”) and ([“ADH1B” or “ALDH2” or “rs1229984” or “rs671”] or [(“East Asian” or “Chinese” or “Japanese” or “Korean”) and “genome-wide”]).

In meta-analyses, the incidence of coronary heart disease appears to be increased by the rs1229984 genotype that increases alcohol exposure, but, conversely, may be decreased by the rs671 genotype that increases alcohol exposure. There is insufficient evidence about the effects of these two genetic variants on stroke.

**Added value of this study**

Our large prospective study is of a population where both variants are common but, as few women drink, strongly affect alcohol exposure in only one sex. To help assess causal effects of alcohol on the incidence of ischaemic stroke, haemorrhagic stroke, and myocardial infarction, it compares the findings from conventional epidemiological analyses and from genetic analyses (which use Mendelian randomisation to study the effects of genetic variants that strongly influence mean male alcohol intake). Consistent with previous studies, the conventional analyses among men found U-shaped associations of self-reported alcohol intake with stroke and myocardial infarction, with the lowest risks at moderate intake.

In contrast, although genotype-predicted mean male alcohol intake varied from near zero to about four drinks per day, it did not have U-shaped associations with risk, and was strongly positively associated throughout its range with blood pressure, ischaemic stroke, and haemorrhagic stroke, suggesting no substantial protective effect of moderate alcohol intake. Based on a smaller number of cases, genotype-predicted mean male alcohol intake had no apparent association with myocardial infarction.

Among women, alcohol intake was low and these genetic variants had little effect on stroke or on myocardial infarction, suggesting that they did not have any substantial non-alcohol-mediated (ie, pleiotropic) net effect on these diseases.

**Implications of all the available evidence**

Genetic evidence shows that the apparently protective effects of moderate alcohol intake against stroke are not mainly caused by alcohol itself, and are largely artifacts of reverse causation and confounding. Throughout the range of mean intake that we studied by Mendelian randomisation, increasing mean alcohol intake uniformly increases blood pressure and stroke incidence. Among the men in this population, alcohol was responsible for about 8% of ischaemic strokes and 16% of intracerebral haemorrhages. The effects of alcohol on myocardial infarction are less certain.

The question of whether there are any real protective effects of moderate alcohol intake can be investigated genetically (by Mendelian randomisation),^12^, ^13^ particularly in populations where there are common genetic variants that alter alcohol metabolism but, since few women drink, strongly influence alcohol intake only in men. Alcohol is metabolised to acetaldehyde, which can cause discomfort if too much accumulates before it is broken down (panel, table 1), and there are genetic variants that importantly affect acetaldehyde formation and breakdown.^14^

Panel **Alcohol, acetaldehyde, and the east Asian flushing syndrome**

$$\text{Alcohol}\overset{\text{ADH}}{⟶}\text{acetaldehyde}\overset{\text{ALDH}}{⟶}\text{acetate}$$

The major clearance pathway for blood alcohol is that an alcohol dehydrogenase (ADH), mainly ADH1, oxidises it to acetaldehyde, which causes discomfort at sufficient concentrations. An aldehyde dehydrogenase (ALDH), mainly ALDH2, then detoxifies the acetaldehyde (oxidising it to acetate, which does not cause discomfort). Fast clearance of alcohol or, particularly, slow breakdown of acetaldehyde can cause individuals to limit alcohol intake.

In African and European populations, acetaldehyde is broken down quickly enough to maintain tolerably low concentrations in drinkers. In east Asian populations, there is a common loss-of-function variant of the *ALDH2* gene on chromosome 12 (rs671). Even a single copy decreases acetaldehyde breakdown enough for the concentration to become uncomfortably high after drinking alcohol. This variant is an important determinant of the east Asian flushing reaction to alcohol, and of alcohol intake. Less importantly, a genetic variant of the *ADH1B* gene on chromosome 4 (rs1229984) that is common in east Asia increases alcohol clearance rates. Together, these two single nucleotide polymorphisms strongly affect alcohol exposure (table 1), and each has been shown to decrease substantially the incidence of alcoholism.

Both variants involve a G→A mutation, with the A allele decreasing alcohol exposure. Each variant has three possible genotypes, AA, AG, and GG, so the two variants define nine possible genotypes. Alcohol intake is affected more by the decreased rate of breakdown of acetaldehyde than by the increased alcohol clearance rate. Hence, when describing these nine genotypes the rs671 genotype is given first and the rs1229984 genotype second, and alphabetic order corresponds to increasing alcohol intake: AA/AA, AA/AG, AA/GG; then AG/AA, AG/AG, AG/GG; then GG/AA, GG/AG, GG/GG.

**Table 1:** Two east Asian genetic variants that alter alcohol metabolism

		***ALDH2* gene**	***ADH1B* gene**
Enzyme*		ALDH2, an aldehyde dehydrogenase	ADH1, an alcohol dehydrogenase
Enzyme function		Acetaldehyde breakdown, by oxidation to acetate	Alcohol breakdown, by oxidation to acetaldehyde
Description of variants			
	SNP identifier	rs671	rs1229984
	Nucleotide change	G→A	G→A
	Amino acid change†	Glu504→Lys	Arg48→His
	Enzyme activity change	Decreased substantially	Increased substantially
	Alcohol clearance rate	Unaffected	Accelerated
	Acetaldehyde clearance rate	Decreased substantially	Unaffected
	Alcohol intake	Reduced substantially	Reduced‡

ALDH=aldehyde dehydrogenase. ADH=alcohol dehydrogenase. SNP=single nucleotide polymorphism.

* ALDH2 is a tetramer of the ALDH2 gene product that requires all four parts to be functional, so a loss-of-function variant is nearly dominant. ADH1 is a dimer that requires two functional parts from the products of any of three similar genes, *ADH1A, ADH1B*, and *ADH1C*.

† For ALDH2 and ADH1B, each of these amino acid changes can be described as altering the “*1” into the “*2” enzyme isoform.

‡ The *ADH1B*-rs1229984 east Asian variant is nearly dominant, with AA and AG having similar effects on alcohol intake.

The *ALDH2*-rs671 variant, which is common only in east Asian populations, greatly slows acetaldehyde breakdown, and the resulting accumulation of acetaldehyde can cause severe discomfort that strongly reduces alcohol intake. Studies of this variant have confirmed the trial evidence that alcohol causally affects blood pressure and some other physiological traits.^15^, ^16^, ^17^ A less important genetic variant, *ADH1B*-rs1229984, accelerates alcohol conversion to acetaldehyde and reduces alcohol intake.

Using data from the nationwide China Kadoorie Biobank prospective study, we investigated the causal relationships between alcohol and cardiovascular disease by comparing the findings from conventional epidemiology (classifying people by self-reported intake) and from genetic epidemiology (using these two variants to classify people by genotype-predicted mean alcohol intake). Since few Chinese women drink, these genetic variants can be used to predict large absolute differences in mean alcohol intake in men, but not in women. Hence, if alcohol itself substantially affects the incidence of stroke or myocardial infarction then these two genetic variants should affect disease incidence differently in men and in women.

## Methods

### Study design and participants

The China Kadoorie Biobank^18^ is a prospective cohort study of 512 715 adults recruited between June 25, 2004, and July 15, 2008, from ten diverse rural and urban areas of China. All permanent residents (aged 35–74 years) without known major disabilities were to be invited into the baseline survey, and 499 500 (28% of 1 801 167 invitees) participated, along with 13 215 who were just outside the target age range. Ethics approval was obtained from relevant local, national, and international ethics committees, and all participants provided written informed consent.

At baseline, participants attended survey clinics where interviewers using laptop-based questionnaires recorded socioeconomic status, medical history, smoking, drinking, diet, and physical activity. Measurements included height (sitting and standing), weight, waist and hip circumference, heart rate, and blood pressure. A non-fasting blood sample was collected for long-term storage. Similar procedures were followed at two separate resurveys of 4–5% of all participants (resurveying 19 786 between May 26, 2008, and Oct 10, 2008, and 25 041 between Aug 4, 2013, and Sept 18, 2014).

### Alcohol drinking patterns

Past and current alcohol drinking patterns were self-reported.^19^ Participants were classified as current drinkers (some alcohol use in most weeks in the past year), non-drinkers (no alcohol use in the past year and never drank in most weeks), occasional drinkers (occasional alcohol use in the past year but never drank in most weeks), or ex-drinkers (none or occasional alcohol use in the past year but previously drank in most weeks). In current drinkers, baseline alcohol intake (from main beverage type, amount, and frequency) was subclassified according to sex: for men, the groupings were less than 140, 140–279, 280–419, and 420 or more g per week and for women, less than 70 and 70 or more g per week. For each of these baseline-defined groups the usual alcohol intake was estimated from intake at the resurveys. Further details of alcohol assessment are described in the appendix (p 4).

### Follow-up for incident cardiovascular disease

Incident cardiovascular disease and cause-specific mortality were ascertained through ongoing linkage, via the unique national identification number, to electronic hospital records from the nationwide health insurance system (which has >98% coverage across the ten study areas), to established local registries of stroke and coronary heart disease, and to local death registries. The insurance records included information about every hospital admission, and evidence of cardiovascular disease was reviewed and coded according to the International Classification of Diseases, tenth revision. The diseases analysed were ischaemic stroke, intracerebral haemorrhage, total stroke, acute myocardial infarction, and total coronary heart disease (first non-fatal or fatal record of each; appendix p 4).

### Genotyping and biochemistry

161 498 participants were genotyped for the two variants of interest, rs671 and rs1229984, using custom Illumina Golden Gate or Affymetrix Axiom arrays at BGI (Shenzhen, China). They included 151 028 randomly selected participants (used in all genetic analyses) and an additional 10 470 participants (used only in analyses of stroke and coronary heart disease) who had been selected because during follow-up they had had a stroke or coronary heart disease recorded.

18 256 participants (who had been selected for nested case-control studies of stroke and of coronary heart disease) were assayed for plasma HDL cholesterol, LDL cholesterol, triglycerides, lipoprotein(a), C-reactive protein, fibrinogen, and γ-glutamyl transferase at the Wolfson Laboratory (Clinical Trial Service Unit, Oxford, UK). Of them, 98% (17 874) were among those genotyped for the above two variants. Further details of genotyping and biochemistry are in the appendix (p 4).

### Mean alcohol intake by genotype and area

**Figure 1:**
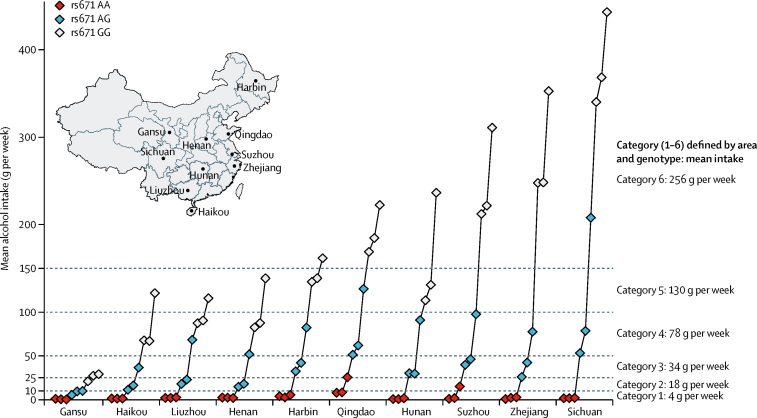
Mean alcohol intake in men from ten study areas in China, subdivided by nine possible genotypes of two common variants that alter alcohol metabolism For each genotype, the A allele discourages alcohol consumption. Within each area, mean alcohol intake was plotted according to the nine possible *ALDH2*-rs671 and *ADH1B*-rs1229984 genotypes (each AA, AG, or GG) from AA/AA homozygosity for both variants to GG/GG homozygosity for both variants. Alcohol intake thresholds were defined at 10, 25, 50, 100, and 150 g per week to assign individuals into six categories of mean male alcohol intake on the basis of their genotype and area.

Combinations of the genotypes for the two genetic variants of interest (rs671 and rs1229984; each AA, AG, or GG) define nine genotypes (panel). Since alcohol use varies greatly by study area,^19^ in each of the ten study areas mean male alcohol intake was calculated for each of these nine genotypes (assigning occasional drinkers an intake of 5 g per week and excluding ex-drinkers, which effectively assigns them the mean intake in other participants in their area). The 90 combinations of genotype and area were subdivided into six categories (1–6) according to these 90 mean values, with cutoff points of 10, 25, 50, 100, and 150 g per week (figure 1). Classification of individual participants into the six categories was dependent only on their genotype and study area, not on their individual drinking patterns. Women were classified into the same six categories of genotype and area as men, regardless of their drinking patterns, to facilitate comparison between genotypic effects in men and in women.

### Statistical analysis

General linear models were used to assess associations with continuous variables (eg, systolic blood pressure), giving the adjusted mean values in each of several exposure groups. These exposure groups were defined either by self-reported alcohol drinking patterns or by categories of genotype and area. Cox regression was used to assess associations with disease rates, giving the relative risks (RRs) of disease incidence in each exposure group, after specifying one of these as the reference group (RR=1). The variance of the log risk in each group, including the reference group, was calculated (from the variances and covariances of the log RRs in all groups except the reference group) and used to obtain group-specific 95% CIs.^20^ Participants with a previous history of coronary heart disease, stroke, or transient cerebral ischaemia were excluded from the analyses of disease incidence. Analytic models and sensitivity analyses are described in the appendix (pp 5, 6).

Conventional epidemiological analyses related individual drinking patterns to physiological factors, or to the RRs for disease. They were adjusted for area, age, education, income, and smoking. To avoid the regression dilution bias,^21^ among current drinkers the means or log RRs were plotted against the usual alcohol intake, along with straight lines of best fit. The slopes of these lines were described in terms of the change in the mean, or in the RR, per 280 g per week (ie, around four drinks per day) usual alcohol intake. Occasionally the RR per 100 g per week was also calculated, as described in the appendix (p 6).

Genetic epidemiological analyses related genotype and study area (categorised as described above) to physiological factors, or to the RRs for disease. These analyses were stratified by area (so any differences in outcome between the six categories of genotype and study area reflect purely genotypic effects) and were adjusted only for age (although sensitivity analyses also adjusted them for education, income and smoking). Means or log RRs were plotted against the mean alcohol intake among the men in each of the six categories. Within each study area a similar analysis was done, and then the slope of the straight line of best fit related outcome to exposure in that one area. To obtain a meta-analysis of these within-area slopes (each reflecting purely genotypic effects), an inverse-variance weighted mean of them yielded the overall slope, stratified by area. Slopes were described in terms of the change in the mean, or in the RR, per change of 280 g per week in genotype-predicted mean male alcohol intake.

Genotypic analyses in women were done not to assess the effects of alcohol in women (since women's alcohol intake was known to be minimal), but to determine the extent to which the genotypes studied in men had pleiotropic effects^12^ (ie, effects of the genotype that were not mediated by drinking patterns). Hence, we used the same six categories of genotype and area as in men, relating the area-adjusted genotypic effects in women to the mean male alcohol intake in these six categories.

For men and for women we also calculated as sensitivity analyses (stratified by area and adjusted for age) the separate genotypic effects of each of the two variants. SAS (version 9.3) and R (version 3.2.1) were used for all statistical analyses.

### Role of the funding source

The funders of the study had no role in study design, data collection, data analysis, data interpretation, or writing of the report. IYM, RGW, LL, RP, and ZC had access to all data and had final responsibility for the decision to submit for publication.

## Results

From the ten study areas, 512 715 participants were enrolled (226 182 urban and 286 533 rural residents, mean age 52 years [SD 11]) and followed for incident cardiovascular disease. By Jan 1, 2017, after around 10 years of follow-up, 4781 (0·9%) had been lost and 44 037 (8·6%) had died. At baseline, 33% (69 897/210 205) of the men and 2% (6245/302 510) of the women reported drinking some alcohol in most weeks, mainly as spirits, but there was wide variation in the prevalence of drinking across study areas (appendix pp 11, 12).

For 161 498 of the participants two G→A single-nucleotide polymorphisms that alter alcohol metabolism were genotyped (rs671 and rs1229984; panel). For rs671 the overall A-allele frequency was 0·21 (range by area 0·13–0·29) and for rs1229984 it was 0·69 (0·64–0·74), with both A-alleles tending to be more common in southern than in northern study areas (appendix p 13). For each variant, the genotype frequencies within each area were consistent with Hardy-Weinberg equilibrium.

The more important variant, rs671, strongly affected drinking patterns: among men, mean alcohol intake with its AA, AG, and GG genotype was 3, 37, and 157 g per week, respectively, and the proportion of current drinkers was 1%, 16%, and 45%. The other variant, rs1229984, had a definite but smaller effect: among men, mean alcohol intake with its AA, AG, and GG genotype was 98, 106, and 157 g per week, respectively, and the proportion of current drinkers was 32%, 33%, and 43%. Among current drinkers, each variant was associated with flushing after drinking a small amount of alcohol, but the effect was much stronger with rs671 (appendix p 14).

Combinations of the rs671 genotype (AA, AG, or GG) and the rs1229984 genotype define nine possibilities for the joint rs671/rs1229984 genotype (panel). When written in alphabetic order from AA/AA to GG/GG, within each of the ten study areas these nine genotypes tended to involve progressively increasing mean male alcohol intake, with larger absolute differences between genotypes in areas where average intake was higher (figure 1). In Sichuan (the area with the highest levels of alcohol consumption) mean male alcohol intake ranged from 2 g per week for individuals with the AA/AA genotype to 443 g per week for those with the GG/GG genotype, whereas in Gansu (the area with the lowest levels of alcohol consumption) it ranged from 1 g per week to only 29 g per week (appendix p 15).

**Figure 2:**
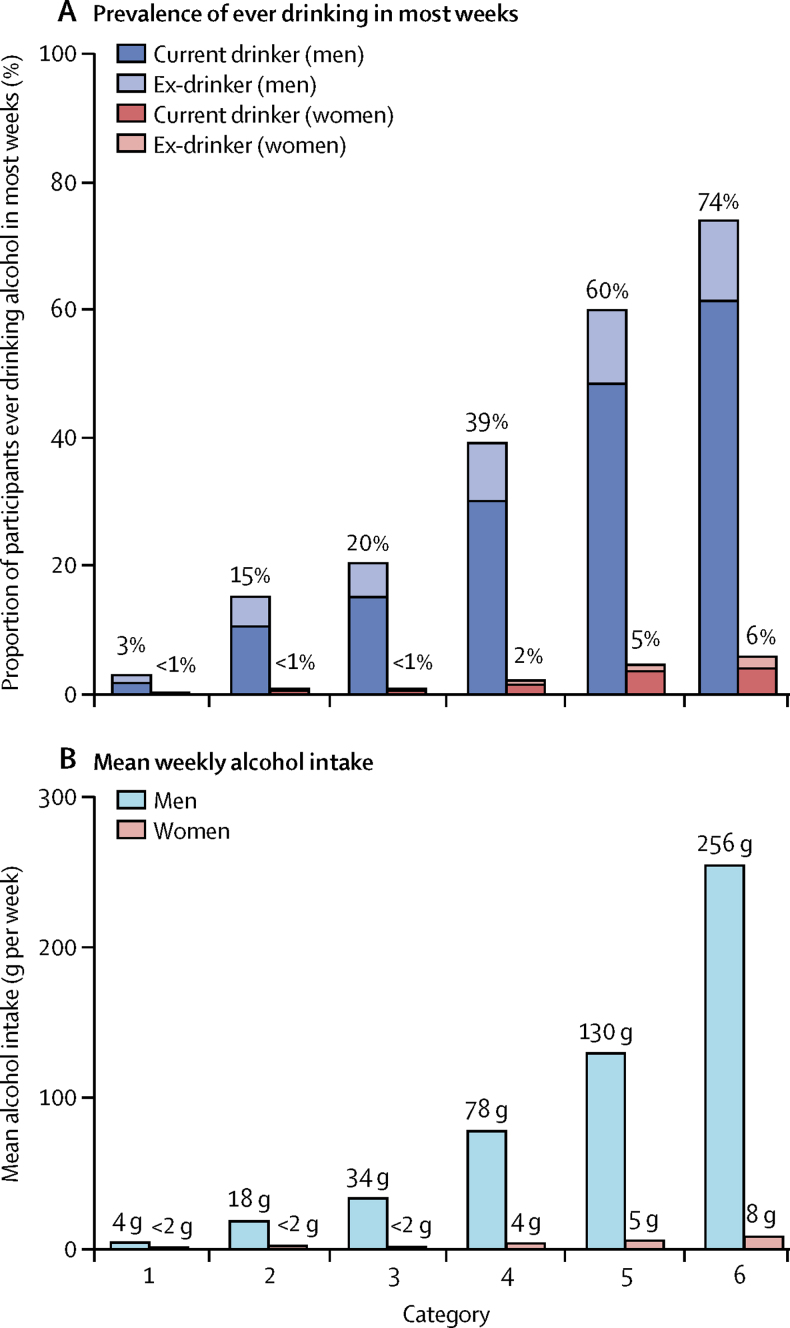
Patterns of alcohol use in six categories of genotype and study area Prevalence of ever drinking, defined as some alcohol in most weeks (A), and mean weekly alcohol intake (B) in six categories of genotype and study area.

Figure 1 shows how the 90 combinations of genotype and study area were divided into six categories, depending on the mean male alcohol intake in each combination. The prevalence of drinking was more than 20 times higher in category 6 than in category 1, and mean alcohol intake was more than 50 times higher (figure 2). Comparisons between these six categories can, as long as they are stratified by area, be used to estimate purely genotypic effects on disease rates and other factors. Apart from the large differences in drinking patterns there were no large genotype-dependent differences between these six categories in smoking or in other self-reported baseline characteristics (appendix p 19).

**Figure 3:**
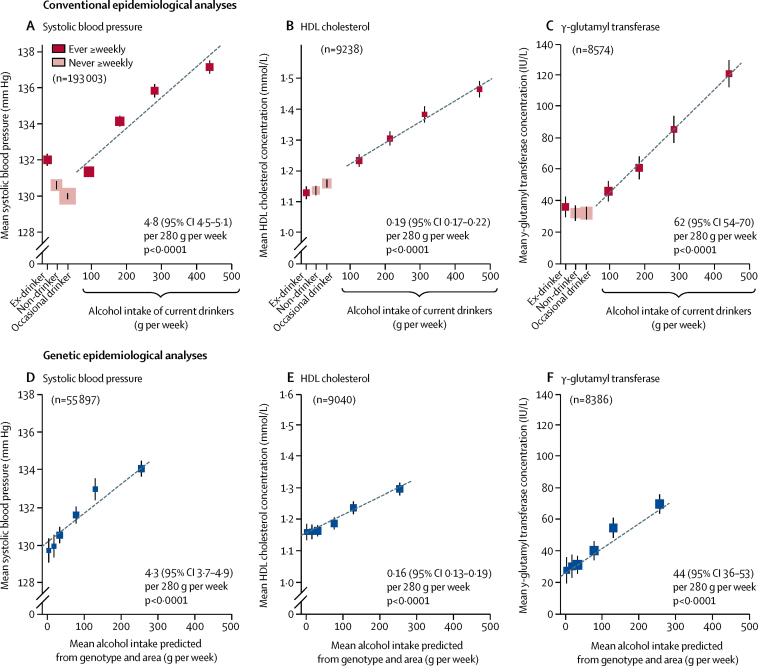
Associations of physiological factors with drinking patterns and with genotypic determinants of mean alcohol intake, in men Conventional epidemiological analyses (A–C) relate self-reported drinking patterns at baseline to mean systolic blood pressure (A), HDL cholesterol (B), and γ-glutamyl transferase (C). Results are adjusted for age, area, education, income, and smoking. The means for current drinkers are plotted against usual alcohol intake, with a fitted line giving the slope (95% CI) per 280 g alcohol per week. Genetic epidemiological analyses (D–F) ignore individual drinking patterns, and for all men relate mean alcohol intake in six categories of genotype and study area to genotypic effects on mean systolic blood pressure (D), HDL cholesterol (E), and γ-glutamyl transferase (F). Results are adjusted for age and area. The slope of the fitted line is the inverse-variance-weighted mean of the slopes of the fitted lines in each study area. The area of each square in A–F is inversely proportional to the variance of the result. Error bars show 95% CIs.

Subsequent analyses compared conventional epidemiological analyses (relating individual drinking patterns to various factors) and genetic epidemiological analyses (which ignore individual drinking patterns, and for all men relate differences between the six categories in mean alcohol intake to genotype-dependent differences between them in other factors). Among men, blood pressure and the concentrations (where available) of HDL cholesterol and γ-glutamyl transferase were positively associated with alcohol intake in both conventional and genetic epidemiological analyses (all p<0·0001; figure 3). Among current drinkers, the mean alcohol intake was about 280 g per week (appendix p 12) and systolic blood pressure increased by 4·8 mm Hg (95% CI 4·5–5·1) per 280 g per week usual alcohol intake. Similarly, systolic blood pressure increased by 4·3 mm Hg (3·7–4·9) per 280 g per week genotype-predicted mean alcohol intake. The increase in HDL cholesterol per 280 g per week alcohol intake was also similar in the conventional and in the genetic analyses. Associations with other physiological factors are shown in the appendix (p 21).

**Figure 4:**
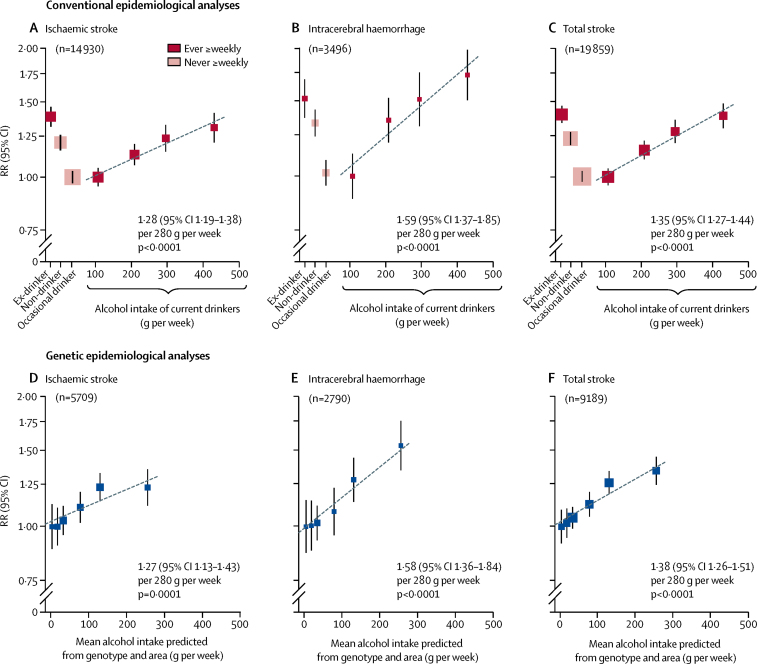
Associations of stroke incidence with drinking patterns and with genotypic determinants of alcohol intake, in men Conventional epidemiological analyses (A–C) relate self-reported drinking patterns at baseline to the incidence of ischaemic stroke (A), intracerebral haemorrhage (B), and total stroke (C). Current drinkers with the lowest mean alcohol intake are the reference group (RR=1), and results are adjusted for age, area, education, income, and smoking. The RRs for current drinkers are plotted against usual alcohol intake, with a fitted line giving the RR (95% CI) per 280 g intake per week. Genetic epidemiological analyses (D–F) ignore individual drinking patterns, and for all men relate mean alcohol intake in six categories of genotype and study area to genotypic effects on the incidence of ischaemic stroke (D), intracerebral haemorrhage (E), and total stroke (F). The category with the lowest mean alcohol intake is the reference group (RR=1), and results are adjusted for age and area. The slope of the fitted line is the inverse-variance-weighted mean of the slopes of the lines of best fit within the ten study areas. The RR is plotted on a log scale and the area of each square is inversely proportional to the variance of the log risk. The group-specific 95% CIs, calculated from this variance, are shown by error bars. RR=relative risk.

For male stroke rates, however, conventional and genetic epidemiological analyses yielded very different findings. In the conventional epidemiological analyses of stroke incidence rates among men, self-reported alcohol intake had a U-shaped association with the incidence of ischaemic stroke, intracerebral haemorrhage, and total stroke (figure 4). Moderate alcohol intake (about 100 g per week) was associated with a risk of stroke lower than that in non-drinkers or, particularly, ex-drinkers. Among current drinkers, stroke risk increased with the usual alcohol intake, with a smaller proportional increase for ischaemic stroke (RR per 280 g per week 1·28, 95% CI 1·19–1·38, p<0·0001) than for intracerebral haemorrhage (1·59, 1·37–1·85, p<0·0001; p=0·01 for the difference between these RRs). U-shaped associations with alcohol intake persisted in sensitivity analyses that excluded early follow-up, ever-smokers, and people with poor self-reported health at baseline (appendix pp 23, 24).

In the genetic epidemiological analyses, however, there were no U-shaped associations with stroke risk and there was no evidence of any protective effects of moderate alcohol intake against ischaemic stroke, intracerebral haemorrhage, or total stroke (figure 4). Stroke risk increased steadily across the whole range of genotype-predicted mean male alcohol intake (4–256 g per week), again with a smaller proportional increase for ischaemic stroke (RR per 280 g per week 1·27, 95% CI 1·13–1·43, p=0·0001) than for intracerebral haemorrhage (1·58, 1·36–1·84, p<0·0001; p=0·01 for the difference between these RRs) or total stroke (1·38, 1·26–1·51). The mean intake in all men was about 100 g per week (appendix p 12), and the corresponding RRs per 100 g per week were 1·09 (1·04–1·14), 1·18 (1·12–1·24), and 1·12 (1·09–1·16), respectively. Adjusting for potential confounders in sensitivity analyses did not materially change the results (appendix p 25).

The genotypic analyses provided no suggestion of increased stroke risk at very low levels of alcohol intake (although in the first of the six categories two-thirds were non-drinkers), or of any material deviation from log-linear relationships (figure 4). Even if, for statistical stability, the three categories with the lowest alcohol intake (categories 1–3, mean male intake around 25 g per week) were pooled and compared with categories 4 and 5 (mean male intake around 100 g per week), the genotypic analyses still indicated adverse rather than protective effects of moderate alcohol intake against ischaemic stroke and against intracerebral haemorrhage (appendix p 26). The corresponding genotypic findings within each separate study area were directionally consistent with each other, and with the overall findings (appendix p 28). For blood pressure and for stroke, the slopes of the lines in Figure 3, Figure 4 appeared to be greater for men younger than age 60 years at baseline than for other men, but the numbers were insufficient for this dependence on age to be reliable (appendix p 30).

**Figure 5:**
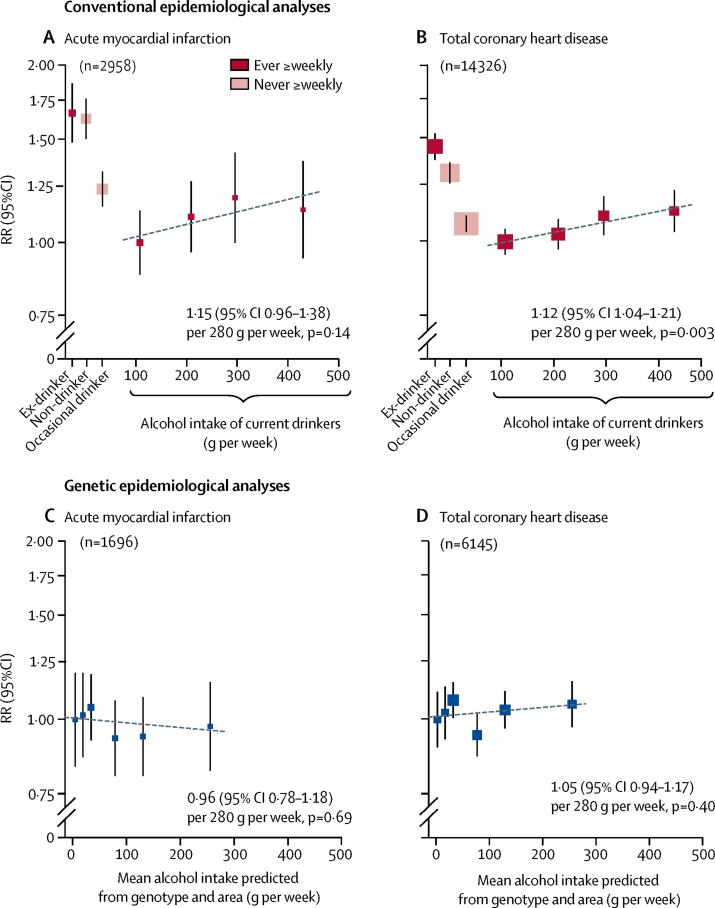
Associations of coronary heart disease incidence with drinking patterns and with genotypic determinants of alcohol intake, in men Conventional epidemiological analyses (A–B) relate self-reported drinking patterns at baseline to the incidence of acute myocardial infarction (A) and total coronary heart disease (B). Current drinkers with the lowest mean alcohol intake are the reference group (RR=1), and results are adjusted for age, area, education, income, and smoking. The RRs for current drinkers are plotted against usual alcohol intake, with a fitted line giving the RR (95% CI) per 280 g intake per week. Genetic epidemiological analyses (C–D) ignore individual drinking patterns, and for all men relate mean alcohol intake in six categories of genotype and area to genotypic effects on the incidence of acute myocardial infarction (C) and total coronary heart disease (D). The category with the lowest mean alcohol intake is the reference group (RR=1), and results are adjusted for age and area. The slope of the fitted line is the inverse-variance-weighted mean of the slopes of the lines of best fit within the ten study areas. The RR is plotted on a log scale and the area of each square is inversely proportional to the variance of the log risk. The group-specific 95% CIs, calculated from this variance, are shown by error bars. RR=relative risk.

For male coronary heart disease, conventional epidemiology and genetic epidemiology again yielded different findings (figure 5). In the conventional epidemiological analyses, there were U-shaped associations with risk. Moderate alcohol intake was associated with rates of acute myocardial infarction and of total coronary heart disease that were substantially lower than the rates in non-drinkers or ex-drinkers (and this apparently protective effect of moderate versus no alcohol intake persisted in sensitivity analyses; appendix p 32). Among current drinkers, usual alcohol intake was weakly positively associated with heart disease; the RR per 280 g per week was 1·15 (95% CI 0·96–1·38, p=0·14) for acute myocardial infarction and 1·12 (1·04–1·21, p=0·003) for total coronary heart disease.

In the genetic epidemiological analyses, however, there was no clear evidence of any net protective effect of moderate alcohol consumption against acute myocardial infarction or total coronary heart disease (figure 5). Across the whole range of genotype-predicted mean male alcohol intake, the RR per 280 g per week was 0·96 (95% CI 0·78–1·18, p=0·69) for acute myocardial infarction and 1·05 (0·94–1·17, p=0·40) for total coronary heart disease.

Among women, mean alcohol intake was low in all six categories of genotype and area (figure 2). Hence, any genotypic effects of these categories that are mediated by drinking patterns should be much smaller in women than in men, but any other (ie, pleiotropic) genotypic effects of them should be comparable in both sexes. To assess any pleiotropic effects on physiological factors or disease rates, the findings in men and in women are compared (table 2), relating the genotypic effects in both sexes to the mean alcohol intake only in men. Among women, systolic blood pressure, HDL cholesterol, γ-glutamyl transferase, ischaemic stroke, intracerebral haemorrhage, and acute myocardial infarction were not adversely associated with the genotypes that increase alcohol intake in men—indeed, blood pressure was, if anything, slightly favourably affected. After allowance for multiple testing, no material genotypic effects on other physiological factors were identified in women, except for increases of a few mm per allele in height, waist, and hip measurements (appendix p 21).

**Table 2:** Comparison between genotypic effects in men and in women

		**Systolic blood pressure (mm Hg)**		**HDL cholesterol (mmol/L)**		**γ-glutamyl transferase (IU/L)**		**Ischaemic stroke (RR)**		**Intracerebral haemorrhage (RR)**		**Acute myocardial infarction (RR)**	
Category of genotype and study area*		Men (n=55 879)	Women (n=83 017)	Men (n=9040)	Women (n=8754)	Men (n=8386)	Women (n=8088)	Men (5709 events)	Women (7087 events)	Men (2790 events)	Women (2525 events)	Men (1696 events)	Women (1246 events)
	1	129·7	129·7	1·16	1·29	28	23	1·00	1·00	1·00	1·00	1·00	1·00
	2	130·0	129·4	1·16	1·29	31	24	1·00	0·93	1·01	1·05	1·02	0·96
	3	130·5	129·2	1·17	1·28	32	25	1·03	0·98	1·02	1·16	1·05	0·99
	4	131·6	128·5	1·19	1·28	41	22	1·11	0·93	1·08	1·21	0·93	0·95
	5	133·0	128·4	1·24	1·28	55	24	1·23	0·96	1·29	1·19	0·94	1·02
	6	134·1	128·5	1·30	1·29	70	23	1·23	0·95	1·54	1·06	0·97	0·92
Effect per 280 g per week mean MALE alcohol intake (95% CI)		4·3 (3·7 to 4·9)	−0·6 (−1·0 to −0·1)	0·16 (0·13 to 0·19)	0·00 (−0·03 to 0·03)	44 (36 to 53)	0 (−3 to 3)	1·27 (1·13 to 1·43)	0·98 (0·88 to 1·09)	1·58 (1·36 to 1·84)	0·96 (0·82 to 1·12)	0·96 (0·78 to 1·18)	0·94 (0·74 to 1·20)
p value for effect greater in men than women†		<0·0001	..	<0·0001	..	<0·0001	..	0·0007	..	<0·0001	..	0·45	..

RR=relative risk.

* Six categories of genotype and study area; mean values of physiological factors and RRs of disease are adjusted for age and area, leaving only genotypic differences.

† Genotypic effect on physiological factor (slope per 280 g per week mean MALE alcohol intake) or on disease incidence (RR per 280 g per week mean MALE alcohol intake); since women consumed little alcohol, comparison between these genotypic effects in men and in women can help assess whether the genotypic effects in men are chiefly mediated by alcohol rather than by pleiotropic pathways that influence both sexes similarly.

Sensitivity analyses considered the two genetic variants separately (appendix pp 36–40). Among women, there were no significant associations of either variant with stroke or heart disease. The following results involve only men, and assess the effects of a single copy of the variant allele. For rs671, participants with the GG genotype had substantially higher mean alcohol intake, systolic blood pressure, HDL cholesterol, and γ-glutamyl transferase than did those with the AG genotype, and had a definite excess risk of stroke (GG *vs* AG: RR 1·19, 1·13–1·24, p<0·0001), though not of myocardial infarction (1·02, 0·92–1·13). For rs1229984, the effects on alcohol intake and on these three physiological factors were only about half as great, but again there was a definite excess risk of stroke (GG *vs* AG: RR 1·19, 1·11–1·27, p<0·0001), but not of myocardial infarction (1·11, 0·95–1·31).

## Discussion

Our aim is to assess causal effects of alcohol intake that should be of general relevance in any population, not just effects of particular genotypes in one east Asian population. Nevertheless, genotypic studies in China can provide generally relevant evidence about causal effects of alcohol intake. For, two genetic variants that greatly alter alcohol metabolism are common in China, the more important of which (rs671) reduces alcohol intake to almost zero in both sexes. In our population men drink more than 20 times as much as women, so these two variants have large absolute effects on alcohol intake only among men. This permits reliable comparison of the causal effects of negligible, moderate, and higher levels of mean male alcohol intake. In populations of European descent, however, only the less important of these two variants (rs1229984) is found, so genetic studies^13^, ^22^ cannot directly compare the effects of negligible and moderate alcohol intake levels.

Mainly for cultural rather than genetic reasons, alcohol intake differed substantially across our ten study areas. After grouping all possible combinations of genotype and study area into six categories according to mean male alcohol intake, there was wide variation between these six categories in mean intake, ranging from almost no alcohol to a mean of about 25 drinks per week among men. By contrast, mean alcohol intake was low among women in all six categories. Hence, the findings among women are used mainly to show that the genotypic findings among men were mediated chiefly by alcohol rather than by any other (ie, pleiotropic) effects of genotype. Although the category definitions were dependent on both genotype and study area, the genetic epidemiological analyses allowed fully for area. So, the findings from them reflect only the effects of within-area differences in genotype.

A meta-analysis of the findings from conventional epidemiological analyses of 83 prospective studies,^6^ mainly in populations of European descent, excluded non-drinkers and found that, among drinkers, stroke incidence increased steadily with the amount of alcohol consumed, whereas the incidence of myocardial infarction was slightly higher in drinkers whose usual intake was only 35 g per week rather than 100 g per week, and was approximately constant over the range of 100–350 g per week. Conventional epidemiological analyses of the present study in China are consistent with this meta-analysis,^6^ and are complemented by novel genetic epidemiological analyses that help assess causality.

Intervention studies^9^, ^10^, ^11^ have shown that alcohol increases blood pressure and HDL cholesterol. The associations between alcohol and these factors were strongly positive in our conventional epidemiological analyses among current drinkers (which relate usual alcohol intake to outcome) and were similarly strongly positive in our genetic epidemiological analyses (which relate genotype-predicted mean male alcohol intake to outcome). Both types of analysis indicated that 280 g per week of alcohol increases systolic blood pressure by about 5 mm Hg, which (in conventional analyses of blood pressure and risk in this population) is associated with increases of about 15% in ischaemic heart disease and ischaemic stroke, and of about 30% in intracerebral haemorrhage.^23^

For ischaemic stroke and, more strongly, for intracerebral haemorrhage, the genetic epidemiological comparisons across our six categories show that risk increases continuously across the whole range from negligible to high genotype-predicted mean intake, with no excess risk in the first of these categories (in which two-thirds of individuals do not drink at all and most others drink only occasionally; appendix p 17). These comparisons also show that stroke risk is about as strongly positively related to genetically predicted mean alcohol intake as it is with the usual alcohol intake among current drinkers. Neither of these two findings is what would be expected under the hypothesis that, in comparison with abstinence, moderate drinking is substantially protective (appendix pp 41, 42). For, the decrease across categories in the proportions exposed to the hypothesised excess risks of abstinence would oppose the increase across categories in the proportions exposed to the excess risks of heavier drinking.

Taken together, these two findings mean that the lower stroke risks in moderate drinkers than in non-drinkers that have been suggested by conventional epidemiological analyses of this and previous studies were not chiefly due to protective effects of moderate drinking, and may well have reflected biases of reverse causation or confounding.

If (but only if) there is no material protective effect, and the causal relationship of alcohol intake to stroke risk is approximately log-linear rather than U-shaped, then the genetic epidemiology provides a direct quantitative estimate of the strength of that causal relationship. In the genetic epidemiological analyses, the excess risks of ischaemic stroke and intracerebral haemorrhage were 27% and 58% per 280 g alcohol per week, respectively. This alcohol intake is approximately the mean amount consumed by the one-third of all men who were current drinkers. Hence, the genetic findings imply that, among all men, alcohol was responsible for about 8% of ischaemic strokes and 16% of intracerebral haemorrhages.

These excess stroke risks are about twice as great as expected just from the effects of alcohol on systolic blood pressure.^23^ This discrepancy might be because blood pressure differences due to these genetic factors persist throughout adult life, or because transient effects of alcohol on blood pressure (and hence on stroke risk) are greater at other times than at our daytime recruitment clinics, or because of adverse effects of alcohol on factors other than blood pressure.

For each of the two genetic variants, the A allele was nearly dominant in its effects on alcohol intake, physiological factors, and stroke, but although the decreases in alcohol intake and physiological factors with one A allele versus none were less than half as great for rs1229984 as for rs671, the corresponding decreases in stroke appeared surprisingly similar (appendix pp 44, 45). This apparent discrepancy may, unless it is a chance finding, be because rs1229984 reduces alcohol exposure both through reducing alcohol intake and, for a given intake, through accelerating alcohol clearance.

For heart disease, the genetic epidemiological analyses are more difficult to interpret reliably. For acute myocardial infarction, they suggest little net effect on risk over the range from near zero to about 250 g per week of predicted mean intake. This is what would be expected if moderate alcohol intake is not really protective, and if (as in the meta-analysis of previous conventional studies^6^) increased intake is not really hazardous above the range of intakes studied. The number of cases of acute myocardial infarction was, however, limited, so some real benefit or hazard cannot be excluded. Considering the two variants separately, our findings for rs1229984 (based on small numbers) are consistent with previous meta-analyses in European-origin populations,^13^ but our findings for rs671 do not support previous meta-analyses in east Asian populations, which suggested a moderate association between the alcohol-tolerant rs671 allele and decreased coronary heart disease risk.^24^, ^25^

The lack of a positive association between genotype-predicted alcohol intake and acute myocardial infarction suggests the adverse effects of alcohol intake on blood pressure could be offset by cardio-protective changes in other factors. Although HDL cholesterol increased substantially with alcohol intake, the causal relevance of different components of HDL cholesterol to coronary heart disease risk remain uncertain. A previous trial^26^ of the cholesteryl ester transfer protein inhibitor anacetrapib, which substantially increases HDL cholesterol, reported a coronary heart disease reduction that was no greater than would be expected just from the LDL-cholesterol-lowering effects of the drug, and in previous analyses^27^ of the China Kadoorie Biobank study, a loss-of-function variant of the cholesteryl ester transfer protein that substantially increases total HDL cholesterol had little net effect on the incidence of coronary heart disease.

A major limitation of all alcohol epidemiology is that exposure is uncertain. Drinking patterns are variable, and intake may be substantially underperceived or under-reported. If mean intake was really substantially higher than reported, then all our conventional and genotypic dose-response relationships are too steep, and the real relationships are substantially shallower. Conversely, our genotypic dose-response relationships would have been about 10% stronger if our calculations of mean intake had included ex-drinkers, whose previous intake was unknown, as having zero intake rather than the mean intake of other participants (appendix p 17).

Since participants reported drinking mainly spirits, the effects of other drinks (eg, red wine) could not be assessed. Although many biomarkers were measured, they might not capture some important mechanisms by which alcohol could affect myocardial infarction risk. The two enzymes affected by the genetic variants we studied are involved in many biochemical pathways, so increases or decreases in their activity could have multiple physiological effects.^28^, ^29^ In women, however, there were no material genotypic associations with cardiovascular risk or physiological factors, with the exception of small effects on anthropometry and, perhaps, blood pressure. Hence, the genotypic findings in men were probably driven mainly by alcohol exposure rather than by pleiotropic effects.

The genetic epidemiological analyses in this large study do not support the apparently protective effects against stroke of moderate drinking when compared with no drinking that are suggested by conventional epidemiological analyses. Although alcohol increases blood pressure, we identified no clear net association with acute myocardial infarction, but the number of cases was limited. The number of strokes, however, was substantial, and the genetic epidemiological analyses show that alcohol intake uniformly increases blood pressure, ischaemic stroke, and haemorrhagic stroke.
