# Supplementary material for: Conventional and genetic evidence on alcohol and vascular disease aetiology: a prospective study of 500 000 men and women in China
Source: Lancet. 2019 May 4;393(10183):1831–42. doi: 10.1016/S0140-6736(18)31772-0 (PMC6497989; doi:10.1016/S0140-6736(18)31772-0)

THE LANCET

Supplementary appendix

This appendix formed part of the original submission and has been peer reviewed. We post it as supplied by the authors.

Supplement to: Millwood IY, Walters RG, Mei XW, et al. Conventional and genetic evidence on alcohol and vascular disease aetiology: a prospective study of 500 000 men and women in China. *Lancet* 2019; published online April 4. http://dx.doi.org/10.1016/S0140-6736(18)31772-0.

Contents list, in order of appearance in the text: CLICK on any item to jump to it

Page		
**3**	**Members of the China Kadoorie Biobank collaborative group**	
**4**	**Supplementary methods**	
**7**	***Text-table 1***	Two East Asian genetic variants that alter alcohol metabolism
**8**	***Webfig 1***	Study participant flowchart
**9**	***Webtable 1***	Self-reported alcohol intake at baseline in 2004-08 and at 2008 re-survey
**10**	***Webtable 2***	Baseline characteristics of men and women by alcohol drinking status
**11**	***Webtable 3***	Beverage types consumed by current drinkers
**12**	***Webtable 4***	Alcohol drinking patterns across ten study areas, ordered North to South China (N to S)
**13**	***Webtable 5***	*ALDH2*-rs671 and *ADH1B*-rs1229984 allele frequencies in ten areas, ordered N to S
**14**	***Webtable 6***	Alcohol drinking patterns by *ALDH2*-rs671 and *ADH1B*-rs1229984 genotypes
**15**	***Webtable 7***	Mean alcohol intake and category (C1-C6) in men and women, by genotype and area
**16**	***Text-fig 1***	Mean alcohol intake in men from ten study areas in China, subdivided by nine possible genotypes of two common variants that alter alcohol metabolism
**17**	***Webtable 8***	Alcohol drinking patterns by six categories (C1-C6) of genotype and area
**18**	***Text-fig 2***	Patterns of alcohol use in six categories of genotype and study area
**19**	***Webtable 9***	Baseline characteristics by six categories (C1-C6) of genotype and area
**20**	***Text-fig 3***	Associations of physiological factors with drinking patterns and with genotypic determinants of alcohol intake in men
**21**	***Webtable 10***	Associations of physiological factors with alcohol intake
**22**	***Text-fig 4***	Associations of stroke incidence with drinking patterns and with genotypic determinants of alcohol intake in men
**23**	***Webtable 11***	Conventional epidemiologic associations of stroke incidence with alcohol intake
**25**	***Webtable 12***	Genetic epidemiologic associations of stroke incidence with mean male alcohol intake
**26**	***Webtable 13***	Genetic epidemiology – CVD incidence RR, moderate v low mean male alcohol intake
**27**	***Webfig 2***	Associations within each area of genotypic categories C1-C6 and SBP, in men
**28**	***Webfig 3***	Associations within each area of genotypic categories C1-C6 and total stroke, in men
**29**	***Webfig 4***	Age vs percent drinking and mean intake in men in categories (C1-3, C4, C5 and C6)
**30**	***Webtable 14***	Relevance of age at baseline to the associations of alcohol intake with SBP, stroke and myocardial infarction among men
**31**	***Text-fig 5***	Associations of coronary heart disease incidence with drinking patterns and with genotypic determinants of alcohol intake in men
**32**	***Webtable 15***	Conventional epidemiologic associations of CHD incidence with alcohol intake
**34**	***Webtable 16***	Genetic epidemiologic associations of CHD incidence with mean male alcohol intake
**35**	***Text-table 2***	Comparison between genotypic effects in men and in women
**36**	***Webtable 17***	Associations of *ALDH2*-rs671 and *ADH1B*-rs1229984 with physiological factors
**37**	***Webfig 5***	Associations of *ALDH2*-rs671 and *ADH1B*-rs1229984 with 3 physiological factors
**38**	***Webtable 18***	Associations of cardiovascular disease with *ALDH2*-rs671 and *ADH1B*-rs1229984
**39**	***Webfig 6***	Associations of *ALDH2*-rs671 with cardiovascular disease incidence
**40**	***Webfig 7***	Associations of *ADH1B*-rs1229984 with cardiovascular disease incidence
**41**	***Webfig 8***	Ischaemic stroke: Results from (a) conventional and (b) genetic epidemiology, and expected genetic epidemiological results if moderate drinking is (c) protective and (d) not
**42**	***Webfig 9***	Intracerebral haemorrhage: Results from (a) conventional and (b) genetic epidemiology, and expected genetic epidemiological results if moderate drinking is (c) protective and (d) not
**43**	***Webfig 10***	Myocardial infarction: Results from (a) conventional and (b) genetic epidemiology, and expected genetic epidemiological results if moderate drinking is (c) protective and (d) not
**44**	***Webfig 11***	Genotypic associations of *ALDH2*-rs671 with stroke and myocardial infarction (a) observed, (b) expected if moderate drinking is protective, and (c) expected if not
**45**	***Webfig 12***	Genotypic associations of *ADH1B*-rs1229984 with stroke and myocardial infarction (a) observed, (b) expected if moderate drinking is protective, and (c) expected if not

Members of the China Kadoorie Biobank collaborative group

International Steering Committee: Junshi Chen, Zhengming Chen (PI), Robert Clarke, Rory Collins, Yu Guo, Liming Li (PI), Jun Lv, Richard Peto, Robin Walters.

International Co-ordinating Centre, Oxford: Daniel Avery, Ruth Boxall, Derrick Bennett, Yumei Chang, Yiping Chen, Zhengming Chen, Robert Clarke, Huaidong Du, Simon Gilbert, Alex Hacker, Michael Holmes, Andri Iona, Christiana Kartsonaki, Rene Kerosi, Garry Lancaster, Kuang Lin, John McDonnell, Iona Millwood, Qunhua Nie, Richard Peto, Jayakrishnan Radhakrishnan, Paul Ryder, Sam Sansome, Dan Schmidt, Rajani Sohoni, Becky Stevens, Iain Turnbull, Robin Walters, Jenny Wang, Lin Wang, Neil Wright, Ling Yang, Xiaoming Yang.

National Co-ordinating Centre, Beijing: Zheng Bian, Ge Chen, Yu Guo, Xiao Han, Can Hou, Chao Liu, Pei Pei, Shuzhen Qu, Yunlong Tan, Canqing Yu.

10 Regional Co-ordinating Centres: Qingdao Qingdao CDC: Zengchang Pang, Ruqin Gao, Shanpeng Li, Shaojie Wang, Yongmei Liu, Ranran Du, Yajing Zang, Liang Cheng, Xiaocao Tian, Hua Zhang, Yaoming Zhai, Feng Ning, Xiaohui Sun, Feifei Li. Licang CDC: Silu Lv, Junzheng Wang, Wei Hou. **Harbin** Heilongjiang Provincial CDC: Mingyuan Zeng, Ge Jiang, Xue Zhou. Nangang CDC: Liqiu Yang, Hui He, Bo Yu, Yanjie Li, Qinai Xu, Quan Kang, Ziyan Guo. **Haikou** Hainan Provincial CDC: Dan Wang, Ximin Hu, Jinyan Chen, Yan Fu, Zhenwang Fu, Xiaohuan Wang. Meilan CDC: Min Weng, Zhendong Guo, Shukuan Wu, Yilei Li, Huimei Li, Zhifang Fu. **Suzhou** Jiangsu Provincial CDC: Ming Wu, Yonglin Zhou, Jinyi Zhou, Ran Tao, Jie Yang, Jian Su. Suzhou CDC: Fang Liu, Jun Zhang, Yihe Hu, Yan Lu, Liangcai Ma, Aiyu Tang, Shuo Zhang, Jianrong Jin, Jingchao Liu. **Liuzhou** Guangxi Provincial CDC: Zhenzhu Tang, Naying Chen, Ying Huang. Liuzhou CDC: Mingqiang Li, Jinhuai Meng, Rong Pan, Qilian Jiang, Jian Lan, Yun Liu, Liuping Wei, Liyuan Zhou, Ningyu Chen, Ping Wang, Fanwen Meng, Yulu Qin, Sisi Wang. **Sichuan** Sichuan Provincial CDC: Xianping Wu, Ningmei Zhang, Xiaofang Chen, Weiwei Zhou. Pengzhou CDC: Guojin Luo, Jianguo Li, Xiaofang Chen, Xunfu Zhong, Jiaqiu Liu, Qiang Sun. **Gansu** Gansu Provincial CDC: Pengfei Ge, Xiaolan Ren, Caixia Dong. Maiji CDC: Hui Zhang, Enke Mao, Xiaoping Wang, Tao Wang, Xi Zhang. **Henan** Henan Provincial CDC: Ding Zhang, Gang Zhou, Shixian Feng, Liang Chang, Lei Fan. Huixian CDC: Yulian Gao, Tianyou He, Huarong Sun, Pan He, Chen Hu, Xukui Zhang, Pan He, Huifang Wu. **Zhejiang** Zhejiang Provincial CDC: Min Yu, Ruying Hu, Hao Wang. Tongxiang CDC: Yijian Qian, Chunmei Wang, Kaixu Xie, Lingli Chen, Yidan Zhang, Dongxia Pan, Qijun Gu. **Hunan** Hunan Provincial CDC: Yuelong Huang, Biyun Chen, Li Yin, Huilin Liu, Zhongxi Fu, Qiaohua Xu. Liuyang CDC: Xin Xu, Hao Zhang, Huajun Long, Xianzhi Li, Libo Zhang, Zhe Qiu.

Supplementary Methods

Assessment of alcohol intake by questionnaire

Alcohol intake was self-reported by participants at the baseline survey in 2004-08 using an interviewer-administered questionnaire, as previously described.[1] Participants were asked how often they had drunk alcohol during the previous 12 months (never or almost never; occasionally; only at certain seasons; every month but less than weekly; usually at least once a week, ie, ≥weekly). Those who had not usually drunk some alcohol at least once a week in the past 12 months were asked if there was period of at least a year prior to that when they had usually drunk some alcohol at least once a week.

Those who had usually drunk some alcohol at least once a week in the past 12 months were asked further questions including: frequency of drinking (days/week); types of beverage (beer, grape wine, rice wine, weak spirits with <40% alcohol content, strong spirits with ≥40% alcohol content) and amount of alcohol drunk on a typical drinking day; and the experience of flushing or dizziness after drinking. Level of alcohol intake was calculated as grams (g) of pure alcohol per week, based on the beverage type, amount drunk and frequency, assuming the following alcohol content by volume (v/v) in China: beer 4%, grape wine 12%, rice wine 15%, weak spirits 38% and strong spirits 53%.

For this report, participants were classified into four main drinking categories: Non-drinkers (never or almost never drank alcohol in the past 12 months and had not drunk in most weeks in any past year); occasional drinkers (drank alcohol in the past 12 months only occasionally, at certain seasons, or monthly, and not in most weeks, and had not drunk alcohol in most weeks in any past year); current drinkers (drank alcohol usually at least once a week during the past 12 months); ex-drinkers (did not drink alcohol in most weeks in the past 12 months but did so in some past year(s)). Current drinkers were further grouped into those drinking <140; 140-279; 280-419; 420+ g/week (men), and <70; 70+ g/week (women). These thresholds gave reasonable numbers in each group and, in men, represent a range of moderate intake (<140) to heavy intake (420+ g/week). To calculate overall mean alcohol intake, a mean intake of 5 g/week (regardless of past drinking patterns) was assigned to those who drank sometimes but less than weekly.

Alcohol intake was re-assessed in the same way among subsets of participants at resurveys in 2008 and 2013-14. These results were used to estimate the *usual* alcohol intake in each baseline category, in order to adjust for the regression dilution bias[2] in the analyses of alcohol intake versus various traits or outcomes among all participants.

Follow-up for incident cardiovascular disease

Incident cardiovascular disease and cause-specific mortality were ascertained through linkage to electronic hospital records from the nationwide health insurance system, to established local registries of stroke and coronary heart disease, and to local death registries. By Jan 1 2017, 44 037 (8.6%) participants had died and 4781 (0.9%) were lost-to-follow-up. Median follow-up was 10.1 years (interquartile range 9.2-11.1). The main study outcomes, coded by the International Classification of Diseases, tenth revision (ICD-10), were the first fatal or hospitalised non-fatal record of ischaemic stroke (ICD-10 I63; first reported stroke was ischaemic), intracerebral haemorrhage (I61; first reported stroke was haemorrhagic), total stroke (I60, I61, I63, I64, I69.0, I69.1, I69.3, I69.4), acute myocardial infarction (I21), and total coronary heart disease (I20-I25).

Genotyping and blood biochemistry measurements

***ALDH2*-rs671** and *ADH1B*-rs1229984 were genotyped in 161,498 participants using custom Illumina Golden Gate® (92,968) or Affymetrix Axiom® arrays (94,000) at BGI (Shenzhen, China). Among 25,470 genotyped with both arrays, the concordance was, respectively, 99.97% and 99.94%. A population-based sample of 151,028 participants was randomly selected from the whole cohort for

genotyping (selected by freezer box, each box containing 96 DNA samples), and this selection was used in all genetic analyses in this report. An additional 10,470 stroke and CHD cases (7583 with stroke, 4107 with CHD, and 1220 with both) were selected for genotyping as part of nested case-control studies of stroke and CHD. The additional stroke and CHD cases were used in genetic analyses of these outcomes.

Biochemistry measurements including high-density lipoprotein cholesterol (HDL-C), low-density lipoprotein cholesterol (LDL-C; directly measured), triglycerides (TG), lipoprotein(a) (Lp(a)), C-reactive protein (CRP), fibrinogen, and gamma-glutamyl transferase (GGT) were assayed at the Wolfson Laboratory (CTSU, Oxford), using baseline plasma samples. Measurements were available for 18,256 participants selected for biochemistry assays in nested case-control studies of stroke and CHD among participants with no prior diagnosis of CHD, stroke, transient ischemic attack or cancer at baseline (including 10,434 incident first stroke cases, 1,287 incident first CHD cases and 6,535 controls), of whom 17,874 also had genotype data.

Categorisation by genotype and area, based on mean male alcohol intake

The ***ALDH2* G>A** and *ADH1B* G>A genotypes defined nine groups, running alphabetically from **AA**/AA to **GG**/GG (***ALDH2*-rs671**/*ADH1B*-rs1229984): see panel in main text. Among men, mean baseline alcohol intake was calculated for each genotype in each area (9 genotypes x 10 areas). Ex-drinkers were excluded from this calculation of mean intake as their baseline intake did not reflect their long-term intake. Based on this, these 90 groups were subdivided into six categories, C1-C6, with cut-points at 10, 25, 50, 100, and 150 g/week mean alcohol intake (Text-Figure 1). This categorisation included all men with genotype information. The cut-points were selected to include adequate numbers of cases in each category, and facilitate investigation of the causal effects of alcohol across the wide range of mean alcohol intakes in C1-C6 (Text-Figure 2). As categories C1-C3 all have mean male alcohol intake ≤35 g/week (≤0.5 drinks/day), some analyses combine these three categories. Sensitivity analyses included ex-drinkers in the calculation of mean intakes, giving them their current intake (0 or 5 g/week); this had little effect on the overall mean intakes in C1-C6.

Women were split into the same six categories based on their genotype and area as men, regardless of female alcohol intake. This allowed the effects of C1-C6 to be compared between men (where genotype was strongly associated with alcohol intake) and women (where alcohol intake was low in all categories; Text-Figure 2).

Statistical methods

General linear models were used for continuous traits (eg, SBP), and Cox regression for relative risks (RRs) of disease incidence (eg, stroke). The variance of the log risk in each group, *including* the reference group, was calculated from the variances and covariances of the log RRs in all groups *except* the reference group, and used to calculate group-specific 95% CIs.[3] Participants with prior CHD, stroke or transient cerebral ischaemia at baseline were excluded (4.5%), and analyses of blood pressure excluded those taking anti-hypertensive medication at baseline (a further 3.4%).

Conventional epidemiological analyses

General linear models related continuous traits to self-reported alcohol intake groups (non, occasional, ex, or by weekly intake). Analyses were adjusted for area (10 groups), age (10-year groups: 30-39; 40-49; 50-59; 60-69; 70-79 years), education (4 groups: no formal; primary; middle/high school; college/university), household income (4 groups: <10,000; 10,000-19,999; 20,000-34,999; 35,000+ yuan/year at baseline), and smoking (6 groups in men: never regular; occasional; ex-regular; current regular <15; current regular 15-24; current regular 25+ cigarette equivalents/day, but only 4 groups in women, as the few who were current regular smokers were not subdivided).

Cox regression related incident CVD to self-reported alcohol intake categories, with exclusions as above. Analyses were adjusted for area (10 strata), age (5-year age-at-risk strata, 35-79 years), and covariates as above. Sensitivity analyses additionally excluded: events during the first three years of follow-up; participants with any major prior disease or poor self-reported health at baseline; ever-smokers; or were conducted among the genotyped subset.

Among current drinkers, adjusted mean values or log RRs and their 95% CIs for each group (<140; 140-279; 280-419; 420+ g/week in men, and <70; 70+ g/week in women) were plotted against the usual alcohol intake in that group. Straight lines of best fit to the plotted points gave the slope (or RR) for 280 g/week usual alcohol intake. This represents an alcohol intake of about 4 drinks/day. The RR per 100 g/week is approximately the cube root of this (as log RR per 100 g/week is [100/280] times log RR per 280 g/week).

Genetic epidemiological analyses

Linear regression related continuous traits, and Cox regression related incident CVD, to categories C1-C6. Overall analyses adjusted for study area (10 groups in linear models or ten strata in Cox models) and age (10-year groups in linear models and 5-year age-at-risk [35-79 years] strata in Cox models), but in contrast with the conventional epidemiological analyses no adjustments were made for covariates (although sensitivity analyses further adjusted for education, income, and smoking with, as expected, no change in the findings). In men, adjusted mean values (or log RRs) and their 95% CIs were plotted against the mean male alcohol intake for each of the categories, as shown in Text-Figures 3-5.

As categories C1-C6 are defined by area and genotype, in order to estimate within-area genotypic effects, age-adjusted analyses were also conducted within each of the ten areas. Within each area, the slope (or RR) was calculated from a straight line of best fit through the mean trait values or log RRs and their 95% CIs and the mean male alcohol intake in the categories present in that area. Within-area effects were combined by inverse-variance-weighted meta-analysis. Results are reported for 280 g/week predicted mean male alcohol intake.

Genotypic analyses in women used the same six categories (C1-C6) of genotype and area as in men, relating continuous traits and incident CVD to mean male alcohol intake in C1-C6. This approach was not intended to assess the causal effects among women, but to refute the existence of major pleiotropic effects of the genetic factors studied in men (ie, effects of genotype not mediated by male drinking patterns).

Analyses were also conducted for individual SNPs (*ALDH2*-rs671 and *ADH1B*-rs1229984) adjusted for age and area to obtain adjusted means by genotype or adjusted for age within areas and meta-analysed across ten areas to obtain per-allele effects for continuous traits, or in age- and region-stratified Cox models to obtain genotypic RRs for disease incidence.

Analyses used SAS version 9.3 and R version 3.2.1, and CKB Data Release 14.

Supplementary references

1. Millwood IY, Li L, Smith M, et al. Alcohol consumption in 0.5 million people from 10 diverse regions of China: prevalence, patterns and socio-demographic and health-related correlates. *Int J Epidemiol* 2013; **42**(3): 816-27.
2. MacMahon S, Peto R, Cutler J, et al. Blood pressure, stroke, and coronary heart disease. Part 1, Prolonged differences in blood pressure: prospective observational studies corrected for the regression dilution bias. *Lancet* 1990; **335**(8692): 765-74.
3. Plummer M. Improved estimates of floating absolute risk. *Statistics in Medicine* 2004; **23**(1): 93-104.

Text-Table 1: Two East Asian genetic variants that alter alcohol metabolism

	***ALDH2* gene**	***ADH1B* gene**
Enzyme*	ALDH2, an aldehyde dehydrogenase	ADH1, an alcohol dehydrogenase
Enzyme function	Acetaldehyde breakdown, by oxidation to acetate	Alcohol breakdown, by oxidation to acetaldehyde
Description of variants		
SNP identifier	rs671	rs1229984
Nucleotide change	G→A	G→A
Amino acid change†	Glu504→Lys	Arg48→His
Enzyme activity change	Decreased substantially	Increased substantially
Alcohol clearance rate	Unaffected	Accelerated
Acetaldehyde clearance rate	Decreased substantially	Unaffected
Alcohol intake	Reduced substantially	Reduced‡

ALDH=aldehyde dehydrogenase. ADH=alcohol dehydrogenase.

* ALDH2 is a tetramer of the ALDH2 gene product that requires all four parts to be functional, so a loss-of-function variant is nearly dominant. ADH1 is a dimer that requires two functional parts from the products of any of three similar genes, *ADH1A*, *ADH1B*, and *ADH1C*.

†For ALDH2 and for ADH1B, the effects on the enzyme of these amino acid changes are described in some other reports as altering the *1 into the *2 enzyme isoform.

‡The *ADH1B*-rs1229984 East Asian variant is nearly dominant, with AA and AG having similar effects on alcohol intake.

Webfigure 1: Study participant flowchart

a) Participants in analyses of risk factors and disease incidence

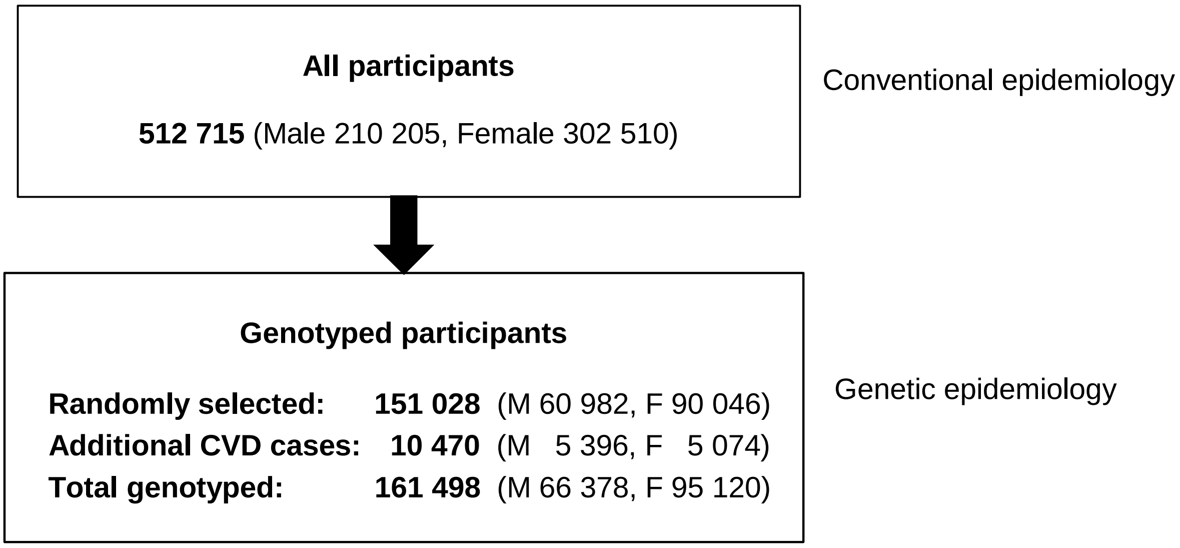

b) Participants in analyses of biochemistry measurements

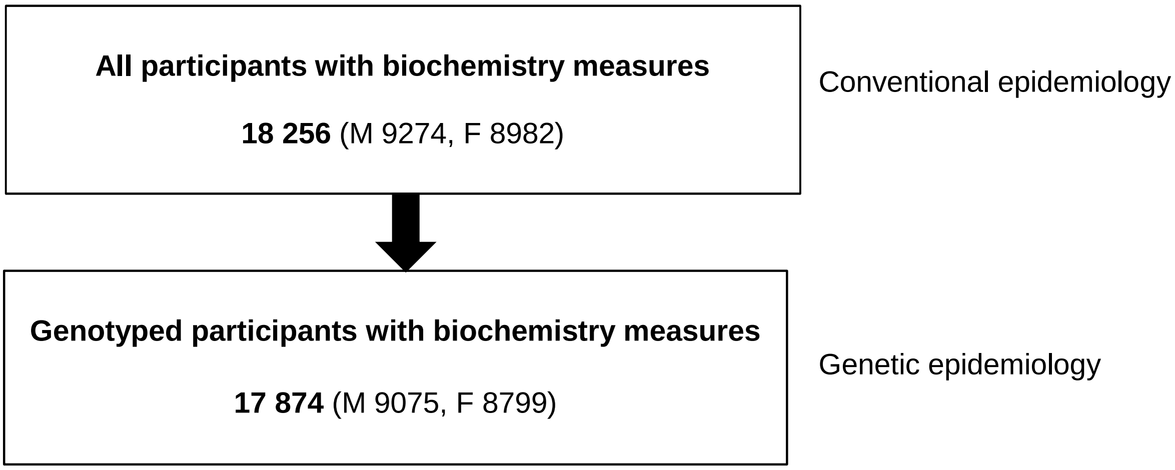

Webtable 1: Self-reported alcohol intake at baseline and at re-survey, by baseline alcohol grouping

~5% of randomly-selected survivors attended re-surveys in 2008 and 2013-14, yielding an estimate of the *usual* alcohol intake for each baseline alcohol grouping

	Results at baseline			Results at re-survey, by *baseline* alcohol grouping				
	Baseline survey, 2004-08			Re-survey in 2008		Re-survey in 2013-14		Average of 2 re-surveys
	N	%	Mean intake (g/week)[1]	N	Mean intake (g/week)[1,2]	N	Mean intake (g/week)[1]	*Usual intake* (g/week)[3]
**a) Men, by baseline alcohol grouping**								
Ex-drinker	18 294	8.7	2.8	652	37.8	767	67.0	52.4
Non-drinker (never ≥weekly)	42 780	20.4	0.0	1548	3.6	1845	4.2	3.9
Occasional drinker (never ≥weekly)	79 234	37.7	5.0	2960	21.0	3659	33.6	27.3
Current drinker, <140 g/week	25 092	11.9	79.5	987	97.1	1199	119.4	108.3
140-279 g/week	18 905	9.0	222.6	659	185.0	888	233.1	209.1
280-419 g/week	12 832	6.1	370.2	463	283.9	589	306.8	295.4
≥420 g/week	13 068	6.2	689.7	503	440.8	632	418.0	429.4
All men	*210 205*	*100.0*	*97.1*	*7772*	*85.4*	*9579*	*102.0*	*93.7*
**b) Women, by baseline alcohol grouping**								
Ex-drinker	2658	0.9	2.5	113	13.4	151	11.8	12.6
Non-drinker (never ≥weekly)	192 323	63.6	0.0	7602	0.8	9988	0.7	0.8
Occasional drinker (never ≥weekly)	101 284	33.5	5.0	4036	4.6	4974	3.8	4.2
Current drinker, <70 g/week	3224	1.1	34.0	124	27.9	162	21.1	24.5
≥70 g/week	3021	1.0	202.7	139	145.0	187	101.4	123.2
All women	*302 510*	*100.0*	*4.1*	*12 014*	*4.2*	*15 462*	*3.2*	*3.7*

[1] Calculations assign an intake of 5 g/week (regardless of past drinking patterns) to those who now drink sometimes but less than weekly.

[2] The 2008 re-survey values are taken as the *usual* alcohol intake for cross-sectional analyses (among current drinkers) of baseline traits.

[3] This calculation (of the average of 2 re-surveys) is taken as the *usual* alcohol intake for prospective analyses (among current drinkers) of events occurring during the ten year follow-up period.

Webtable 2: Baseline characteristics of men and women by alcohol drinking status

	Men					Women				
	Ex-drinker	Non-drinker	Occasional drinker	Current drinker	All men	Ex-drinker	Non-drinker	Occasional drinker	Current drinker	All women
**N**	18 294	42 780	79 234	69 897	210 205	2658	192 323	101 284	6245	302 510
**Region, %**										
Rural	57.8	67.3	56.3	49.9	56.5	73.3	56.8	52.3	54.8	55.4
Urban	42.2	32.7	43.7	50.1	43.5	26.7	43.2	47.7	45.2	44.6
**Age group, %**										
30-39 years	6.4	9.6	18.2	14.1	14.1	4.5	14.9	18.3	10.3	15.9
40-49 years	18.6	21.1	30.4	32.4	28.2	19.5	28.5	35.9	30.5	30.9
50-59 years	33.2	29.8	28.8	31.7	30.3	39.0	31.8	29.3	32.8	31.0
60-69 years	29.8	27.0	16.3	16.3	19.7	28.3	18.5	12.8	19.6	16.7
70-79 years	12.0	12.5	6.3	5.5	7.8	8.7	6.4	3.7	6.7	5.5
**Mean age (SD), years**	57.1 (10.3)	56.1 (11.1)	51.2 (10.8)	51.6 (10.2)	52.8 (10.9)	56.7 (9.4)	52.2 (10.7)	49.7 (9.9)	53.2 (10.3)	51.5 (10.5)
**Education, %**										
No formal education	10.0	15.3	6.2	7.6	8.9	28.3	31.8	13.1	21.2	25.3
Primary school	42.3	40.8	28.4	32.1	33.4	41.6	33.4	27.5	31.3	31.4
Middle school	30.0	26.6	35.1	34.2	32.4	17.7	22.9	30.5	22.4	25.4
High school	13.6	12.5	20.5	18.1	17.5	9.1	9.7	20.6	17.1	13.5
College/ university	6.2	4.7	9.8	8.0	7.9	3.2	2.3	8.2	8.1	4.4
**Household income, %**										
<10,000 yuan/year	27.7	31.8	27.2	20.7	26.0	44.0	29.8	29.0	34.2	29.8
10,000-19,999	28.9	26.5	29.6	27.8	28.3	26.8	27.4	33.9	26.5	29.6
20,000-34,999	24.3	23.8	24.2	28.0	25.4	16.7	25.2	22.7	21.5	24.2
35,000+ yuan/year	19.0	17.8	18.9	23.5	20.2	12.5	17.6	14.4	17.9	16.5
**Mean physical activity (SD), MET-hours/day**	18.0 (14.5)	20.7 (15.1)	22.9 (15.6)	22.9 (15.0)	22.0 (15.3)	18.3 (11.1)	21.0 (13.3)	19.4 (11.7)	20.0 (11.6)	20.4 (12.8)
**Smoking status, %**										
Never-regular smoker	8.4	24.0	16.3	8.0	14.4	67.4	96.9	93.4	71.2	94.9
Occasional smoker	7.9	8.8	16.1	8.1	11.2	10.1	0.9	3.0	9.7	1.8
Ex-regular smoker	26.1	13.3	10.9	12.7	13.3	11.2	0.6	0.9	3.4	0.9
Current regular smoker	57.6	53.9	56.7	71.3	61.1	11.3	1.6	2.7	15.7	2.4
**Medical history, %**										
Coronary heart disease	6.3	3.5	2.2	1.9	2.7	4.2	3.3	3.1	2.7	3.2
Stroke or TIA	6.9	3.3	1.7	1.3	2.3	2.6	1.4	1.2	0.9	1.3
Diabetes	6.5	3.7	2.4	2.0	2.9	5.4	3.9	2.3	1.3	3.3
Cancer	1.3	0.7	0.3	0.2	0.5	1.1	0.5	0.5	0.4	0.5
Any chronic disease[1]	40.5	26.9	19.9	18.2	22.6	36.9	23.0	20.1	22.2	22.1
Anti-hypertensive use	11.9	7.1	3.7	3.2	4.9	7.8	5.3	3.7	2.8	4.7
Statin use	0.6	0.3	0.1	0.2	0.2	0.7	0.3	0.2	0.2	0.2
Poor self-rated health	18.9	11.3	7.6	6.3	8.9	24.4	11.5	10.9	10.2	11.4

[1]Coronary heart disease, stroke, transient ischaemic attack (TIA), diabetes, cancer, emphysema, bronchitis, cirrhosis, hepatitis, peptic ulcer, tuberculosis, gallstone/gallbladder disease, kidney disease, rheumatoid arthritis

Webtable 3: Main beverage types consumed by current drinkers

	Men (69 897 current drinkers / 210 205 participants)		Women (6245 current drinkers / 302 510 participants)	
	% of all current drinkers	Mean intake per current drinker (g/week)	% of all current drinkers	Mean intake per current drinker (g/week)
Strong spirit drinkers	46.8	351.6	49.1	166.0
Weak spirit drinkers	22.8	299.3	12.7	96.8
Rice wine drinkers	11.3	211.2	6.2	66.7
Grape wine drinkers	0.9	65.9	9.8	29.5
Beer drinkers	18.2	156.1	22.2	66.6

Webtable 4: Alcohol drinking patterns across ten study areas, ordered from North to South China

	N	Ex-drinker (%)	Non-drinker (%)	Occasional drinker (%)	Current drinker (%)	Mean intake in current drinkers (g/week)	Mean intake overall (g/week)[1]
**a) Men**							
Harbin	23 252	8.4	8.3	33.8	49.5	194.7	98.3
Qingdao	15 624	5.5	10.9	35.6	48.0	271.5	132.3
Henan	27 841	6.8	8.4	59.4	25.5	246.7	66.1
Gansu	19 298	2.7	40.2	49.5	7.6	204.9	18.1
Suzhou	22 363	11.2	16.7	31.4	40.7	295.5	122.2
Sichuan	21 315	14.8	10.9	23.9	50.4	422.7	214.6
Zhejiang	24 027	7.2	31.5	23.1	38.2	355.0	136.8
Hunan	26 370	12.5	33.4	29.9	24.3	277.6	69.1
Liuzhou	19 321	10.1	15.7	47.2	27.0	195.2	55.4
Haikou	10 794	4.1	33.2	47.0	15.8	211.4	35.8
**All areas**	**210 205**	**8.7**	**20.4**	**37.7**	**33.3**	**285.7**	**97.1**
**b) Women**							
Harbin	34 304	0.6	37.2	57.7	4.5	72.7	6.2
Qingdao	19 884	0.2	54.7	43.6	1.5	89.3	3.5
Henan	35 515	0.3	26.5	72.3	1.0	66.8	4.3
Gansu	30 589	0.1	81.3	18.5	0.1	60.2	1.0
Suzhou	30 896	0.4	89.2	9.8	0.6	79.4	1.0
Sichuan	34 371	3.8	48.8	41.2	6.2	187.2	13.8
Zhejiang	33 677	0.5	88.5	9.5	1.5	108.7	2.1
Hunan	33 530	1.1	84.9	12.9	1.2	109.5	1.9
Liuzhou	30 852	1.1	48	48.6	2.3	53.4	3.7
Haikou	18 892	0.1	89.8	9.7	0.4	79.4	0.8
**All areas**	**302 510**	**0.9**	**63.6**	**33.5**	**2.1**	**115.6**	**4.1**

[1] Calculations assign an intake of 5 g/week (regardless of past drinking patterns) to those who now drink sometimes but less than weekly

Webtable 5: ALDH2-rs671 and *ADH1B*-rs1229984 allele frequencies across the ten study areas, ordered from North to South China

	**No. of men and women genotyped**	***ALDH2*-rs671 A-allele frequency[1,2]**	***ADH1B*-rs1229984 A-allele frequency[1,2]**
Harbin	17 836	0.16	0.67
Qingdao	11 763	0.18	0.69
Henan	17 762	0.13	0.66
Gansu	16 096	0.14	0.64
Suzhou	15 163	0.23	0.71
Sichuan	16 401	0.19	0.68
Zhejiang	18 022	0.28	0.72
Hunan	16 331	0.27	0.73
Liuzhou	13 998	0.24	0.70
Haikou	7656	0.29	0.74
**All areas**	**151 028**	**0.21**	**0.69**

[1] A-alleles decrease alcohol tolerability. Genotype distributions did not deviate from Hardy-Weinberg equilibrium within areas.

[2] Corresponding frequencies in European-origin populations (1KGP) are 0.00 (*ALDH2*-rs671) and 0.03 (*ADH1B*-rs1229984).

Webtable 6: Alcohol drinking patterns overall, by *ALDH2*-rs671 genotype, and by *ADH1B*-rs1229984 genotype

Characteristic	Overall in CKB study	Genotyped subset (rest of the table)	*ALDH2*-rs671			P-value for trend / allele[1]	*ADH1B*-rs1229984			P-value for trend / allele[1]
			AA	AG	GG		AA	AG	GG	
**a) Men**										
N	210 205	60 982	2770	19 880	38 332		29 155	26 083	5744	
Ex-drinker, %	8.7	8.6	1.2	5.7	10.4	<0.0001	8.6	8.0	8.6	0.12
Non-drinker, %	20.4	20.2	71.2	31.5	10.5	<0.0001	21.5	21.1	16.6	<0.0001
Occasional drinker, %	37.7	37.2	26.3	46.4	33.8	<0.0001	38.2	37.7	32.2	<0.0001
Current drinker, %	33.3	34.0	1.2	16.3	45.2	<0.0001	31.7	33.2	42.5	<0.0001
Flushing response[2] in current drinkers, %	17.9	18.4	53.6	53.1	10.3	<0.0001	18.0	16.6	12.7	<0.0001
Mean alcohol intake in current drinkers, g/week	285.7	286.3	88.2	192.0	281.6	<0.0001	254.3	267.5	315.1	<0.0001
Mean alcohol intake overall, g/week[3]	106.1	108.4	2.5	37.1	157.0	<0.0001	98.0	105.7	156.9	<0.0001
**b) Women**										
N	302 510	90 046	4106	28 951	56 989		42 904	38 217	8925	
Ex-drinker, %	0.9	0.9	0.1	0.4	1.1	<0.0001	0.7	0.8	0.9	0.24
Non-drinker, %	63.6	63.9	88.0	71.8	60.8	<0.0001	66.2	65.1	62.4	<0.0001
Occasional drinker, %	33.5	33.2	11.6	27.1	35.5	<0.0001	31.3	32.1	33.9	<0.0001
Current drinker, %	2.1	2.1	0.2	0.7	2.6	<0.0001	1.7	1.9	2.9	<0.0001
Flushing response[2] in current drinkers, %	23.6	22.8	-	54.3	14.5	<0.0001	24.4	21.4	14.1	0.05
Mean alcohol intake in current drinkers, g/week	115.6	115.2	-	65.9	94.6	<0.0001	81.8	92.3	106.7	0.06
Mean alcohol intake overall, g/week[3]	4.1	4.1	0.6	1.9	5.1	<0.0001	3.5	3.9	5.6	<0.0001

[1] Prevalences or means are adjusted for age within areas (which had little effect) and combined across areas by taking inverse-variance-weighted averages.

[2] Flushing after drinking a small amount of alcohol intake.

[3] Calculations assign an intake of 5 g/week to occasional drinkers, and excludes ex-drinkers

Webtable 7: Mean alcohol intake and category (C1-C6) in men and women, by genotype and study area

	**ALDH2-rs671**/ADH1B-rs1229984 genotype (see Panel in main text) Mean alcohol intake[1], and category (see Text-Figure 1)																	
	**AA**/AA		**AA**/AG		**AA**/GG		**AG**/AA		**AG**/AG		**AG**/GG		**GG**/AA		**GG**/AG		**GG**/GG	
**a) Men**																		
Gansu	1.0	C1	0.4	C1	0.3	C1	5.4	C1	9.4	C1	9.9	C1	21.0	C2	27.3	C3	29.1	C3
Haikou	1.3	C1	1.1	C1	1.2	C1	11.3	C2	16.3	C2	36.7	C3	67.9	C4	67.1	C4	121.9	C5
Liuzhou	1.9	C1	2.1	C1	2.5	C1	18.0	C2	23.0	C2	68.4	C4	87.5	C4	90.6	C4	116.1	C5
Henan	2.4	C1	2.3	C1	1.9	C1	14.7	C2	18.1	C2	52.0	C4	82.7	C4	87.7	C4	138.7	C5
Harbin	3.9	C1	2.6	C1	5.4	C1	32.4	C3	42.1	C3	82.4	C4	134.7	C5	138.8	C5	161.6	C6
Qingdao	7.8	C1	8.2	C1	25.6	C3	51.4	C4	62.0	C4	126.7	C5	168.9	C6	184.7	C6	222.5	C6
Hunan	0.7	C1	0.6	C1	1.5	C1	30.2	C3	29.7	C3	91.0	C4	113.7	C5	131.4	C5	236.5	C6
Suzhou	0.9	C1	1.8	C1	15.1	C2	39.9	C3	46.3	C3	97.7	C4	212.2	C6	221.6	C6	311.0	C6
Zhejiang	0.7	C1	2.1	C1	2.6	C1	25.9	C3	42.4	C3	77.7	C4	247.7	C6	248.2	C6	352.8	C6
Sichuan	1.5	C1	1.7	C1	1.9	C1	53.3	C4	78.7	C4	207.9	C6	340.3	C6	368.5	C6	443.2	C6
**b) Women**																		
Gansu	0.3	C1	0.0	C1	0.5	C1	0.7	C1	0.9	C1	1.4	C1	1.1	C2	1.2	C3	1.5	C3
Haikou	0.2	C1	0.2	C1	0.0	C1	0.4	C2	0.4	C2	0.5	C3	0.8	C4	0.9	C4	1.1	C5
Liuzhou	1.3	C1	1.1	C1	0.8	C1	2.7	C2	2.6	C2	2.4	C4	3.9	C4	4.4	C4	6.1	C5
Henan	2.0	C1	1.9	C1	2.6	C1	3.2	C2	3.4	C2	4.6	C4	4.4	C4	4.9	C4	4.6	C5
Harbin	0.8	C1	1.9	C1	0.1	C1	2.7	C3	3.0	C3	5.9	C4	7.6	C5	7.1	C5	9.0	C6
Qingdao	0.6	C1	0.7	C1	0.6	C3	1.7	C4	2.4	C4	2.2	C5	4.2	C6	4.2	C6	4.9	C6
Hunan	0.1	C1	0.1	C1	1.4	C1	0.5	C3	1.0	C3	1.1	C4	2.6	C5	3.5	C5	4.3	C6
Suzhou	0.1	C1	0.3	C1	0.1	C2	0.5	C3	0.7	C3	0.4	C4	0.9	C6	1.4	C6	1.0	C6
Zhejiang	0.1	C1	0.1	C1	0.2	C1	0.8	C3	0.7	C3	1.6	C4	2.7	C6	3.8	C6	5.0	C6
Sichuan	0.8	C1	0.4	C1	0.7	C1	2.5	C4	4.4	C4	7.5	C6	17.7	C6	19.1	C6	36.0	C6

[1] Calculations assign an intake of 5 g/week to occasional drinkers, ex-drinkers are excluded from the calculation.

Text-Figure 1: Mean alcohol intake in men from ten study areas in China, subdivided by nine possible genotypes of two common variants that alter alcohol metabolism

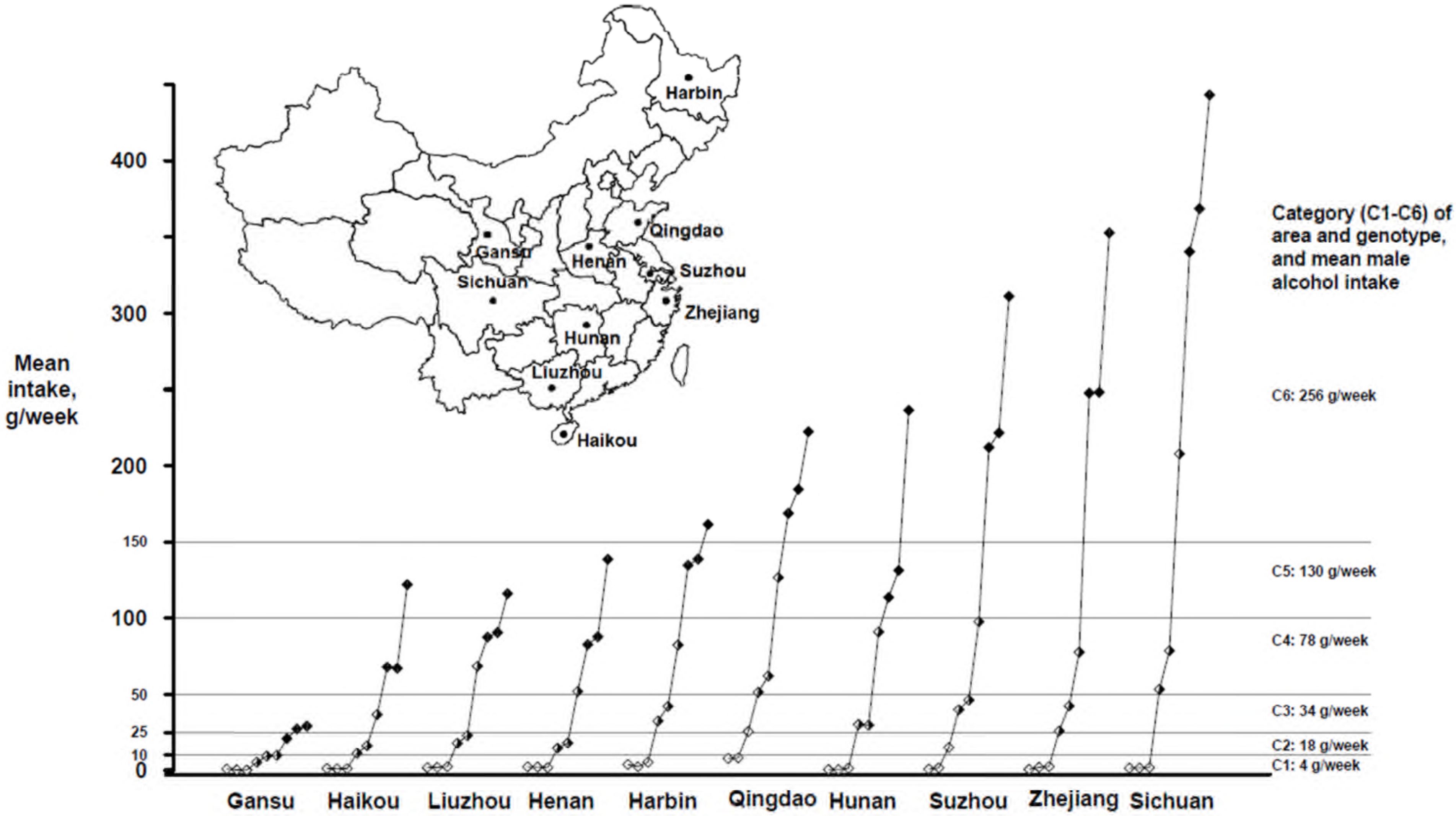

For each genotype, the A allele discourages alcohol consumption. Within each area, mean alcohol intake was plotted according to the nine possible *ALDH2*-rs671 and *ADH1B*-rs1229984 genotypes (each AA, AG, or GG) from AA/AA homozygosity for both variants to GG/GG homozygosity for both variants. White, half white/half black and black symbols denote respectively the AA, AG and GG genotypes of rs671. Alcohol intake thresholds were defined at 10, 25, 50, 100, and 150 g per week to assign individuals into six categories of mean male alcohol intake, based on their genotype and area.

Webtable 8: Alcohol drinking patterns by six categories (C1-C6) of genotype and area (see Text-Figure 1)

	Category of genotype and area						
	**C1**	**C2**	**C3**	***C1-C3***	**C4**	**C5**	**C6**
**a) Men (60 982 genotyped)**							
N	4269	6352	11 974	*22 595*	13 528	9046	15 813
Ex-drinker, %	1.2	4.5	5.3	*4.3*	9.0	11.5	12.6
Non-drinker, %	68.5	26.4	37.4	*40.2*	12.4	8.2	5.3
Occasional drinker, %	28.6	58.5	42.2	*44.2*	48.4	31.9	20.7
Current drinker overall, %	1.7	10.6	15.0	*11.3*	30.2	48.5	61.4
<140 g/week, %	1.0	7.1	8.3	*6.6*	14.0	21.5	13.7
140-279 g/week, %	0.5	2.2	3.8	*2.7*	7.3	15.2	16.2
280+ g/week, %	0.2	1.3	3.0	*2.0*	8.8	11.8	31.5
Mean alcohol intake in current drinkers, g/week	147.2	137.5	197.0	*179.9*	228.2	234.6	362.0
Flushing response in current drinkers, %	56.2	40.2	49.1	*47.0*	19.8	11.3	13.5
Mean alcohol intake overall, g/week							
All except ex-drinkers[1]	4.0	18.3	33.5	*23.5*	78.3	130.2	255.5
All men[2]	4.0	17.6	31.8	*22.6*	71.5	115.6	223.7
**b) Women (90 046 genotyped)**							
N	6439	9723	17 173	*33 335*	19 940	13 051	23 720
Ex-drinker, %	0.1	0.3	0.3	*0.2*	0.6	1.0	1.9
Non-drinker, %	88.5	65.3	82.1	*78.5*	49.7	50.0	63.1
Occasional drinker, %	11.3	33.9	17.1	*20.9*	48.2	45.4	31.1
Current drinker overall, %	0.1	0.5	0.5	*0.5*	1.5	3.6	4.0
<70 g/week, %	0.1	0.5	0.4	0.4	1.0	2.2	1.4
70+ g/week, %	0.0	0.1	0.2	0.1	0.5	1.4	2.6
Mean alcohol intake in current drinkers, g/week	50.4	33.8	59.6	*50.2*	72.8	86.4	152.8
Flushing response in current drinkers, %	37.5	44.2	55.0	*50.3*	25.3	10.5	23.7
Mean alcohol intake overall, g/week							
All except ex-drinkers[1]	0.6	1.9	1.2	*1.3*	3.5	5.4	7.8
All women[2]	0.6	1.9	1.2	*1.3*	3.5	5.4	7.7

[1] Calculations assign an intake of 5 g/week to occasional drinkers, and excludes ex-drinkers (which effectively assigns them the mean intake of all other participants in their category of genotype and area).

[2] Calculations assign an intake of 5 g/week (regardless of past drinking patterns) to those who now drink sometimes but less than weekly.

Text-Figure 2: Patterns of alcohol use in six categories of genotype and study area

Each individual's category (C1-C6) depends only on genotype and study area, not on sex or actual drinking patterns (even though the category definitions were made by calculating the mean male alcohol intake in the 90 combinations of genotype and area, then using cut-points of 10, 25, 50, 100 and 150 g/week)

a) Percent drinking alcohol

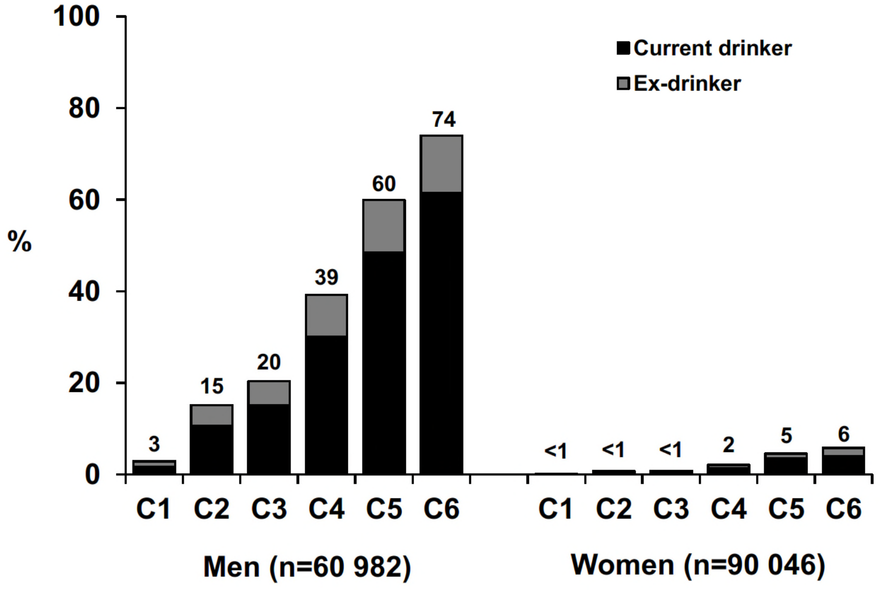

b) Mean alcohol intake, g/week

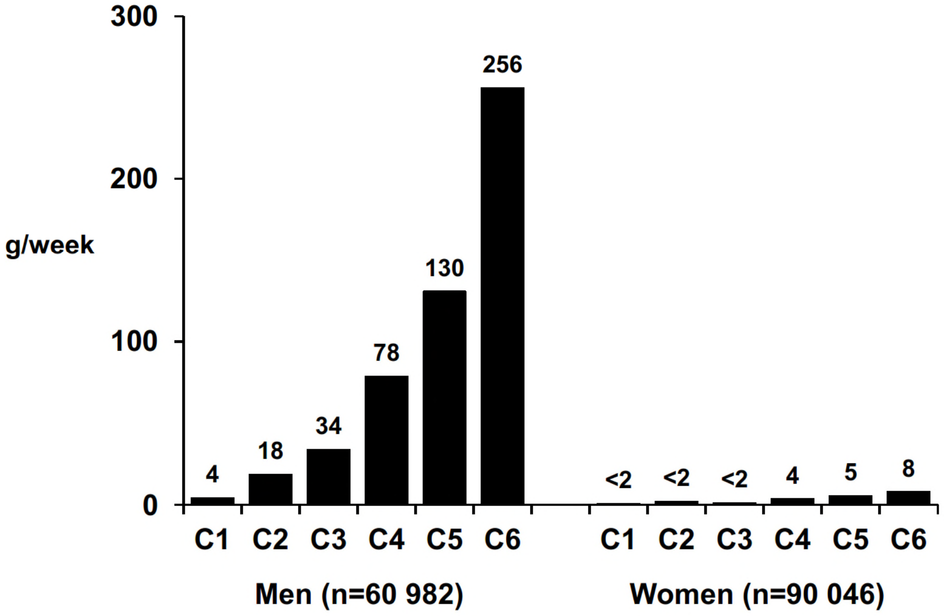

Webtable 9: Baseline characteristics by six categories (C1-C6) of genotype and area

	All participants	Genotyped subset	Category of genotype and area[1]						P-value for trend by mean male intake[2]
			C1	C2	C3	C4	C5	C6	
**a) Men**									
N	210 205	60 982	4269	6352	11 974	13 528	9046	15 813	
Mean age, years	52.8	52.9	53.1	52.9	53.1	53.0	52.6	52.8	0.04
Education > 6 years, %	25.3	25.2	23.0	25.3	25.3	25.0	26.6	24.7	0.05
Income >20,000 yuan[3], %	45.6	44.7	43.6	45.8	44.8	45.3	43.8	44.6	0.56
Ever regular smoker, %	74.4	74.4	72.6	74.2	74.7	74.5	74.7	74.5	0.67
Mean physical activity, MET-hours/day[4]	22.0	22.1	21.9	21.8	22.0	22.1	22.2	22.2	0.30
**b) Women**									
N	302 510	90 046	6439	9723	17 173	19 940	13 051	23 720	
Mean age, years	51.5	51.5	51.7	51.5	51.5	51.4	51.5	51.4	0.48
Education > 6 years, %	17.9	17.8	18.1	17.7	17.9	17.6	18	17.5	0.15
Income >20,000 yuan[3], %	40.7	39.5	38.7	39.2	39.4	39.8	39.2	39.6	0.30
Ever regular smoker, %	3.2	3.2	3.2	2.9	3.2	3.0	3.2	3.6	0.03
Mean physical activity, MET-hours/day[4]	20.4	20.5	20.4	20.6	20.4	20.5	20.3	20.6	0.18

[1] Prevalences or means are adjusted for area and (where appropriate) age.

[2] The P-value for trend is from an inverse-variance-weighted meta-analysis across ten areas, adjusted (where appropriate) for age.

[3] At the midpoint of the baseline survey of household income in 2004-8, 1 yuan=0.13 USD=0.07 GBP

[4] MET: metabolic equivalent (1 MET is 1 kcal/kg/hour, and is approximately the energy cost of sitting quietly)

Text-Figure 3: Associations of physiological factors with drinking patterns and with genotypic determinants of alcohol intake in men

I. Conventional epidemiology

Mean level, adjusted for area, age and covariates
by self-reported alcohol intake
Black: ever ≥weekly, white: never ≥weekly

II. Genetic epidemiology

Mean level, adjusted for area and age but no covariates
by genotype-predicted mean alcohol intake (in 6 categories)
Mendelian randomisation

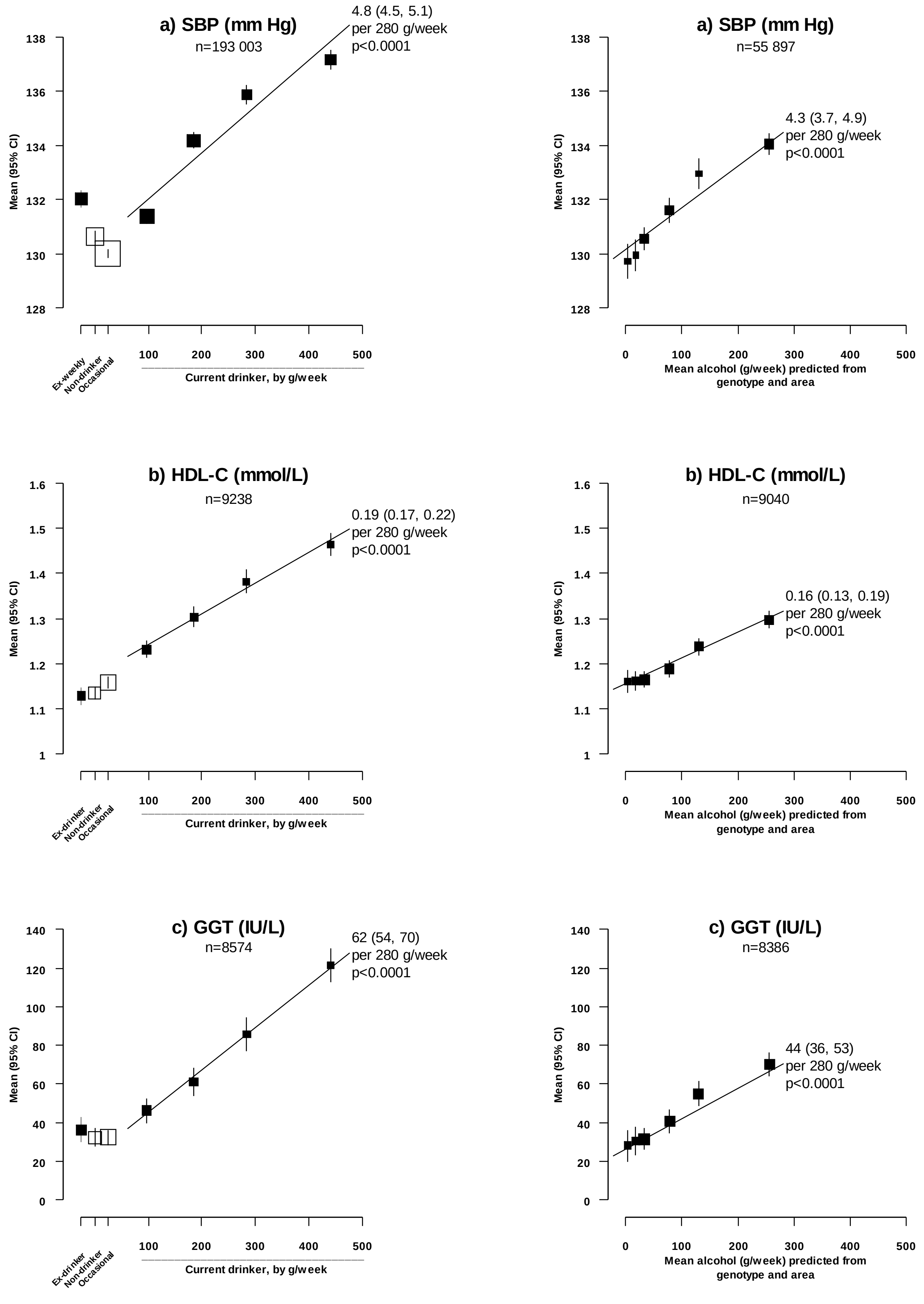

Webtable 10: Associations of physiological factors with alcohol intake

	Conventional epidemiology[1]			Genetic epidemiology[2]		
	N	Slope (95% CI) per 280 g/week usual alcohol intake in current drinkers, and P-value		N	Slope (95% CI) per 280 g/week genotype-predicted mean male alcohol intake, and P-value	
**a) Men**						
SBP, mmHg	66071	4.79 (4.47, 5.11)	<0.0001	55897	4.29 (3.72, 4.85)	<0.0001
DBP, mmHg	66071	2.87 (2.68, 3.06)	<0.0001	55897	3.07 (2.75, 3.40)	<0.0001
Heart rate, bpm	67779	2.65 (2.44, 2.85)	<0.0001	57965	1.94 (1.58, 2.30)	<0.0001
BMI, kg/m^2	67778	0.17 (0.12, 0.22)	<0.0001	57964	0.37 (0.28, 0.46)	<0.0001
Waist, cm	67779	0.87 (0.72, 1.03)	<0.0001	57965	1.87 (1.60, 2.14)	<0.0001
Hip, cm	67779	0.12 (0.02, 0.22)	0.016	57965	0.58 (0.41, 0.75)	<0.0001
WHR, %	67779	0.86 (0.75, 0.97)	<0.0001	57965	1.51 (1.33, 1.70)	<0.0001
Weight, kg	67778	0.67 (0.51, 0.83)	<0.0001	57964	1.39 (1.11, 1.67)	<0.0001
Body fat, %	67779	0.42 (0.32, 0.52)	<0.0001	57929	0.97 (0.79, 1.15)	<0.0001
Height, cm	67779	0.22 (0.12, 0.31)	<0.0001	57965	0.52 (0.35, 0.69)	<0.0001
Sitting height, cm	67779	0.00 (-0.05, 0.06)	0.86	57965	0.16 (0.06, 0.26)	0.0015
Glucose, mmol/L	64981	0.12 (0.09, 0.15)	<0.0001	55776	0.15 (0.09, 0.21)	<0.0001
HDL-C, mmol/L	2775	0.19 (0.17, 0.22)	<0.0001	9040	0.16 (0.13, 0.19)	<0.0001
LDL-C, mmol/L	2775	-0.10 (-0.16, -0.05)	0.0003	9040	-0.03 (-0.1, 0.03)	0.28
Ln (TG)	2775	0.14 (0.09, 0.19)	<0.0001	9040	0.10 (0.04, 0.15)	0.0007
Lp(a), nmol/L	2775	-6.20 (-10.09, -2.32)	0.0018	9040	0.48 (-3.81, 4.77)	0.83
CRP, mg/L	2775	1.08 (0.41, 1.76)	0.0018	9040	-0.12 (-0.75, 0.51)	0.72
Fibrinogen, g/L	1497	-0.09 (-0.17, 0.00)	0.038	4728	-0.23 (-0.32, -0.14)	<0.0001
GGT, IU/L	2588	61.7 (53.7, 69.7)	<0.0001	8386	44.3 (36.1, 52.5)	<0.0001
**b) Women**						
SBP, mmHg	5897	6.67 (4.30, 9.04)	<0.0001	83017	-0.55 (-1.02, -0.08)	0.022
DBP, mmHg	5897	3.78 (2.49, 5.06)	<0.0001	83017	0.11 (-0.14, 0.36)	0.40
Heart rate, bpm	6026	2.43 (1.03, 3.82)	0.0006	85945	-0.38 (-0.66, -0.11)	0.0069
BMI, kg/m^2	6026	-0.05 (-0.46, 0.35)	0.79	85945	0.11 (0.03, 0.19)	0.0083
Waist, cm	6026	0.77 (-0.33, 1.87)	0.17	85945	0.43 (0.21, 0.65)	0.0001
Hip, cm	6026	-0.72 (-1.47, 0.04)	0.062	85945	0.35 (0.20, 0.50)	<0.0001
WHR, %	6026	1.58 (0.78, 2.38)	0.0001	85945	0.13 (-0.03, 0.29)	0.11
Weight, kg	6026	-0.16 (-1.23, 0.91)	0.77	85945	0.57 (0.36, 0.78)	<0.0001
Body fat, %	6026	0.25 (-0.59, 1.09)	0.56	85903	0.17 (0.00, 0.34)	0.056
Height, cm	6026	-0.19 (-0.83, 0.45)	0.55	85945	0.44 (0.31, 0.57)	<0.0001
Sitting height, cm	6026	-0.35 (-0.73, 0.03)	0.068	85945	0.21 (0.13, 0.28)	<0.0001
Glucose, mmol/L	5699	0.18 (-0.05, 0.41)	0.12	82657	-0.07 (-0.12, -0.02)	0.0078
HDL-C, mmol/L	196	0.41 (0.22, 0.60)	<0.0001	8754	0.00 (-0.03, 0.03)	0.96
LDL-C, mmol/L	196	-0.19 (-0.64, 0.27)	0.42	8754	-0.06 (-0.12, 0.01)	0.095
Ln (TG)	196	-0.16 (-0.54, 0.22)	0.41	8752	-0.03 (-0.09, 0.02)	0.22
Lp(a), nmol/L	196	-2.88 (-36.17, 30.41)	0.87	8754	1.96 (-2.88, 6.81)	0.43
CRP, mg/L	196	-0.56 (-4.66, 3.53)	0.79	8754	0.34 (-0.15, 0.83)	0.18
Fibrinogen, g/L	100	-0.41 (-1.04, 0.23)	0.21	4485	-0.04 (-0.13, 0.05)	0.37
GGT, IU/L	185	30.2 (6.3, 54.0)	0.013	8088	-0.2 (-3.0, 2.7)	0.91

[1] Adjusted for area, age, education, income and smoking. Usual alcohol intake is calculated from the re-survey in 2008 for baseline traits; [2] Age-adjusted inverse-variance-weighted meta-analysis across 10 areas

Abbreviations: SBP systolic blood pressure; DBP diastolic blood pressure; BMI body mass index; WHR waist to hip ratio; HDL-C high-density-lipoprotein-cholesterol; LDL-C low-density-lipoprotein cholesterol; Ln (TG) $\log_e$ triglycerides (mmol/l); CRP C-reactive protein; GGT gamma-glutamyl transferase (in international units/L).

Text-Figure 4: Associations of stroke incidence with drinking patterns and with genotypic determinants of alcohol intake in men

I. Conventional Epidemiology

Relative risk, adjusted for area, age and covariates, by self-reported alcohol intake. Black: ever ≥weekly (with RR=1 for lowest current), white: never ≥weekly

II. Genetic epidemiology

Relative risk, adjusted for area and age but no covariates by genotype-predicted mean alcohol intake (in 6 categories, with RR=1 for category C1): Mendelian randomisation

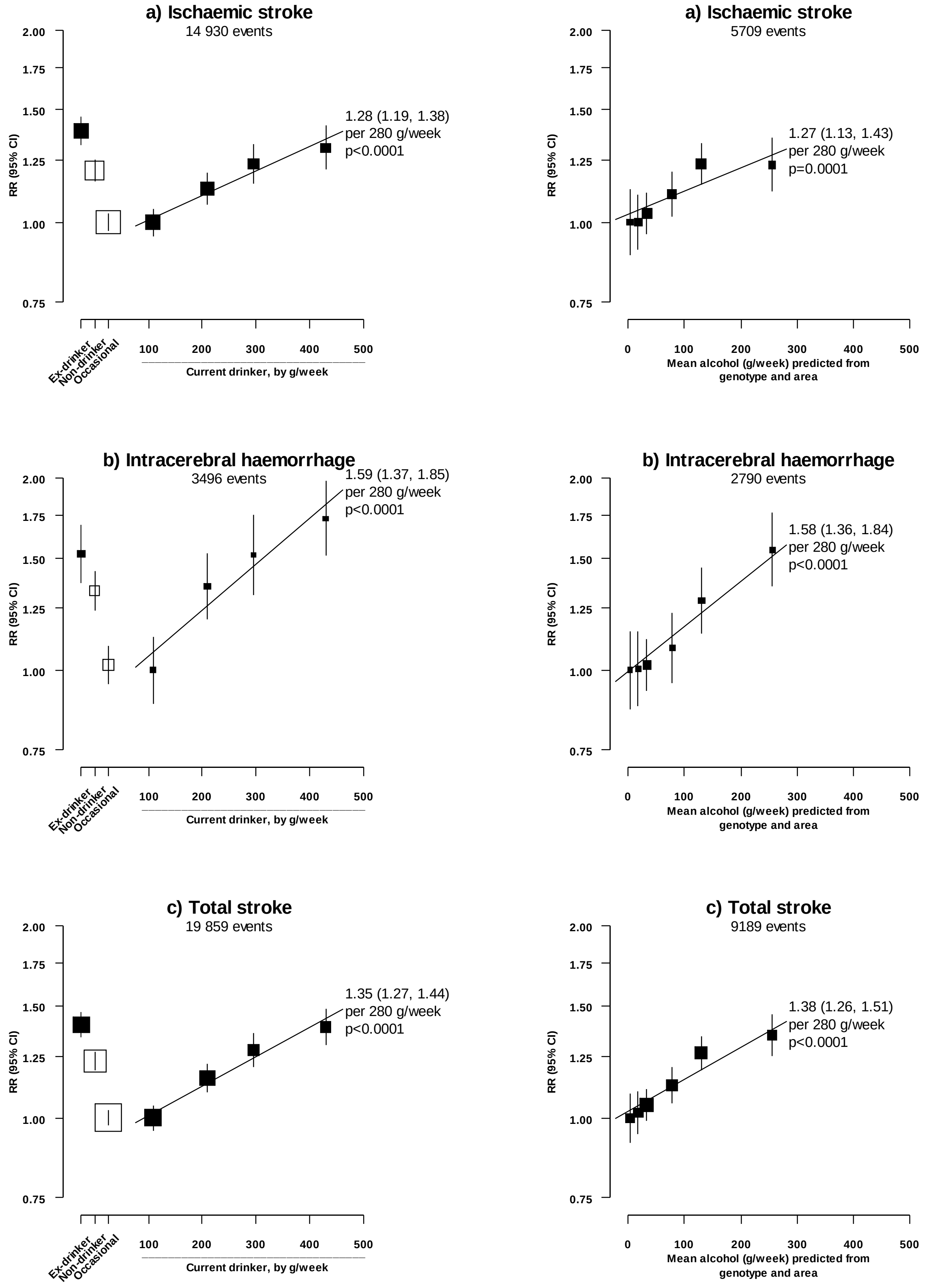

Webtable 11: Conventional epidemiologic associations of stroke incidence with alcohol intake – various models

	Ischaemic stroke		Intracerebral haemorrhage		Total stroke	
	**N events**	**RR (95% CI)**	**N events**	**RR (95% CI)**	**N events**	**RR (95% CI)**
Men, Model 1: Adjust for area, age, education, income and smoking, and exclude prior CVD						
Ex-drinker	1621	1.39 (1.32, 1.46)	405	1.52 (1.38, 1.68)	2190	1.40 (1.34, 1.46)
Non-drinker	3266	1.21 (1.16, 1.25)	1011	1.33 (1.25, 1.43)	4620	1.23 (1.19, 1.27)
Occasional drinker	5276	1.00 (0.97, 1.03)	1040	1.02 (0.96, 1.09)	6780	1.00 (0.98, 1.03)
Current drinker, <140 g/week	1831	1.00 (0.95, 1.05)	288	1.00 (0.89, 1.12)	2281	1.00 (0.96, 1.04)
140-279 g/week	1387	1.13 (1.07, 1.19)	296	1.35 (1.21, 1.52)	1805	1.16 (1.10, 1.21)
280-419 g/week	835	1.23 (1.15, 1.32)	203	1.52 (1.32, 1.74)	1125	1.28 (1.20, 1.36)
≥420 g/week	714	1.31 (1.21, 1.41)	253	1.73 (1.52, 1.97)	1058	1.39 (1.31, 1.48)
RR (95% CI) per 280 g/week usual alcohol intake (among current drinkers), and P-value	*4767*	*1.28 (1.19, 1.38) <0.0001*	*1040*	*1.59 (1.37, 1.85) <0.0001*	*6269*	*1.35 (1.27, 1.44) <0.0001*
Men, Model 2: As Model 1, but additionally exclude those with any prior disease or poor self-reported health, and events from the first 3 years follow-up						
Ex-drinker	670	1.39 (1.29, 1.50)	140	1.49 (1.26, 1.76)	859	1.37 (1.28, 1.47)
Non-drinker	1572	1.22 (1.15, 1.29)	384	1.21 (1.09, 1.35)	2099	1.21 (1.15, 1.26)
Occasional drinker	2795	1.00 (0.96, 1.04)	514	1.09 (1.00, 1.20)	3553	1.01 (0.98, 1.05)
Current drinker, <140 g/week	958	1.00 (0.94, 1.07)	134	1.00 (0.84, 1.19)	1178	1.00 (0.94, 1.06)
140-279 g/week	758	1.12 (1.04, 1.20)	136	1.33 (1.12, 1.58)	959	1.13 (1.06, 1.21)
280-419 g/week	476	1.22 (1.11, 1.33)	100	1.59 (1.30, 1.94)	623	1.27 (1.17, 1.37)
≥420 g/week	415	1.27 (1.15, 1.41)	141	2.13 (1.78, 2.54)	606	1.42 (1.30, 1.54)
RR (95% CI) per 280 g/week usual alcohol intake (among current drinkers), and P-value	*2607*	*1.25 (1.14, 1.38) <0.0001*	*511*	*1.91 (1.56, 2.36) <0.0001*	*3366*	*1.36 (1.25, 1.49) <0.0001*

(continued on next page)

Conventional epidemiologic associations of stroke incidence with alcohol intake – various models *(Webtable 11 continued)*

	Ischaemic stroke		Intracerebral haemorrhage		Total stroke	
	**N events**	**RR (95% CI)**	**N events**	**RR (95% CI)**	**N events**	**RR (95% CI)**
Men, Model 3: As Model 1, but additionally exclude ever-regular smokers						
Ex-drinker	260	1.47 (1.30, 1.67)	64	1.52 (1.19, 1.95)	355	1.47 (1.32, 1.63)
Non-drinker	1190	1.36 (1.27, 1.45)	291	1.25 (1.10, 1.42)	1617	1.35 (1.27, 1.42)
Occasional drinker	1745	1.10 (1.05, 1.16)	323	1.08 (0.96, 1.21)	2212	1.08 (1.03, 1.13)
Current drinker, <140 g/week	371	1.00 (0.90, 1.11)	58	1.00 (0.77, 1.30)	470	1.00 (0.91, 1.10)
140-279 g/week	193	1.27 (1.10, 1.47)	45	1.38 (1.02, 1.85)	252	1.24 (1.10, 1.41)
280-419 g/week	85	1.30 (1.05, 1.61)	16	1.01 (0.61, 1.65)	109	1.22 (1.01, 1.47)
≥420 g/week	50	1.12 (0.85, 1.48)	19	1.29 (0.81, 2.04)	78	1.19 (0.95, 1.49)
RR (95% CI) per 280 g/week usual alcohol intake (among current drinkers), and P-value	*699*	*1.26 (1.01, 1.57)* *0.04*	*138*	*1.21 (0.78, 1.87)* *0.40*	*909*	*1.24 (1.03, 1.5)* *0.02*
Men, Model 4: As Model 1, but restrict to the subset of genotyped individuals						
Ex-drinker	639	1.46 (1.35, 1.58)	336	1.64 (1.47, 1.83)	1062	1.49 (1.40, 1.58)
Non-drinker	1189	1.22 (1.14, 1.29)	822	1.43 (1.33, 1.54)	2174	1.26 (1.20, 1.32)
Occasional drinker	1969	1.00 (0.95, 1.05)	818	1.04 (0.97, 1.12)	2994	1.00 (0.96, 1.04)
Current drinker, <140 g/week	739	1.00 (0.93, 1.08)	229	1.00 (0.88, 1.14)	1047	1.00 (0.94, 1.06)
140-279 g/week	535	1.10 (1.01, 1.20)	233	1.36 (1.20, 1.55)	822	1.15 (1.07, 1.23)
280-419 g/week	346	1.27 (1.15, 1.42)	153	1.43 (1.22, 1.68)	549	1.32 (1.21, 1.44)
≥420 g/week	292	1.34 (1.19, 1.50)	199	1.70 (1.47, 1.97)	541	1.44 (1.31, 1.57)
RR (95% CI) per 280 g/week usual alcohol intake (among current drinkers), and P-value	*1912*	*1.32 (1.17, 1.48)* *<0.0001*	*814*	*1.55 (1.31, 1.83)* *<0.0001*	*2959*	*1.39 (1.27, 1.52)* *<0.0001*
Women, Model 1: As Model 1 for men						
Ex drinker	190	1.23 (1.06, 1.42)	41	1.26 (0.92, 1.73)	257	1.23 (1.08, 1.39)
Non-drinker	11283	1.15 (1.12, 1.18)	2225	1.33 (1.26, 1.41)	14544	1.14 (1.12, 1.17)
Occasional drinker	6653	1.03 (1.00, 1.06)	811	1.13 (1.05, 1.22)	8074	1.03 (1.00, 1.05)
Current drinker, <70 g/week	240	1.00 (0.88, 1.14)	24	1.00 (0.67, 1.49)	290	1.00 (0.89, 1.12)
70+ g/week	175	1.00 (0.86, 1.17)	35	1.09 (0.78, 1.53)	240	1.04 (0.92, 1.19)
RR (95% CI) per 280 g/week usual alcohol intake (among current drinkers), and P-value	*415*	*1.01 (0.58, 1.77)* *0.96*	*59*	*1.28 (0.29, 5.67)* *0.75*	*530*	*1.12 (0.69, 1.84)* *0.64*

Webtable 12: Genetic epidemiologic associations of stroke incidence with alcohol intake – various models

	Ischaemic stroke		Intracerebral haemorrhage		Total stroke	
	**N events**	**RR (95% CI)**	**N events**	**RR (95% CI)**	**N events**	**RR (95% CI)**
Men, Model 1: Adjust for area and age, and exclude prior CVD						
Category C1 (4 g/week mean male intake)	309	1.00 (0.89, 1.12)	223	1.00 (0.87, 1.15)	563	1.00 (0.92, 1.09)
Category C2 (19 g/week mean male intake)	638	1.00 (0.91, 1.10)	306	1.01 (0.88, 1.14)	1003	1.02 (0.95, 1.10)
Category C3 (34 g/week mean male intake)	927	1.03 (0.96, 1.11)	589	1.02 (0.93, 1.11)	1656	1.05 (0.99, 1.11)
Category C4 (78 g/week mean male intake)	1518	1.11 (1.02, 1.20)	540	1.08 (0.96, 1.22)	2215	1.13 (1.06, 1.20)
Category C5 (130 g/week mean male intake)	1426	1.23 (1.15, 1.33)	533	1.29 (1.15, 1.44)	2107	1.27 (1.19, 1.34)
Category C6 (255 g/week mean male intake)	891	1.23 (1.12, 1.35)	599	1.54 (1.36, 1.76)	1645	1.35 (1.26, 1.45)
RR (95% CI) per 280 g/week genotype-predicted mean male intake, and P-value[1]	*5709*	*1.27 (1.13, 1.43)* *0.0001*	*2790*	*1.58 (1.36, 1.84)* *<0.0001*	*9189*	*1.38 (1.26, 1.51)* *<0.0001*
Men, Model 2: As Model 1, but additionally adjust for education, income and smoking						
Category C1 (4 g/week mean male intake)	309	1.00 (0.89, 1.12)	223	1.00 (0.87, 1.15)	563	1.00 (0.92, 1.09)
Category C2 (19 g/week mean male intake)	638	1.00 (0.91, 1.10)	306	1.01 (0.89, 1.15)	1003	1.02 (0.95, 1.10)
Category C3 (34 g/week mean male intake)	927	1.02 (0.95, 1.10)	589	1.03 (0.94, 1.12)	1656	1.05 (0.99, 1.10)
Category C4 (78 g/week mean male intake)	1518	1.11 (1.02, 1.20)	540	1.09 (0.97, 1.23)	2215	1.13 (1.06, 1.20)
Category C5 (130 g/week mean male intake)	1426	1.23 (1.15, 1.32)	533	1.30 (1.16, 1.45)	2107	1.27 (1.20, 1.34)
Category C6 (255 g/week mean male intake)	891	1.23 (1.12, 1.35)	599	1.55 (1.37, 1.77)	1645	1.35 (1.26, 1.45)
RR (95% CI) per 280 g/week genotype-predicted mean male intake, and P-value[1]	*5709*	*1.28 (1.13, 1.44)* *0.0001*	*2790*	*1.59 (1.36, 1.84)* *<0.0001*	*9189*	*1.38 (1.26, 1.51)* *<0.0001*
Women, Model 1: As Model 1 for men (relating female risk in C1-C6 to mean MALE intake)						
Category C1 (4 g/week mean MALE intake)	462	1.00 (0.91, 1.10)	231	1.00 (0.87, 1.14)	745	1.00 (0.93, 1.08)
Category C2 (19 g/week mean MALE intake)	830	0.93 (0.86, 1.02)	302	1.05 (0.93, 1.19)	1213	0.99 (0.92, 1.05)
Category C3 (34 g/week mean MALE intake)	1307	0.98 (0.92, 1.04)	629	1.16 (1.06, 1.26)	2102	1.04 (0.99, 1.09)
Category C4 (78 g/week mean MALE intake)	1698	0.93 (0.87, 1.00)	502	1.21 (1.07, 1.38)	2398	1.01 (0.95, 1.07)
Category C5 (130 g/week mean MALE intake)	1754	0.96 (0.90, 1.02)	409	1.19 (1.04, 1.35)	2359	1.02 (0.97, 1.08)
Category C6 (255 g/week mean MALE intake)	1042	0.95 (0.88, 1.04)	452	1.06 (0.93, 1.22)	1685	0.99 (0.93, 1.06)
RR (95% CI) per 280 g/week genotype-predicted mean male intake, and P-value[1]	*7093*	*0.98 (0.88, 1.09)* *0.69*	*2525*	*0.96 (0.82, 1.12)* *0.62*	*10 502*	*0.98 (0.9, 1.06)* *0.61*

[1] From an inverse-variance-weighted meta-analysis across ten areas, adjusted for age.

Webtable 13: Genetic epidemiology – CVD incidence rate ratios in men, comparing categories C4-C5 (moderate mean alcohol intake) versus C1-C3 (low mean alcohol intake)

	**Events in categories C1-C3** (mean intake 23.5 g/week)	**Events in categories C4-C5** (mean intake 98.7 g/week)	**Incidence rate ratio, C4-C5 / C1-C3** RR and 95% CI
Ischaemic stroke	1874	2944	1.16 (1.09, 1.25)
Intracerebral haemorrhage	1118	1073	1.21 (1.09, 1.35)
Total stroke	3222	4322	1.18 (1.12, 1.25)
Acute myocardial infarction	695	688	0.89 (0.78, 1.02)
Total CHD	2201	2787	0.94 (0.88, 1.00)

[1] Although the categories are defined by study area and genotype, these analyses are of within-area genotypic effects, and are adjusted for area and age (and exclude men with prior CVD at baseline).

Webfigure 2: Associations within each separate area of genotypic categories C1-C6 and SBP, in men

Analyses are adjusted for age

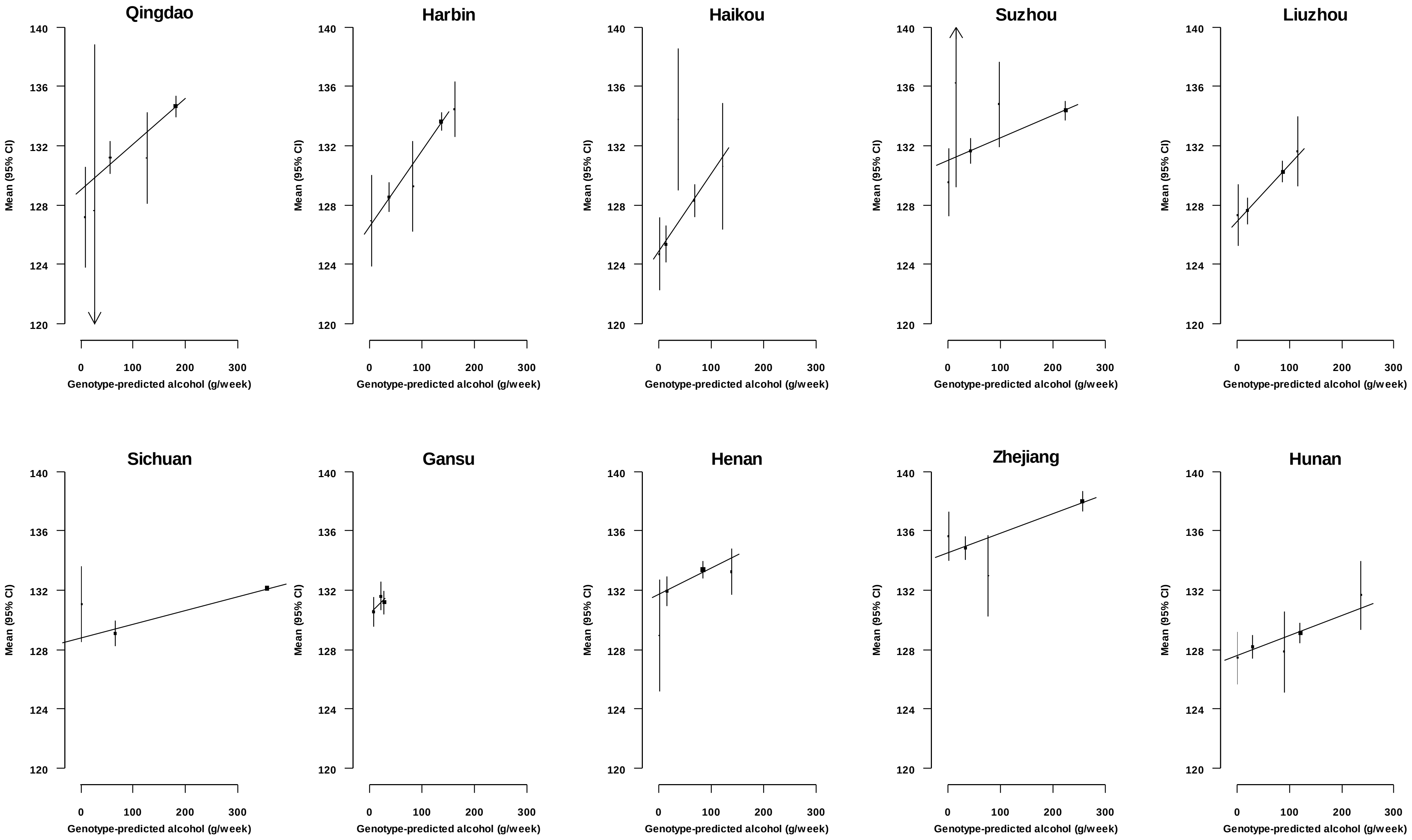

Webfigure 3: Associations within each separate area of genotypic categories C1-C6 and total stroke incidence, in men

Due to small numbers of events, categories C1-C3 have been merged as the reference category. In some areas, one of C4, C5 or C6 is not present and in Gansu none were present (Figure 1)
Analyses are adjusted for age

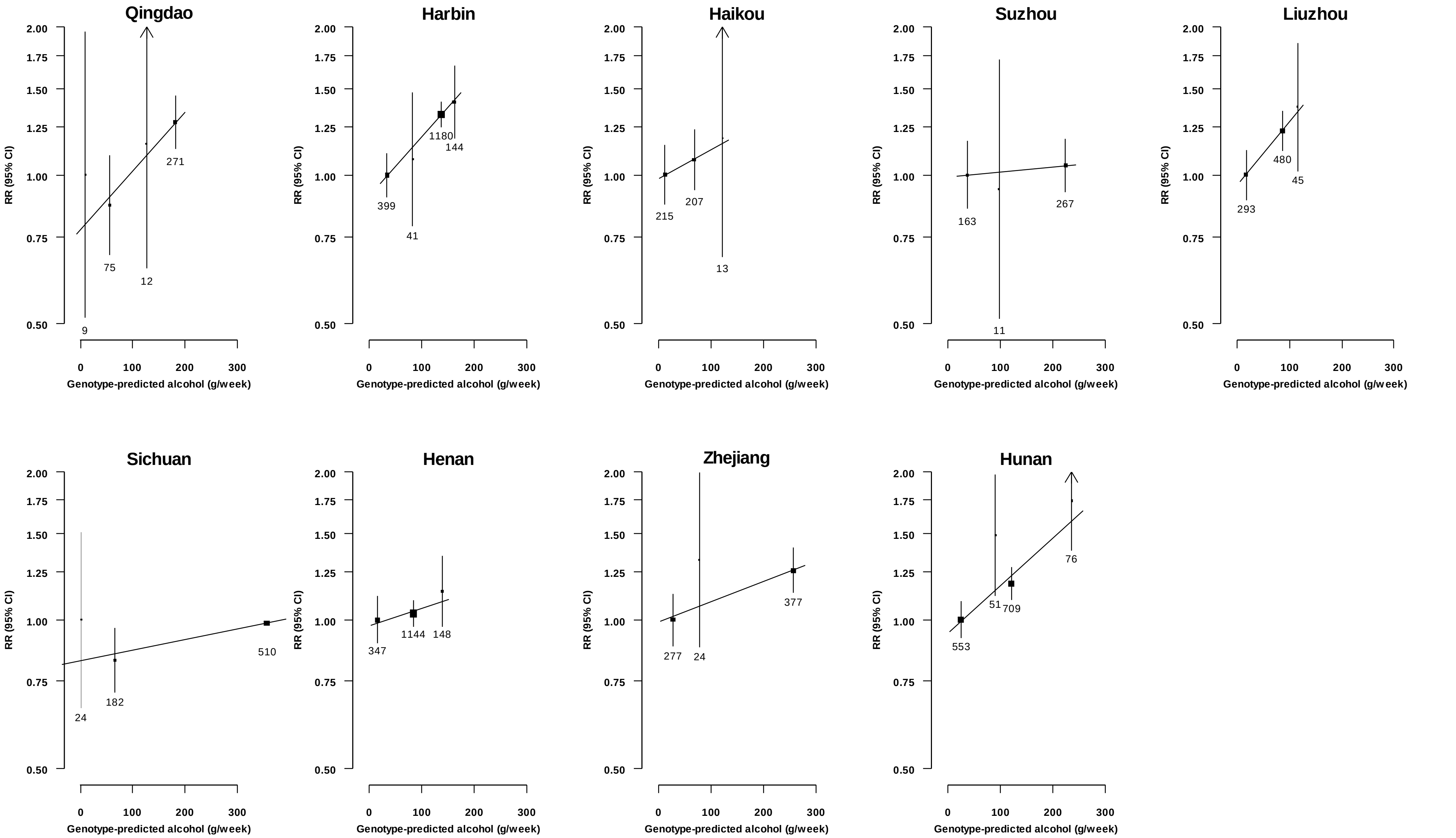

Webfigure 4: Age at baseline vs percent drinking and mean intake among men in categories C1-3, C4, C5 and C6 of genotype and area

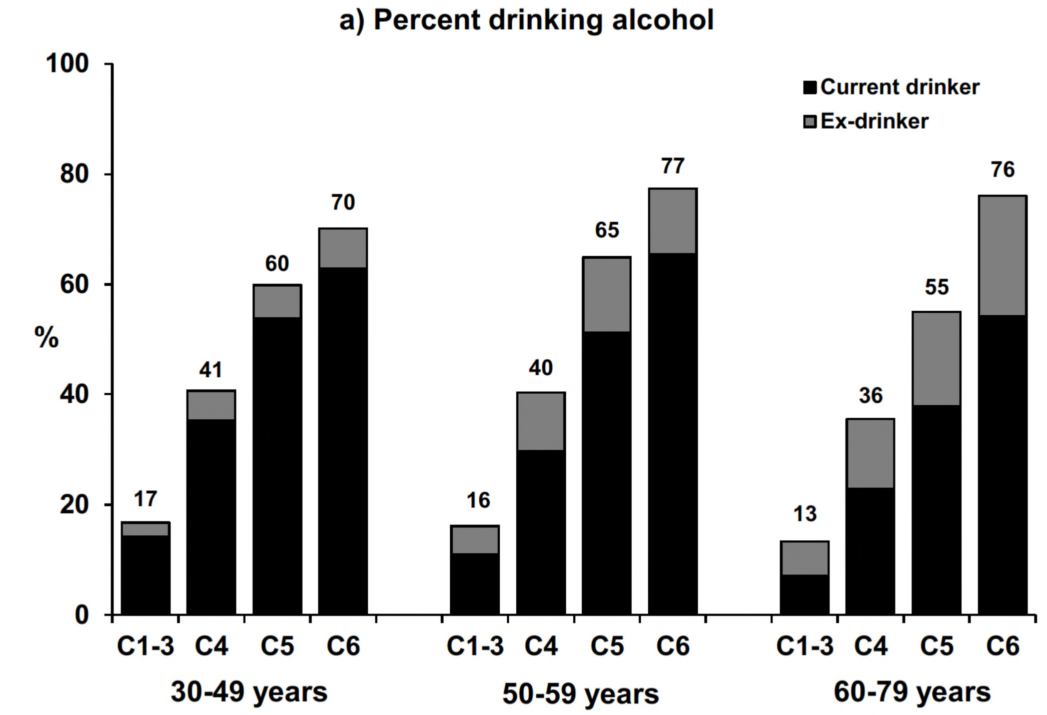

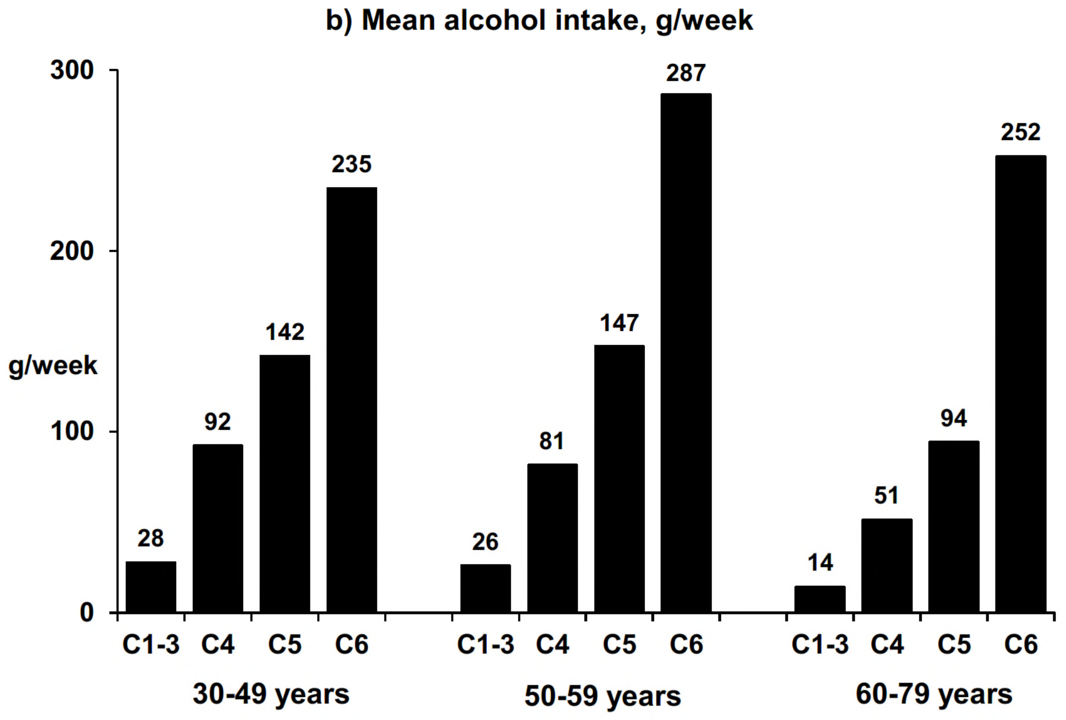

Webtable 14: **Relevance of age at baseline to the associations of alcohol intake with SBP, stroke and myocardial infarction among men**

	Conventional epidemiology[1]			Genetic epidemiology[2]		
	**Number of participants or events**	**Slope or RR (95% CI) per 280 g/week usual intake in current drinkers**	**P-value**	**Number of participants or events**	**Slope or RR (95% CI) per 280 g/week genotype-predicted mean male alcohol intake**	**P-value**
**SBP (mmHg)**						
30-49 years	31 792	5.50 (5.09, 5.92)	<0.0001	24 945	3.78 (3.06, 4.49)	<0.0001
50-59 years	20 852	4.63 (4.05, 5.20)	<0.0001	17 112	5.13 (4.11, 6.15)	<0.0001
60-79 years	13 427	3.61 (2.79, 4.43)	<0.0001	13 840	3.63 (2.29, 4.96)	<0.0001
**Ischaemic stroke**						
30-49 years	1211	1.37 (1.20, 1.57)	<0.0001	1245	1.42 (1.05, 1.93)	0.02
50-59 years	1607	1.29 (1.13, 1.47)	0.0001	1769	1.23 (0.99, 1.52)	0.07
60-79 years	1949	1.19 (1.04, 1.36)	0.009	2695	1.16 (0.98, 1.36)	0.08
**Intracerebral haemorrhage**						
30-49 years	261	1.79 (1.36, 2.36)	<0.0001	497	1.82 (1.22, 2.73)	0.003
50-59 years	337	1.65 (1.28, 2.14)	0.0001	809	1.77 (1.32, 2.38)	0.0001
60-79 years	442	1.49 (1.15, 1.92)	0.002	1484	1.31 (1.07, 1.59)	0.008
**Acute myocardial infarction**						
30-49 years	193	1.35 (0.98, 1.86)	0.06	341	0.81 (0.53, 1.25)	0.35
50-59 years	245	1.27 (0.93, 1.73)	0.14	526	0.83 (0.57, 1.21)	0.33
60-79 years	320	0.92 (0.64, 1.32)	0.65	829	1.00 (0.73, 1.37)	0.99

[1] Adjusted for area, age, education, income and smoking. Usual alcohol intake is calculated from the resurvey in 2008 for SBP, and from an average of resurveys in 2008 and 2013-14 for events during the ten-year follow-up.

[2] From an inverse-variance-weighted meta-analysis across ten areas, adjusted for age.

Text-Figure 5: Associations of coronary heart disease incidence with drinking patterns and with genotypic determinants of alcohol intake in men

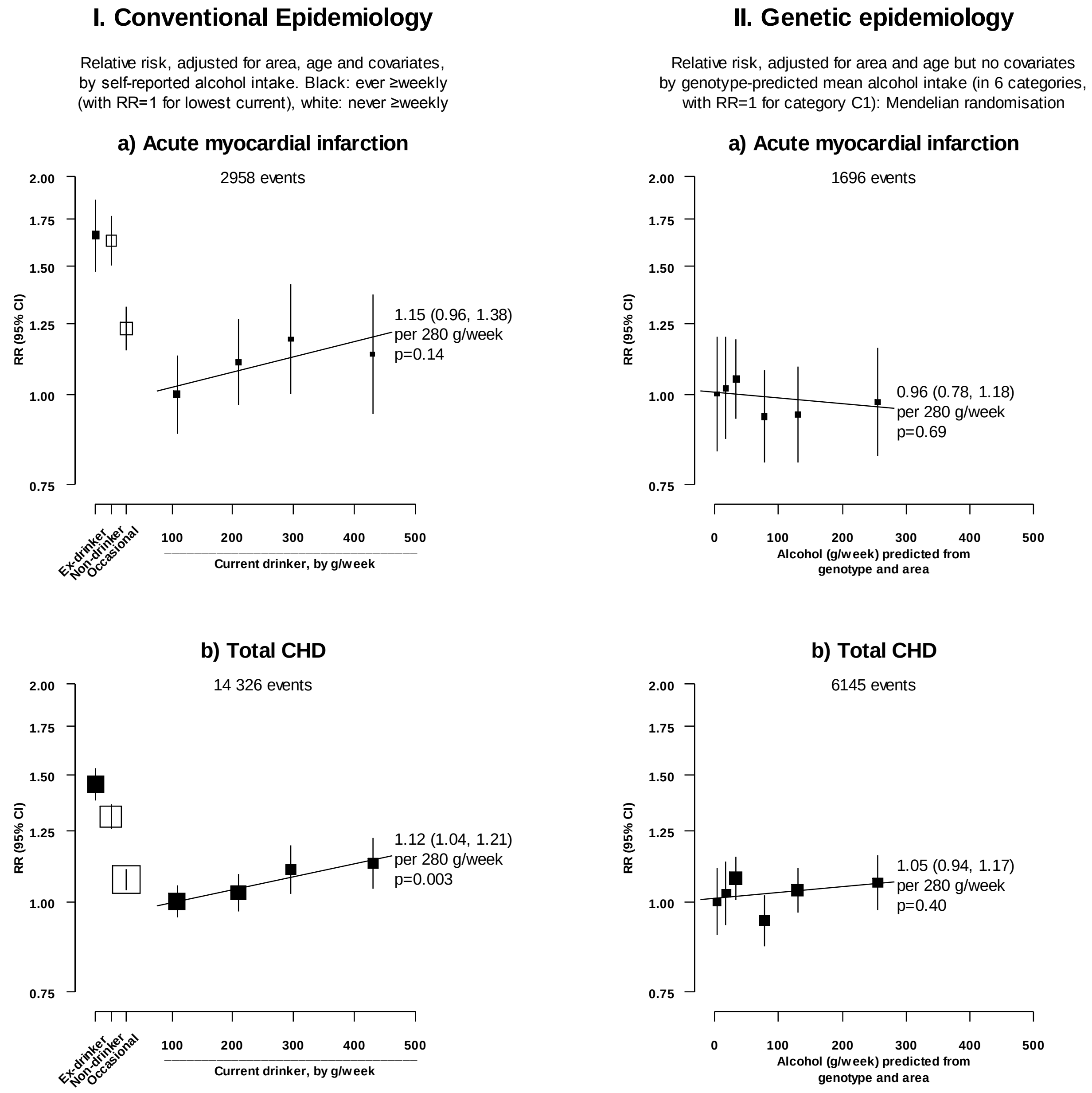

Webtable 15: Conventional epidemiologic associations of CHD with alcohol intake – various models

	Acute myocardial infarction		Total coronary heart disease	
	**N events**	**RR (95% CI)**	**N events**	**RR (95% CI)**
Men, Model 1: Adjust for area, age, education, income and smoking, and exclude prior CVD				
Ex-drinker	329	1.66 (1.48, 1.85)	1697	1.45 (1.38, 1.52)
Non-drinker	840	1.63 (1.51, 1.76)	3234	1.31 (1.26, 1.36)
Occasional drinker	1031	1.23 (1.16, 1.31)	4915	1.07 (1.04, 1.11)
Current drinker, <140 g/week	273	1.00 (0.89, 1.13)	1766	1.00 (0.95, 1.05)
140-279 g/week	225	1.11 (0.97, 1.26)	1287	1.03 (0.97, 1.09)
280-419 g/week	138	1.19 (1.01, 1.41)	756	1.11 (1.03, 1.19)
≥420 g/week	122	1.14 (0.95, 1.37)	671	1.13 (1.04, 1.22)
RR (95% CI) per 280 g/week usual alcohol intake (among current drinkers), and P-value	*758*	*1.15 (0.96, 1.38)* *0.14*	*4480*	*1.12 (1.04, 1.21)* *0.003*
Men, Model 2: As Model 1, but additionally exclude those with any prior disease or poor self-reported health, and events from the first 3 years follow-up				
Ex-drinker	134	1.57 (1.32, 1.87)	628	1.30 (1.20, 1.41)
Non-drinker	425	1.66 (1.50, 1.85)	1473	1.24 (1.17, 1.31)
Occasional drinker	572	1.30 (1.20, 1.42)	2523	1.05 (1.00, 1.09)
Current drinker, <140 g/week	145	1.00 (0.85, 1.18)	898	1.00 (0.94, 1.07)
140-279 g/week	136	1.18 (0.99, 1.40)	700	1.04 (0.96, 1.12)
280-419 g/week	84	1.24 (1.00, 1.55)	451	1.17 (1.07, 1.29)
≥420 g/week	61	0.97 (0.75, 1.26)	381	1.11 (1.00, 1.23)
RR (95% CI) per 280 g/week usual alcohol intake (among current drinkers), and P-value	*426*	*1.04 (0.81, 1.34)* *0.76*	*2430*	*1.13 (1.02, 1.25)* *0.02*

(continued on next page)

Conventional epidemiologic associations of CHD incidence with alcohol intake – various models *(Webtable 15 continued)*

	Acute myocardial infarction		Total coronary heart disease	
	**N events**	**RR (95% CI)**	**N events**	**RR (95% CI)**
Men, Model 3: As Model 1, but additionally exclude ever-regular smokers				
Ex-drinker	48	1.85 (1.39, 2.47)	261	1.49 (1.31, 1.68)
Non-drinker	235	1.56 (1.36, 1.80)	1003	1.30 (1.21, 1.39)
Occasional drinker	266	1.23 (1.08, 1.39)	1533	1.09 (1.04, 1.15)
Current drinker, <140 g/week	43	1.00 (0.74, 1.36)	369	1.00 (0.90, 1.11)
140-279 g/week	22	1.12 (0.73, 1.71)	170	1.05 (0.90, 1.23)
280-419 g/week	14	1.71 (1.01, 2.90)	62	0.93 (0.72, 1.20)
≥420 g/week	10	1.51 (0.80, 2.84)	69	1.43 (1.12, 1.82)
RR (95% CI) per 280 g/week usual alcohol intake (among current drinkers), and P-value	*89*	*1.60 (0.92, 2.78)*<br>*0.10*	*670*	*1.25 (1.02, 1.54)*<br>*0.04*
Men, Model 4: As Model 1, but restrict to the subset of genotyped individuals				
Ex-drinker	185	1.60 (1.38, 1.85)	781	1.65 (1.54, 1.77)
Non-drinker	474	1.60 (1.45, 1.77)	1495	1.45 (1.37, 1.53)
Occasional drinker	583	1.16 (1.06, 1.26)	2036	1.13 (1.08, 1.18)
Current drinker, <140 g/week	172	1.00 (0.86, 1.16)	737	1.00 (0.93, 1.08)
140-279 g/week	133	1.07 (0.90, 1.27)	522	1.03 (0.94, 1.12)
280-419 g/week	68	0.92 (0.72, 1.17)	282	1.01 (0.89, 1.13)
≥420 g/week	81	1.17 (0.93, 1.46)	292	1.18 (1.05, 1.33)
RR (95% CI) per 280 g/week usual alcohol intake (among current drinkers), and P-value	*454*	*1.10 (0.87, 1.38)*<br>*0.44*	*1833*	*1.13 (1.00, 1.27)*<br>*0.04*
Women, Model 1: As Model 1 for men				
Ex drinker	32	1.57 (1.10, 2.25)	292	1.12 (0.99, 1.26)
Non-drinker	1601	1.49 (1.40, 1.59)	12268	1.07 (1.05, 1.10)
Occasional drinker	589	1.15 (1.05, 1.25)	7959	0.96 (0.94, 0.98)
Current drinker, <70 g/week	19	1.00 (0.64, 1.57)	331	1.00 (0.90, 1.11)
70+ g/week	26	1.19 (0.80, 1.77)	246	0.84 (0.74, 0.96)
RR (95% CI) per 280 g/week usual alcohol intake (among current drinkers), and P-value	*45*	*1.65 (0.30, 9.05)*<br>*0.56*	*577*	*0.61 (0.38, 0.98)*<br>*0.04*

Webtable 16: Genetic epidemiologic associations of CHD incidence with alcohol intake – various models

	Acute myocardial infarction		Total coronary heart disease	
	N events	RR (95% CI)	N events	RR (95% CI)
Men, Model 1: Adjust for area and age, and exclude prior CVD				
Category C1 (4 g/week mean male intake)	132	1.00 (0.84, 1.19)	379	1.00 (0.90, 1.11)
Category C2 (19 g/week mean male intake)	209	1.02 (0.87, 1.19)	607	1.03 (0.93, 1.13)
Category C3 (34 g/week mean male intake)	354	1.05 (0.93, 1.19)	1215	1.08 (1.01, 1.15)
Category C4 (78 g/week mean male intake)	383	0.93 (0.81, 1.07)	1278	0.94 (0.87, 1.02)
Category C5 (130 g/week mean male intake)	305	0.94 (0.81, 1.09)	1509	1.04 (0.97, 1.11)
Category C6 (255 g/week mean male intake)	313	0.97 (0.83, 1.15)	1157	1.06 (0.98, 1.15)
RR (95% CI) per 280 g/week genotype-predicted mean male intake, and P-value[1]	*1696*	*0.96 (0.78, 1.18)* *0.69*	*6145*	*1.05 (0.94, 1.17)* *0.40*
Men, Model 2: As Model 1, but additionally adjust for education, income and smoking				
Category C1 (4 g/week mean male intake)	132	1.00 (0.84, 1.19)	379	1.00 (0.90, 1.11)
Category C2 (19 g/week mean male intake)	209	1.01 (0.86, 1.19)	607	1.02 (0.93, 1.12)
Category C3 (34 g/week mean male intake)	354	1.04 (0.92, 1.18)	1215	1.07 (1.00, 1.14)
Category C4 (78 g/week mean male intake)	383	0.93 (0.80, 1.07)	1278	0.93 (0.86, 1.01)
Category C5 (130 g/week mean male intake)	305	0.94 (0.81, 1.09)	1509	1.03 (0.97, 1.11)
Category C6 (255 g/week mean male intake)	313	0.97 (0.82, 1.15)	1157	1.06 (0.97, 1.15)
RR (95% CI) per 280 g/week genotype-predicted mean male intake, and P-value[1]	*1696*	*0.97 (0.79, 1.20)* *0.80*	*6145*	*1.05 (0.94, 1.17)* *0.37*
Women, Model 1: As Model 1 for men (relating female risk in C1-C6 to mean MALE intake)				
Category C1 (4 g/week mean MALE intake)	116	1.00 (0.83, 1.21)	493	1.00 (0.91, 1.09)
Category C2 (19 g/week mean MALE intake)	174	0.96 (0.81, 1.13)	757	1.01 (0.92, 1.10)
Category C3 (34 g/week mean MALE intake)	244	0.99 (0.85, 1.15)	1380	0.97 (0.91, 1.03)
Category C4 (78 g/week mean MALE intake)	294	0.95 (0.81, 1.11)	1806	0.99 (0.93, 1.05)
Category C5 (130 g/week mean MALE intake)	207	1.02 (0.85, 1.22)	2046	0.99 (0.93, 1.05)
Category C6 (255 g/week mean MALE intake)	211	0.92 (0.75, 1.13)	1697	1.02 (0.95, 1.09)
RR (95% CI) per 280 g/week genotype-predicted mean male intake, and P-value[1]	*1246*	*0.94 (0.74, 1.20)* *0.62*	*8179*	*1.02 (0.93, 1.12)* *0.66*

[1] From an inverse-variance-weighted meta-analysis across ten areas, adjusted for age

Text-Table 2: Comparison between genotypic effects in men and in women

	**Systolic blood pressure (mm Hg)**		**HDL cholesterol (mmol/L)**		**Gamma-glutamyl transferase (IU/L)**		**Ischaemic stroke (RR)**		**Intracerebral haemorrhage (RR)**		**Acute myocardial infarction (RR)**	
Category of genotype and study area*	Men n= 55 879	Women n= 83 017	Men n= 9040	Women n= 8754	Men n= 8386	Women n= 8088	Men 5709 events	Women 7087 events	Men 2790 events	Women 2525 events	Men 1696 events	Women 1246 events
1	129.7	129.7	1.16	1.29	28	23	1.00	1.00	1.00	1.00	1.00	1.00
2	130.0	129.4	1.16	1.29	31	24	1.00	0.93	1.01	1.05	1.02	0.96
3	130.5	129.2	1.17	1.28	32	25	1.03	0.98	1.02	1.16	1.05	0.99
4	131.6	128.5	1.19	1.28	41	22	1.11	0.93	1.08	1.21	0.93	0.95
5	133.0	128.4	1.24	1.28	55	24	1.23	0.96	1.29	1.19	0.94	1.02
6	134.1	128.5	1.30	1.29	70	23	1.23	0.95	1.54	1.06	0.97	0.92
Effect per 280 g/week mean MALE alcohol intake, 95% CI, and p-value for effect being greater in men†	4.3<br>3.7, 4.9	-0.6<br>-1.0, -0.1	0.16<br>0.13, 0.19	0.00<br>-0.03, 0.03	44<br>36, 53	0<br>-3, 3	1.27<br>1.13, 1.43	0.98<br>0.88, 1.09	1.58<br>1.36, 1.84	0.96<br>0.82, 1.12	0.96<br>0.78, 1.18	0.94<br>0.74, 1.20
	p<0.0001		p<0.0001		p<0.0001		p=0.0007		p<0.0001		0.45	

*Six categories of genotype and study area; mean values of physiological factors and RRs of disease are adjusted for age and area, leaving genotypic differences.

†Genotypic effect on physiological factor (slope per 280 g per week mean MALE alcohol intake) or on disease incidence (RR per 280 g per week mean MALE alcohol intake; since women consumed little alcohol, comparison between these genotypic effects in men and in women can help assess whether the genotypic effects in men are chiefly mediated by alcohol rather than by pleiotropic pathways that influence both sexes similarly. RR=relative risk.

Webtable 17: Associations of *ALDH2*-rs671 and *ADH1B*-rs1229984 with physiological factors

	Number genotyped	*ALDH2*-rs671 GG vs AG difference (SE)[1]	*ALDH2*-rs671 Trend per G-allele (SE)[2]	*ALDH2*-rs671 P-value for trend	*ADH1B*-rs1229984 GG vs AG difference (SE)[1]	*ADH1B*-rs1229984 Trend per G-allele (SE)[2]	*ADH1B*-rs1229984 P-value for trend
**a) Men**							
SBP, mmHg	55897	2.55 (0.17)	2.10 (0.14)	<0.0001	0.88 (0.28)	0.46 (0.12)	0.0001
DBP, mmHg	55897	1.87 (0.10)	1.59 (0.08)	<0.0001	0.59 (0.17)	0.31 (0.07)	<0.0001
Heart rate, bpm	57965	1.15 (0.11)	0.83 (0.09)	<0.0001	0.32 (0.18)	0.21 (0.08)	0.0073
BMI, kg/m^2	57964	0.24 (0.03)	0.20 (0.02)	<0.0001	0.21 (0.05)	0.09 (0.02)	<0.0001
Waist, cm	57965	1.11 (0.08)	1.00 (0.07)	<0.0001	0.64 (0.14)	0.28 (0.06)	<0.0001
Hip, cm	57965	0.39 (0.05)	0.38 (0.04)	<0.0001	0.38 (0.09)	0.10 (0.04)	0.0090
WHR, %	57965	0.86 (0.06)	0.73 (0.05)	<0.0001	0.29 (0.09)	0.21 (0.04)	<0.0001
Weight, kg	57964	0.88 (0.09)	0.82 (0.07)	<0.0001	0.59 (0.14)	0.21 (0.06)	0.0008
Body fat, %	57929	0.56 (0.05)	0.49 (0.04)	<0.0001	0.36 (0.09)	0.12 (0.04)	0.0008
Height, cm	57965	0.28 (0.05)	0.36 (0.04)	<0.0001	0.00 (0.09)	-0.04 (0.04)	0.29
Sitting height, cm	57965	0.08 (0.03)	0.12 (0.02)	<0.0001	0.12 (0.05)	0.02 (0.02)	0.27
Glucose, mmol/L	55776	0.13 (0.02)	0.09 (0.01)	<0.0001	0.07 (0.03)	0.00 (0.01)	0.80
HDL-C, mmol/L	9040	0.07 (0.01)	0.05 (0.01)	<0.0001	0.04 (0.01)	0.01 (0.00)	0.017
LDL-C, mmol/L	9040	-0.01 (0.02)	-0.02 (0.01)	0.22	0.08 (0.02)	0.03 (0.01)	0.0014
Ln (TG)	9040	0.07 (0.02)	0.05 (0.01)	<0.0001	0.00 (0.02)	-0.01 (0.01)	0.22
Lp(a), nmol/L	9040	0.57 (1.14)	0.54 (0.86)	0.53	-4.12 (1.69)	-0.79 (0.71)	0.27
CRP, mg/L	9040	-0.04 (0.20)	-0.02 (0.12)	0.87	0.27 (0.30)	0.11 (0.10)	0.27
Fibrinogen, g/L	4728	-0.12 (0.02)	-0.10 (0.02)	<0.0001	0.00 (0.04)	-0.02 (0.02)	0.27
GGT, IU/L	8386	21.71 (2.4)	8.42 (1.06)	<0.0001	11.68 (3.55)	1.41 (0.80)	0.078
**b) Women**							
SBP, mmHg	83017	-0.57 (0.15)	-0.54 (0.12)	<0.0001	0.00 (0.24)	0.00 (0.10)	0.98
DBP, mmHg	83017	-0.05 (0.08)	-0.07 (0.06)	0.30	0.11 (0.13)	0.00 (0.05)	0.99
Heart rate, bpm	85945	-0.23 (0.08)	-0.27 (0.07)	<0.0001	-0.04 (0.14)	-0.04 (0.06)	0.55
BMI, kg/m^2	85945	0.04 (0.02)	0.04 (0.02)	0.038	0.06 (0.04)	0.05 (0.02)	0.0029
Waist, cm	85945	0.16 (0.07)	0.18 (0.05)	0.0005	0.12 (0.11)	0.14 (0.05)	0.0025
Hip, cm	85945	0.19 (0.05)	0.21 (0.04)	<0.0001	0.01 (0.08)	0.06 (0.03)	0.068
WHR, %	85945	0.00 (0.05)	0.01 (0.04)	0.80	0.12 (0.08)	0.09 (0.03)	0.0053
Weight, kg	85945	0.23 (0.07)	0.31 (0.05)	<0.0001	0.07 (0.11)	0.06 (0.05)	0.16
Body fat, %	85903	0.06 (0.05)	0.07 (0.04)	0.086	0.16 (0.08)	0.11 (0.04)	0.0016
Height, cm	85945	0.18 (0.04)	0.28 (0.03)	<0.0001	-0.10 (0.06)	-0.09 (0.03)	0.0016
Sitting height, cm	85945	0.09 (0.02)	0.12 (0.02)	<0.0001	-0.03 (0.04)	-0.03 (0.02)	0.053
Glucose, mmol/L	82657	-0.01 (0.01)	-0.02 (0.01)	0.042	0.03 (0.02)	0.00 (0.01)	0.81
HDL-C, mmol/L	8754	0.00 (0.01)	0.00 (0.01)	0.67	0.02 (0.01)	-0.01 (0.00)	0.17
LDL-C, mmol/L	8754	-0.02 (0.02)	-0.01 (0.01)	0.55	-0.02 (0.03)	0.01 (0.01)	0.30
Ln (TG)	8752	-0.01 (0.01)	-0.01 (0.01)	0.27	-0.03 (0.02)	0.00 (0.01)	0.68
Lp(a), nmol/L	8754	-0.15 (1.28)	0.35 (0.92)	0.71	-0.29 (1.88)	0.85 (0.78)	0.27
CRP, mg/L	8754	0.15 (0.16)	0.13 (0.09)	0.14	-0.17 (0.23)	0.04 (0.08)	0.60
Fibrinogen, g/L	4485	-0.01 (0.02)	-0.01 (0.02)	0.40	-0.02 (0.04)	0.00 (0.01)	0.97
GGT, IU/L	8088	0.52 (0.93)	0.08 (0.48)	0.87	-1.32 (1.37)	-0.68 (0.37)	0.070

[1] Adjusted for age and area. [2] A meta-analysis of the age-adjusted results in each of the 10 study areas (ie, an inverse-variance-weighted average).

Abbreviations: SBP systolic blood pressure; DBP diastolic blood pressure; BMI body mass index; WHR waist to hip ratio; HDL-C high-density-lipoprotein-cholesterol; LDL-C low-density-lipoprotein cholesterol; Ln (TG) $\log_e$ triglycerides (mmol/l); CRP C-reactive protein; GGT gamma-glutamyl transferase (in international units/L

Webfigure 5: Associations of *ALDH2*-rs671 and *ADH1B*-rs1229984 with three physiological factors

Analyses are adjusted for area and age. Results are plotted at the mean MALE alcohol intake of each genotype, to facilitate the comparison of findings between men and women. The mean FEMALE alcohol intakes of rs671 AA/AG/GG are 0.6/1.9/5.1 g/week and of rs1229984 AA/AG/GG are 3.5/3.9/5.6 g/week

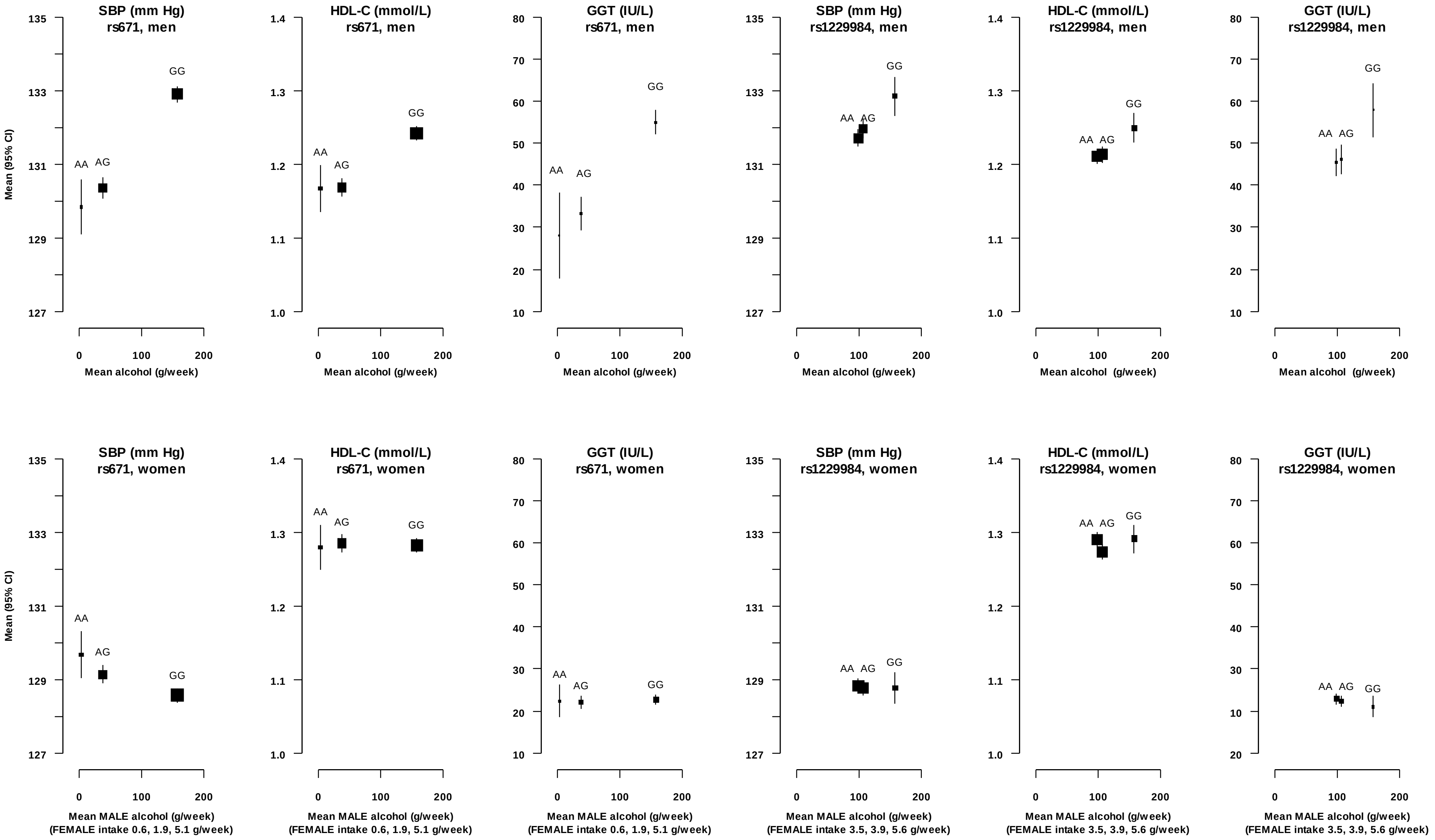

Webtable 18: Associations of *ALDH2*-rs671 and *ADH1B*-rs1229984 with cardiovascular disease incidence; reference group is the AG genotype

	ALDH2-rs671			*ADH1B*-rs122984		
	**Genotype**	**Events**	**RR (95% CI)** [1]	**Genotype**	**Events**	**RR (95% CI)** [1]
**a) Men**						
Ischaemic stroke	AA	223	1.04 (0.91, 1.18)	AA	2667	1.00 (0.97, 1.04)
	AG	1631	1.00 (0.95, 1.05)	AG	2450	1.00 (0.96, 1.04)
	GG	3855	1.15 (1.11, 1.19)	GG	592	1.13 (1.04, 1.23)
Intracerebral haemorrhage	AA	110	1.02 (0.84, 1.23)	AA	1292	1.00 (0.94, 1.05)
	AG	797	1.00 (0.93, 1.07)	AG	1171	1.00 (0.94, 1.06)
	GG	1883	1.24 (1.18, 1.30)	GG	327	1.32 (1.19, 1.47)
Total stroke	AA	359	1.02 (0.92, 1.13)	AA	4309	1.01 (0.98, 1.04)
	AG	2626	1.00 (0.96, 1.04)	AG	3898	1.00 (0.97, 1.03)
	GG	6204	1.19 (1.15, 1.22)	GG	982	1.19 (1.11, 1.26)
Myocardial infarction	AA	85	1.27 (1.03, 1.58)	AA	769	0.95 (0.88, 1.02)
	AG	520	1.00 (0.92, 1.09)	AG	745	1.00 (0.93, 1.07)
	GG	1091	1.02 (0.95, 1.09)	GG	182	1.11 (0.96, 1.29)
Total CHD	AA	273	1.07 (0.95, 1.20)	AA	2888	0.97 (0.94, 1.01)
	AG	1925	1.00 (0.96, 1.04)	AG	2684	1.00 (0.96, 1.04)
	GG	3947	1.03 (1.00, 1.06)	GG	573	0.99 (0.91, 1.08)
**b) Women**						
Ischaemic stroke	AA	298	0.99 (0.89, 1.11)	AA	3372	1.04 (1.00, 1.07)
	AG	2203	1.00 (0.96, 1.04)	AG	2996	1.00 (0.96, 1.04)
	GG	4592	0.98 (0.95, 1.01)	GG	725	1.01 (0.93, 1.08)
Intracerebral haemorrhage	AA	105	0.99 (0.82, 1.20)	AA	1184	1.03 (0.97, 1.09)
	AG	766	1.00 (0.93, 1.07)	AG	1073	1.00 (0.94, 1.06)
	GG	1654	1.03 (0.98, 1.09)	GG	268	1.04 (0.92, 1.17)
Total stroke	AA	442	1.00 (0.91, 1.10)	AA	4961	1.02 (1.00, 1.05)
	AG	3234	1.00 (0.97, 1.03)	AG	4472	1.00 (0.97, 1.03)
	GG	6826	1.00 (0.98, 1.03)	GG	1069	1.00 (0.94, 1.06)
Myocardial infarction	AA	57	1.15 (0.89, 1.50)	AA	575	0.97 (0.89, 1.05)
	AG	369	1.00 (0.90, 1.11)	AG	551	1.00 (0.92, 1.09)
	GG	820	1.05 (0.97, 1.13)	GG	120	0.90 (0.76, 1.08)
Total CHD	AA	324	0.99 (0.89, 1.11)	AA	3769	0.97 (0.94, 1.00)
	AG	2495	1.00 (0.96, 1.04)	AG	3593	1.00 (0.97, 1.03)
	GG	5360	1.01 (0.99, 1.04)	GG	817	0.95 (0.89, 1.02)

[1] Each group-specific CI (including that for AG, the reference group) reflects the variance of the log risk in that group, so any two-way comparison between AG and GG should take account of the variance of the log risk both in AG and in GG. The relative risks (RRs) are adjusted for age and area, so depend only on genotype.

Webfigure 6: Associations of *ALDH2*-rs671 with cardiovascular disease incidence

Analyses are adjusted for area and age. Reference = AG genotype. Results are plotted at the mean MALE alcohol intake of each genotype to facilitate the comparison of findings between men and women. The mean FEMALE intakes of rs671 AA/AG/GG are 0.6/1.9/5.1 g/week

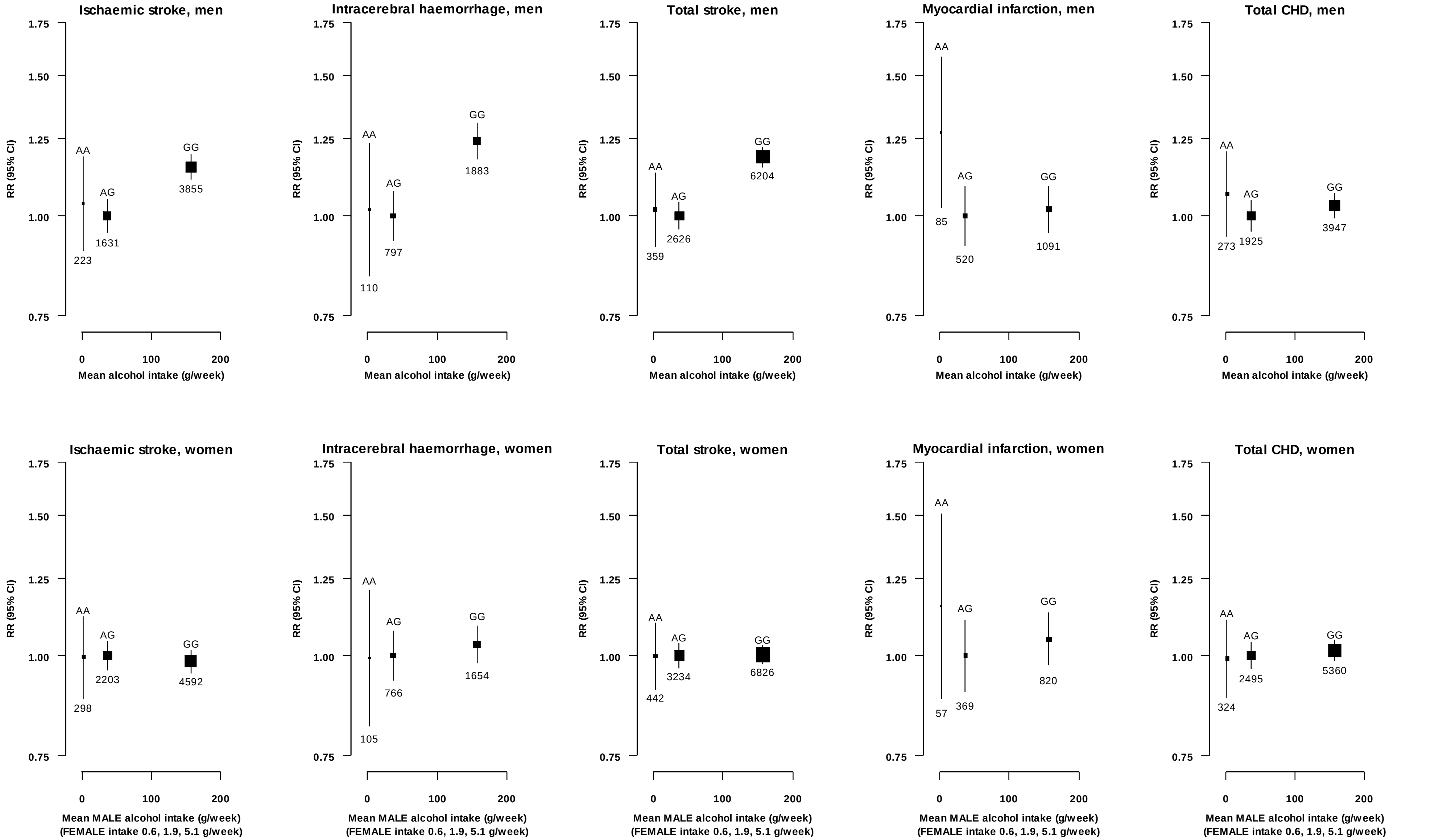

Webfigure 7: Associations of *ADH1B*-rs1229984 with cardiovascular disease incidence

Analyses are adjusted for area and age. Reference = AG genotype. Results are plotted at the mean MALE alcohol intake of each genotype to facilitate the comparison of findings between men and women. The mean FEMALE alcohol intakes of rs1229984 AA/AG/GG are 3.5/3.9/5.6 g/week

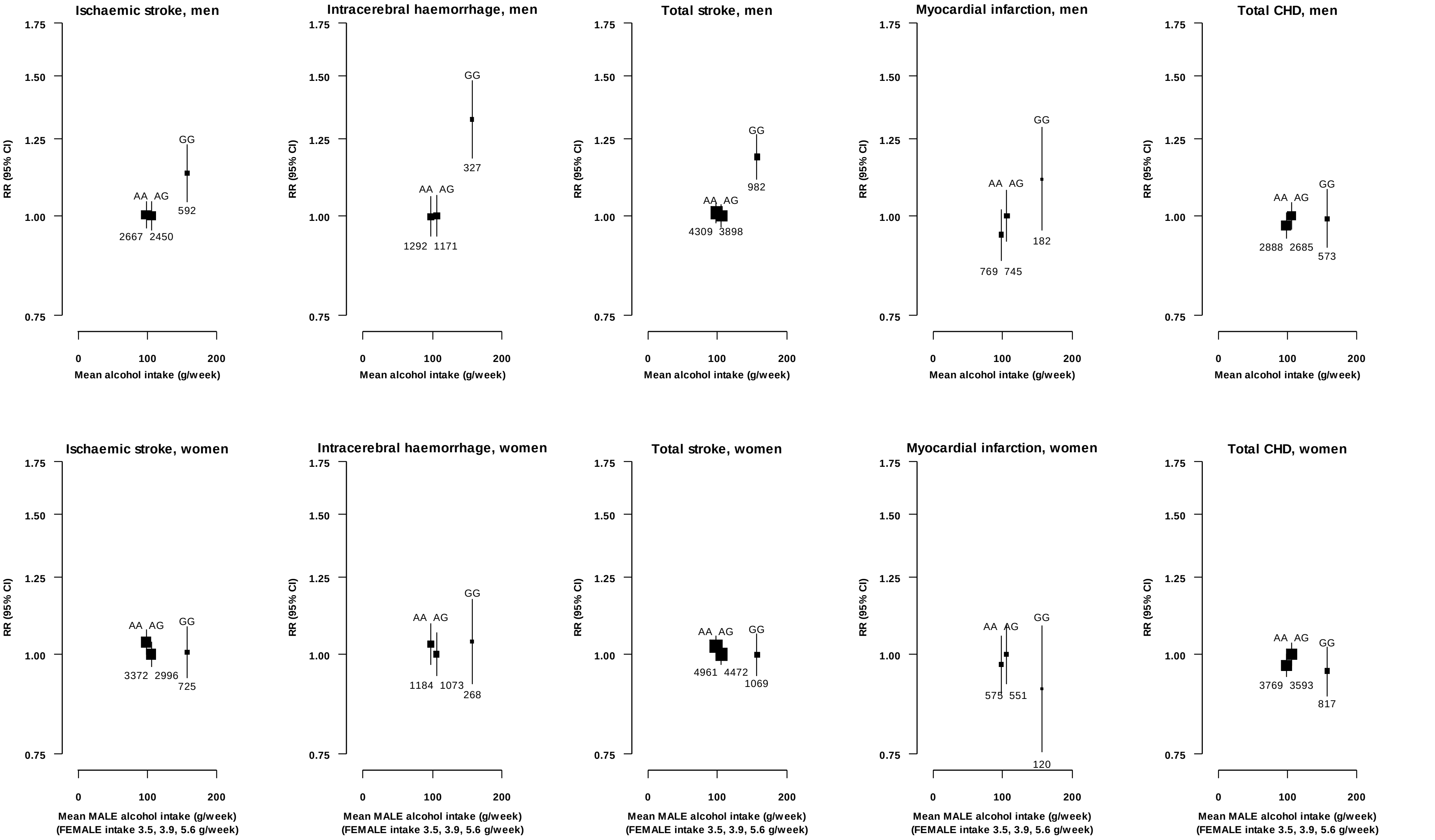

Webfigure 8: Ischaemic stroke: Results previously presented from (a) conventional and (b) genetic epidemiology; and Results expected from genetic epidemiology if moderate drinking is (c) protective and (d) not

Panel (c) describes what would be expected in genetic epidemiology if the U-shaped relationship with risk in non, occasional and current drinkers in Panel (a) is entirely causal, indicating a protective effect of occasional or moderate intake but thereafter an adverse effect of greater intake (with the few ex-drinkers given the risks of non-drinkers).

Panel (d) describes what would be expected in genetic epidemiology if the relationship with risk in current drinkers in Panel (a) is entirely causal, and extends down to zero intake for other men. Panels (c) and (d) take the relationship of intake to risk among current drinkers to be log-linear, and as steep as the line fitted to current drinkers in Panel (a).

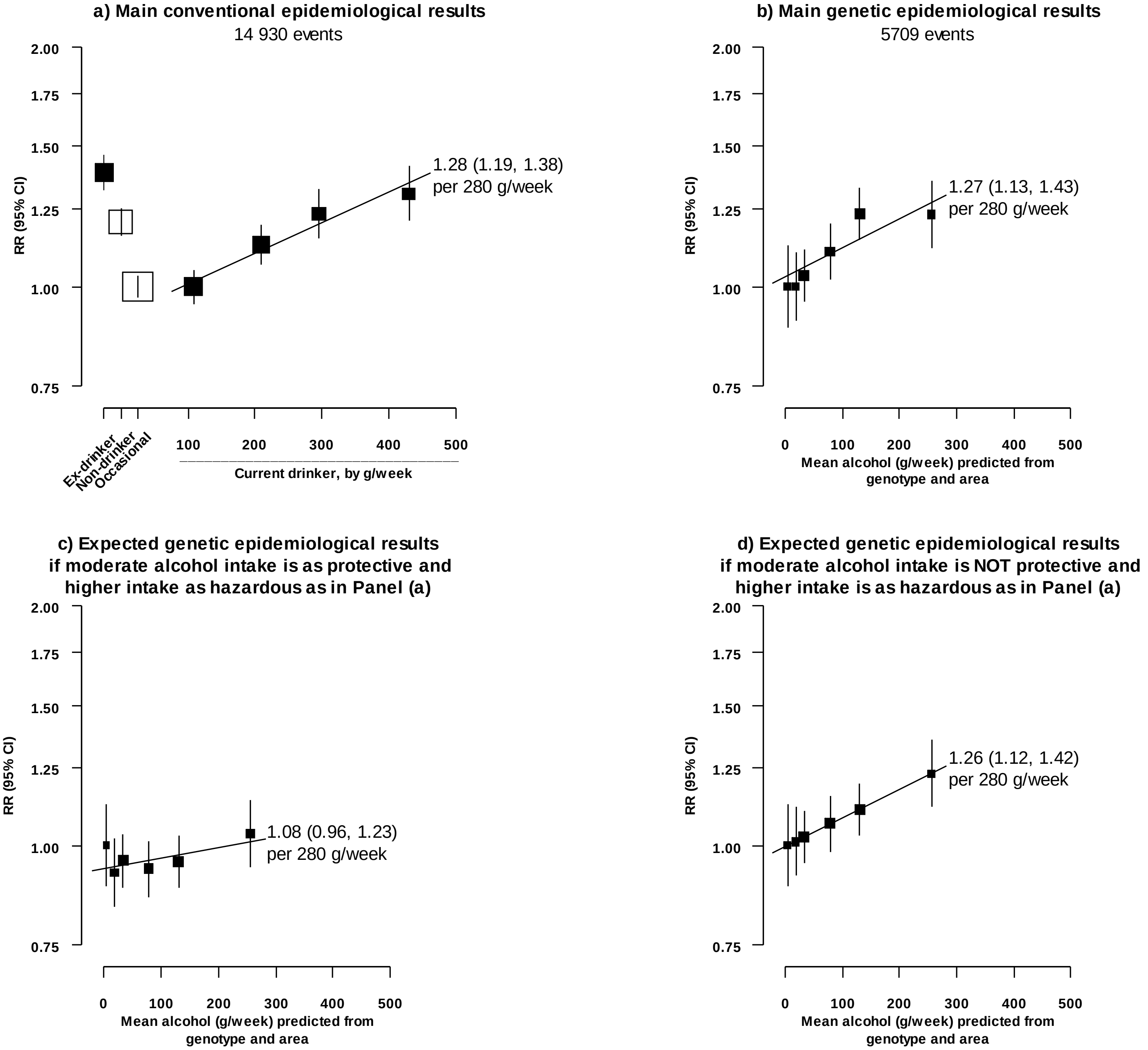

Comment

If moderate intake were protective but higher intake hazardous, then on going from categories C1-C6 the risks would *decrease* due to less abstinence, but *increase* due to higher consumption per drinker.

Details: Webtable 8 (p17 of this Appendix) shows that, for the categories C1-C6 used in genetic epidemiology,

in C1, ~2/3 were never-drinkers and almost all others were only occasional drinkers;
in C2-C3, ~1/3 were never-drinkers and half were only occaisonal drinkers;
in C4-C5, ~10% were never-drinkers, 40% occasional drinkers and 50% current drinkers;
in C6, ~60% were current drinkers, with intake per drinker much higher than in C4-C5.

Webfigure 9: Intracerebral haemorrhage: Results previously presented from (a) conventional and (b) genetic epidemiology; and Results expected from genetic epidemiology if moderate drinking is (c) protective and (d) not

Panel (c) describes what would be expected in genetic epidemiology if the U-shaped relationship with risk in non, occasional and current drinkers in Panel (a) is entirely causal, indicating a protective effect of occasional or moderate intake but thereafter an adverse effect of greater intake (with the few ex-drinkers given the risks of non-drinkers).

Panel (d) describes what would be expected in genetic epidemiology if the relationship with risk in current drinkers in Panel (a) is entirely causal, and extends down to zero intake for other men. Panels (c) and (d) take the relationship of intake to risk among current drinkers to be log-linear, and as steep as the line fitted to current drinkers in Panel (a).

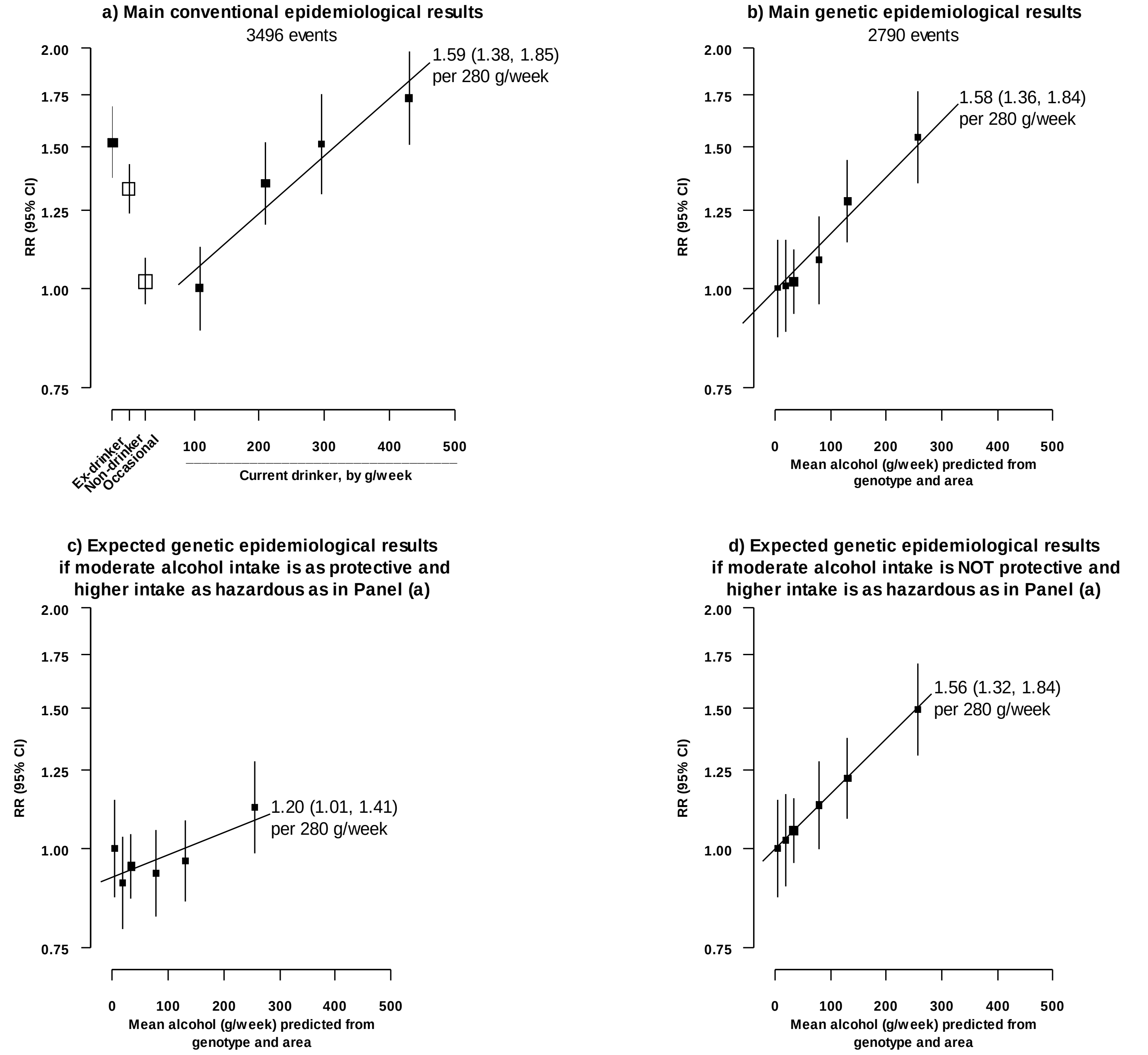

Comment

If moderate intake were protective but higher intake hazardous, then on going from categories C1-C6 the risks would *decrease* due to less abstinence, but *increase* due to higher consumption per drinker.

Details: Webtable 8 (p17 of this Appendix) shows that, for the categories C1-C6 used in genetic epidemiology,
in C1, ~2/3 were never-drinkers and almost all others were only occasional drinkers;
in C2-C3, ~1/3 were never-drinkers and half were only occaisonal drinkers;
in C4-C5, ~10% were never-drinkers, 40% occasional drinkers and 50% current drinkers;
in C6, ~60% were current drinkers, with intake per drinker much higher than in C4-C5.

Webfigure 10: Myocardial infarction: Results previously presented from (a) conventional and (b) genetic epidemiology; and Results expected from genetic epidemiology if moderate drinking is (c) protective and (d) not

Panel (c) describes what would be expected in genetic epidemiology if the U-shaped relationship with risk in non, occasional and current drinkers in Panel (a) is entirely causal, indicating a protective effect of occasional or moderate intake but thereafter an adverse effect of greater intake (with the few ex-drinkers given the risks of non-drinkers).

Panel (d) describes what would be expected in genetic epidemiology if the relationship with risk in current drinkers in Panel (a) is entirely causal, and extends down to zero intake for other men. Panels (c) and (d) take the relationship of intake to risk among current drinkers to be log-linear, and as steep as the line fitted to current drinkers in Panel (a).

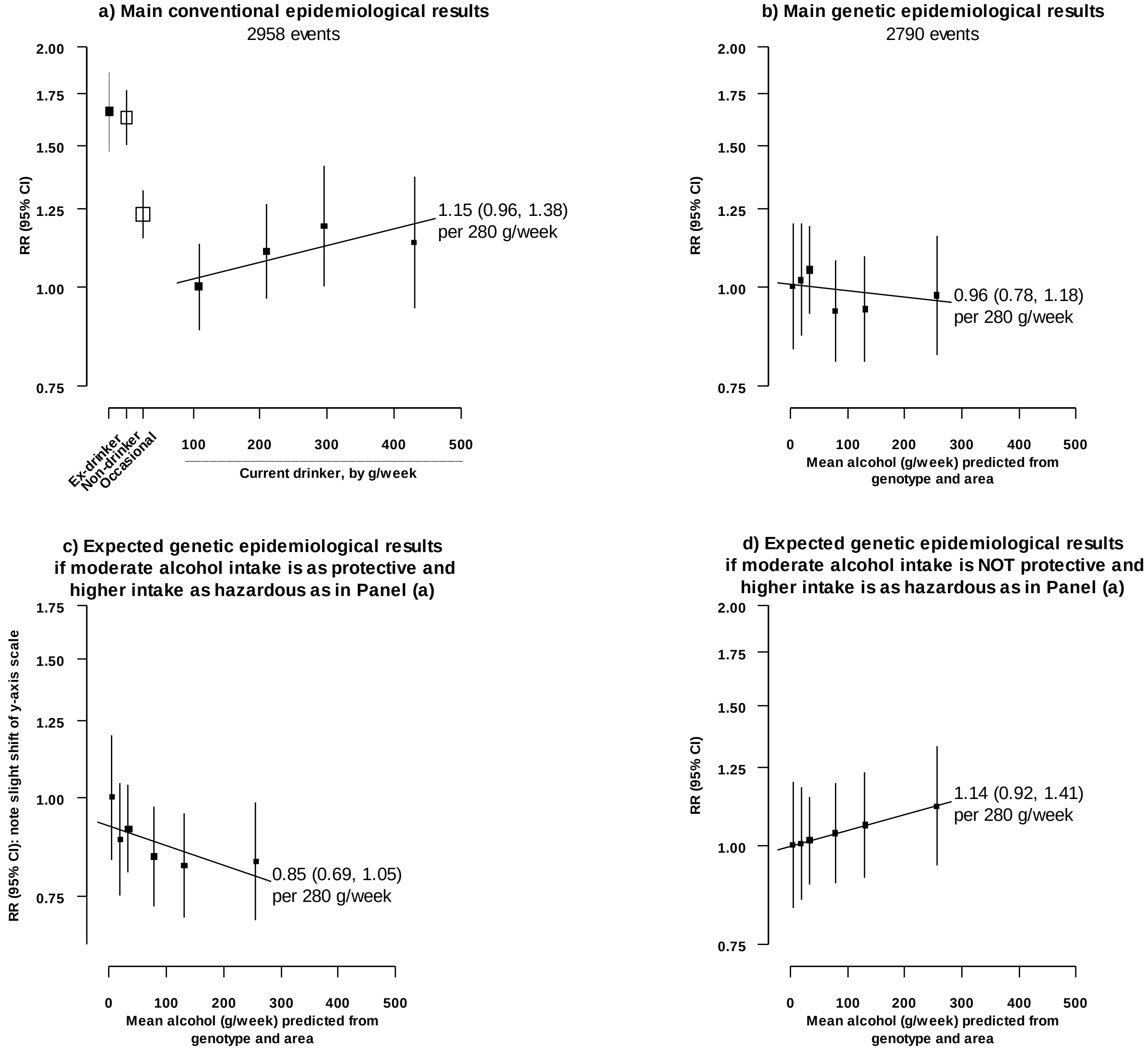

Comment

If moderate intake were protective but higher intake hazardous, then on going from categories C1-C6 the risks would *decrease* due to less abstinence, but *increase* due to higher consumption per drinker.

Details: Webtable 8 (p17 of this Appendix) shows that, for the categories C1-C6 used in genetic epidemiology,
in C1, ~2/3 were never-drinkers and almost all others were only occasional drinkers;
in C2-C3, ~1/3 were never-drinkers and half were only occaisonal drinkers;
in C4-C5, ~10% were never-drinkers, 40% occasional drinkers and 50% current drinkers;
in C6, ~60% were current drinkers, with intake per drinker much higher than in C4-C5.

Webfigure 11: Genotypic associations of *ALDH2*-rs671 with ischaemic stroke, intracerebral haemorrhage and myocardial infarction (a) observed, (b) expected if moderate drinking is protective, and (c) expected if it is not

Panel (b) describes what would be expected if the U-shaped relationships with risk among non, occasional and current drinkers in conventional epidemiology are causal, indicating a protective effect of occasional or moderate intake but thereafter an adverse effect of greater intake (with the few ex-drinkers given the risks of non-drinkers).

Panel (c) describes what would be expected if the adverse effect in current drinkers in conventional epidemiology is causal, and it extends down to zero intake for other men. Panels (b) and (c) both take the relationship of intake to risk in current drinkers to be log-linear, and as steep as the line fitted to current drinkers in conventional epidemiology.

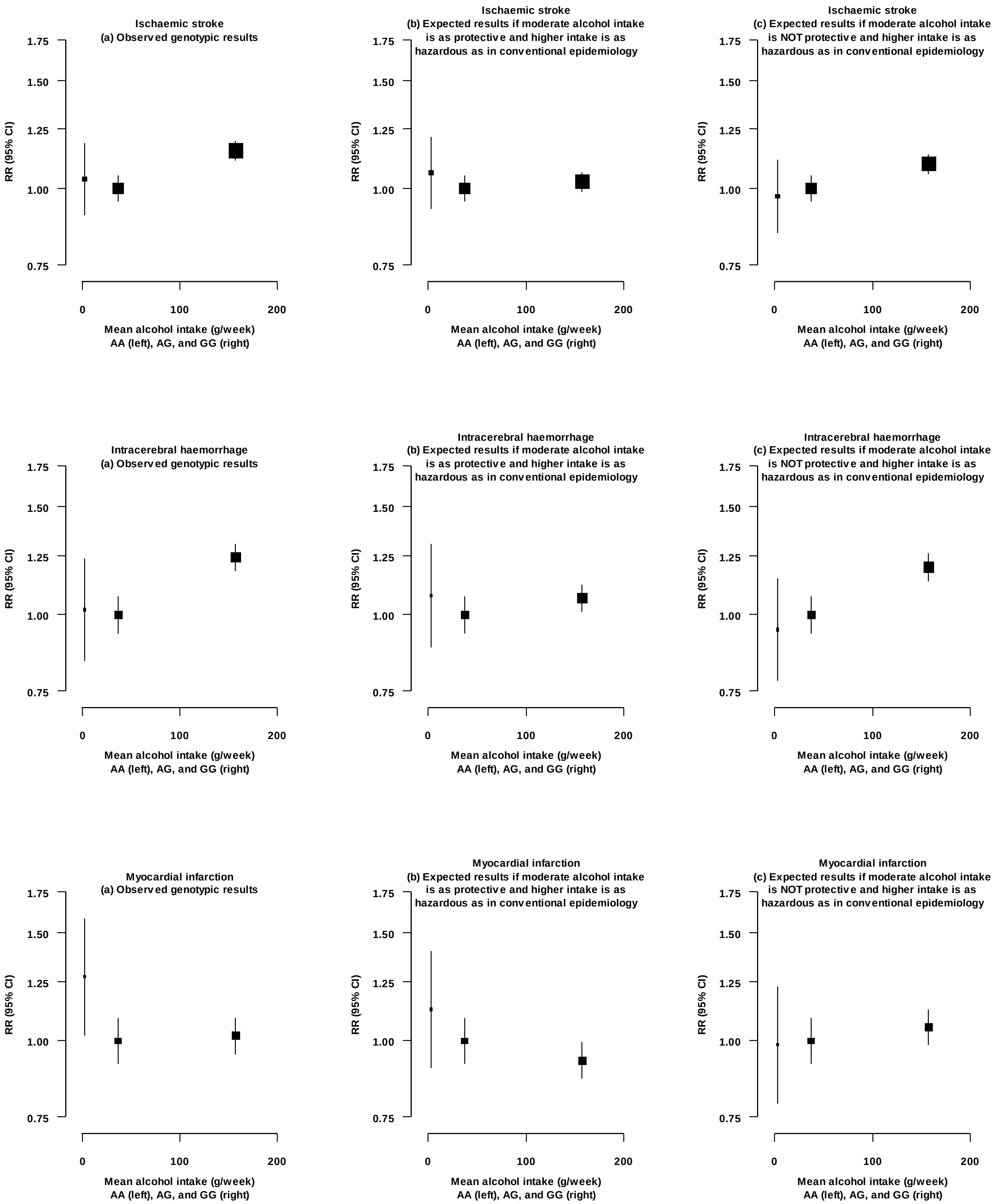

Webfigure 12: Genotypic associations of *ADH1B*-rs1229984 with ischaemic stroke, intracerebral haemorrhage and myocardial infarction (a) observed, (b) expected if moderate drinking is protective, and (c) expected if it is not

Panel (b) describes what would be expected if the U-shaped relationships with risk among non, occasional and current drinkers in conventional epidemiology are causal, indicating a protective effect of occasional or moderate intake but thereafter an adverse effect of greater intake (with the few ex-drinkers given the risks of non-drinkers).

Panel (c) describes what would be expected if the adverse effect in current drinkers in conventional epidemiology is causal, and it extends down to zero intake for other men. Panels (b) and (c) both take the relationship of intake to risk in current drinkers to be log-linear, and as steep as the line fitted to current drinkers in conventional epidemiology.

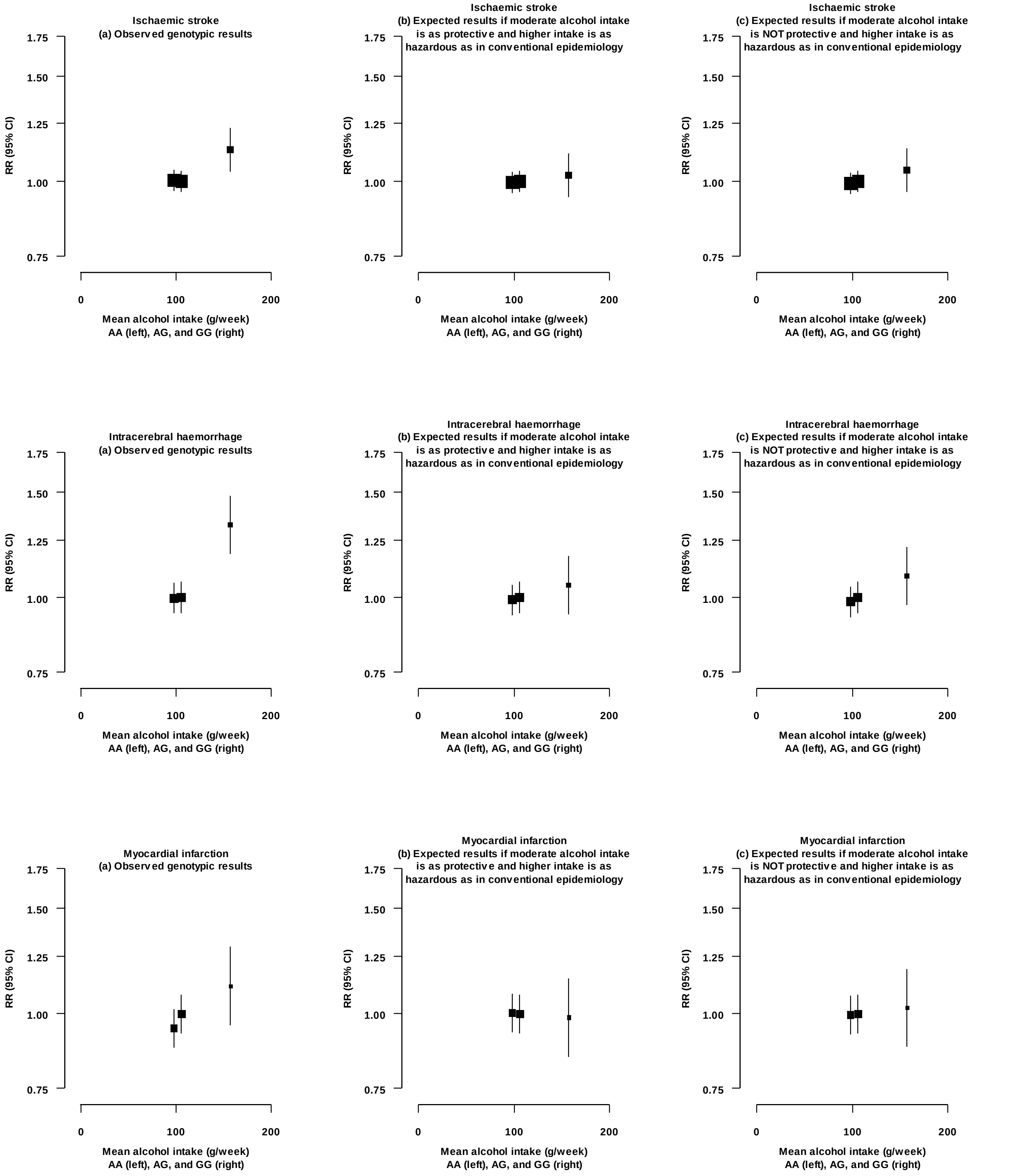

Supplement: Supplementary appendix [file mmc1.pdf]
